# 2-Aminothiophene Derivatives—New Drug Candidates Against Leishmaniasis: Drug Design, Synthesis, Pharmacomodulation, and Antileishmanial Activity

**DOI:** 10.3390/ph18010125

**Published:** 2025-01-17

**Authors:** Rodrigo Santos Aquino de Araújo, Vitória Gaspar Bernardo, Robert da Silva Tibúrcio, Danilo Cesar Galindo Bedor, Michel Leandro de Campos, Roberto Pontarolo, Julyanne Maria Saraiva de Sousa, Klinger Antonio da Franca Rodrigues, Marcus Tullius Scotti, Anuraj Nayarisseri, Pascal Marchand, Francisco Jaime Bezerra Mendonça-Junior

**Affiliations:** 1 Laboratory of Synthesis and Drug Delivery, Department of Biological Sciences, State University of Paraíba, João Pessoa 58071-160, Brazil; rodrigobiologojp@gmail.com (R.S.A.d.A.); vit.gaspar.bernardo@gmail.com (V.G.B.); 2 Postgraduate Program in Natural and Synthetic Bioactive Products, Federal University of Paraíba, João Pessoa 58051-900, Brazil; mtscotti@gmail.com; 3 Laboratory of Planning and Synthesis in Medicinal Chemistry, Pharmaceutical Sciences Department, Federal University of Pernambuco, Recife 50740-520, Brazil; robertstiburcio@gmail.com; 4 Pharmaceutical and Cosmetic Development Center (NUDFAC), Department of Pharmaceutical Science, Federal University of Pernambuco, Recife 50740-520, Brazil; danilo.bedor@ufpe.br; 5 Health Sciences Institute, Federal University of Mato Grosso (UFMT), Sinop 78550-000, Brazil; michel.campos@ufmt.br; 6 Departamento de Farmácia, Universidade Federal do Paraná, Av. Prefeito Lothário Meissner 632, Curitiba 80210-170, Brazil; pontarolo@ufpr.br (R.P.); klinger@ufdpar.edu.br (K.A.d.F.R.); 7 Infectious Disease Laboratory, Campus Ministro Reis Velloso, Federal University of Parnaíba Delta, Parnaíba 64202-020, Brazil; julyannemss@ufdpar.edu.br; 8 In Silico Research Laboratory, Eminent Biosciences, Mahalakshmi Nagar, Indore 452010, India; anuraj@eminentbio.com; 9 Nantes Université, Cibles et médicaments des infections et de l’immunité, IICiMed, UR 1155, F-44000 Nantes, France; pascal.marchand@univ-nantes.fr

**Keywords:** leishmaniasis, 2-aminothiophene, drug design, bioisosterism, pharmacomodulation

## Abstract

**Background/Objectives**: Leishmaniasis is one of the 20 Neglected Tropical Diseases according to the WHO, affecting approximately 12 million people in four continents, generating serious public health problems. The lack of therapeutic options, associated with toxicity and the emergence of resistance to the few available drugs, makes it urgent to develop new drug options. In this context, the aims of this work are to expand the knowledge about the pharmacophore group responsible for the antileishmanial potential of 2-aminothiophene derivatives. Thus, new compounds were synthesized containing chemical modifications at the C-3, C-4, and C-5 positions of the 2-aminothiophene ring, in addition to the S-Se bioisosterism. **Methods**: Dozens of 2-AT and 2-aminoselenophen (2-AS) derivatives were sequentially synthesized through applications of the Gewald reaction and were then evaluated in vitro for their activities against *L. amazonensis* and for cytotoxicity against macrophages. **Results**: Several series of compounds were synthesized, and it was possible to identify some substitution patterns favorable to the activity generating compounds with IC_50_ values below 10 µM, such as the non-essentiality of the presence of a carbonitrile group at C-3; the importance of the presence and size of cycloalkyl/piperidinyl chains at C-4 and C-5 in modulating the activity; and the increase in activity without affecting the safety of the S/Se bioisosteric substitution. **Conclusions**: Taken together, these findings reaffirm the great potential of 2-aminothiophenes to generate antileishmanial drug candidates and offers contributions to the drug design of compounds with an even more promising profile for the problem of leishmaniasis.

## 1. Introduction

Leishmaniasis, a group of vector-borne diseases caused by protozoan parasites of the genus *Leishmania*, represents a significant public health challenge in many tropical and subtropical regions. It is considered one of the 20 Neglected Tropical Diseases (NTDs) according to the World Health Organization (WHO) [1], and it presents three clinical manifestation forms: visceral, mucocutaneous, and cutaneous, which manifest endemically in approximately 100 countries in four continents, with an estimated prevalence of 12 million cases, and with a risk of infecting 1 in 8 people worldwide [1,2,3].

The clinical forms of leishmaniasis differ in terms of immunopathogenicity and morbimortality rates, and depend on the infecting *Leishmania* species [4]. However, transmission to the mammalian hosts (including humans and dogs) occurs through the bites from infected female sand flies, particularly *Phlebotomus* in the Old World and *Lutzomyia* in the Americas [5,6].

Cutaneous leishmaniasis (CL) is caused by around 20 species of *Leishmania*, especiallly *L. major*, *L. tropica*, *L. amazonensis*, *L. braziliensis*, *L. guyanensis*, and *L. mexicana* [3,6]. Although it is not the most severe form of the disease (which is fatal in the absence of treatment), it stands out as the most prevalent form and also the one that causes the greatest social stigma due to the deformities and disfiguring scars caused by ulcerative skin lesions, physically and psychologically affecting patients [7,8].

Despite all these global problems, and the great efforts made by the scientific community to control this disease, there are currently no effective vaccines available [9,10,11], the vector control remains a considerable challenge [12], and chemotherapy treatment options have not evolved in recent decades [3,13,14].

The limited and old therapeutic arsenal available continues to be based on using pentavalent antimonials (Glucantime and Pentostam) as the first-line treatment, along with liposomal amphotericin B (AmBisome^®^), pentamidine, paromomycin, and the only oral drug miltefosine (IMPAVIDO^®^). These drugs have several limitations related to toxicity, serious side effects, and/or long half-life, high costs, and long treatment regimens, in addition to the fact that their extensive use has promoted the emergence of resistant strains [3,13,15]. These facts highlight the need to discover and develop new chemotherapies that may be more effective and safer against leishmaniasis [3,16,17].

In this context, our research group has been interested in the developing new antileishmanial drug candidates based on 2-aminothiophenic prototypes. 2-Aminothiophene (2-AT) derivatives are privileged structures widely used in medicinal chemistry for designing bioactive compounds and are the subject of several recent review articles [18,19,20,21].

**Figure 1:**
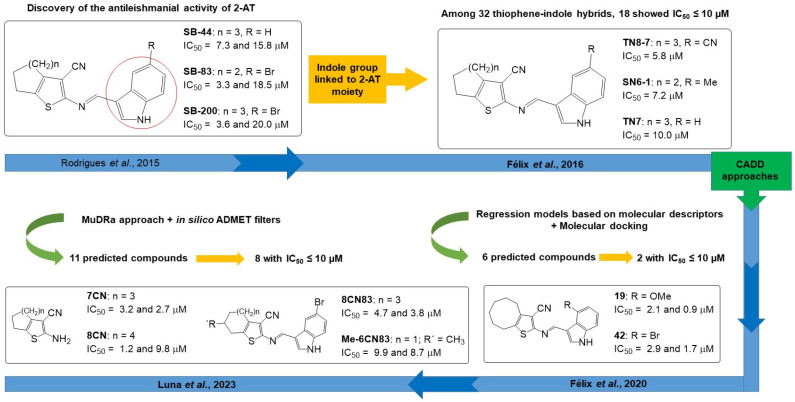
Timeline of the drug design and development process of 2-aminothiophenes with antileishmanial activity. Within the squares are the most active compounds of each study. The IC_50_ values represent the 50% inhibition values against the promastigotes and amastigotes forms of *L. amazonensis*. The references cited in the figure correspond to the following references in the text: Rodrigues et al., 2015 [22]; Félix et al., 2016 [24]; Félix et al., 2020 [25]; and Luna et al., 2023 [26].

Regarding leishmaniasis, one of our first studies reported the antileishmanial activity of 10 2-AT against *L. amazonensis* [22]. In this first study, three compounds (**SB-44**, **SB-83**, and **SB-200**) (Figure 1) stood out, with IC_50_ values of 7.37, 3.37, and 3.65 μM against promastigotes and EC_50_ of 15.82, 18.5, and 20.09 μM against amastigotes, respectively, being much more active than the reference drug Meglumine antimoniate (pentavalent antimoniate). This study indicated that antileishmanial activity can be related an immunomodulatory mechanism, in addition to signaling that the presence of the indole group linked to the 2-amino portion is favorable to the activity.

The sequence of this study resulted in an in vivo oral evaluation of the compound **SB-83** in a model of *L. amazonensis* infection in Swiss mice. The results after 7 weeks of treatment with **SB-83** at concentrations of 50 and 200 mg/kg were more effective than meglumine antimoniate (via i.p at 100 mg/kg), reducing paw lesion size and decreasing the parasite load in the reservoirs without causing significant clinical changes in the biochemical, hematological, and histopathological parameters [23].

These findings motivated the synthesis of a series of 32 2-aminothiophene-indole hybrids, in which the size of the cycloalkyl ring fused to the C-4 and C-5 positions of the thiophene ring (5 to 8-membered rings) was modified, as well as the positions (C4′, C5′, and C7′) and substituents (methyl, methoxyl, cyano, nitro) attached to the indole ring [24]. More than half of these hybrid compounds (18 compounds) exhibited pronounced anti-promastigote activity with IC_50_ values below 15.0 μM, being more active and less toxic than the reference drugs (tri- and pentavalent antimonials, with IC_50_ values of 12.7 and 87.7 μM, respectively). The most active compounds were **TN8-7**, **TN6-1**, and **TN7** (Figure 1), with IC_50_ values of 5.8, 7.2, and 10.0 μM, respectively. These compounds were also shown to be able to inhibit the growth of *L. amazonensis* trivalent antimony (Sb^+III^)-resistant strain (for example, for compound **TN8-7**: IC_50_ values of 7.8 (resistant strain) and 5.8 μM (sensitive strain), but without a statistically significant difference), showing that it may be a therapeutic alternative for resistant strains.

The results of this study indicate that the nature of the substituent and its position in the indole ring promote the modulation of anti-*Leishmania* activity, with position 5′ being a privileged position. It was additionally observed that the increase in the size of the cycloalkyl ring fused to the thiophene ring is favorable to the activity, with cycloocta[*b*]thiophene series being the one with the best profile.

In turn, two independent studies with different approaches using distinct computer-aided drug design (CADD) tools were conducted as a follow-up to these studies and aiming at a more quantitative approach to identify 2-aminothiophenes as anti-*Leishmania* drug candidates.

In the first of these, Félix et al. (2020) [25] used 92 2-AT to build regression models based on molecular descriptors generated by the DRAGON 7.0 program, which were statistically analyzed using MobyDigs v. 1.1. Then, the 30 molecules with the best predicted IC_50_ values (pIC_50_) were selected from these models, with which molecular docking studies were performed against three *Leishmania* enzymes: arginase, glycerol-3-phosphate dehydrogenase, and pyruvate kinase. After the docking studies, the six compounds with the greatest potential for anti-*Leishmania* activity were selected, synthesized, and evaluated against *L. amazonensis*. Of the six predicted and synthesized compounds, two of them, compounds **19** and **42**, showed pronounced antileishmanial activity against the two evolutionary forms of the parasite, respectively presenting IC_50_ values of 2.16 and 2.97 μM (against promastigotes) and of 0.9 and 1.71 μM (against axenic amastigotes), with selectivity indexes (SI) of 52 and 75, and therefore, being more active and less toxic than the reference drug meglumine antimoniate (Sb^+5^) (IC_50_ = 70.33 and 2.77 μM against promastigotes and amastigotes, respectively; SI = 1.01). These two molecules have in common the presence of the cycloocta[*b*]thiophene ring, as well as a substituent linked to the 4′ position of the indole ring (4′-OMe for **19** and 4′-Br for **42**) (Figure 1).

In the second study, Luna et al. (2023) [26] used the Multi-Descriptor Read Across (MuDRa) approach combined with in silico ADMET and pharmacokinetics studies using the online pkCSM server (http://biosig.unimelb.edu.au/pkcsm/) and the OSIRIS Data Explorer and DataWarrior version 5.5 software to perform a virtual screening using a dataset of 1862 compounds extracted from the ChEMBL database, in order to select 2-AT with potential anti-*L. amazonensis* activity. The result of the virtual screening predicted 11 2-aminothiophene with a desirable drug likeness profile and with a probability greater than 70% of inhibiting the two evolutionary forms of the parasite. All the compounds were synthesized, and eight of them (Figure 1) showed to be active against at least against one of the evolutionary forms of the parasite, with IC_50_ values lower than 10 μM, being more active than the reference drug meglumine antimoniate. The structure–activity relationship (SAR) study revealed: (1) the presence of indole substituents linked to the amino position of the 2-AT, especially 5-bromoindole, contributes to, but is not essential for, the antileishmanial activity, and (2) the Gewald adducts themselves possess antileishmanial potential.

This set of results highlights the unquestionable antileishmanial potential of 2-amino-thiophene derivatives and motivated us to expand the investigation of the pharmacophoric skeleton through the design, synthesis, and bio-evaluation of new synthetic derivatives containing chemical modifications at the C-3, C-4, and C-5 positions of the 2-aminothiophenic ring, in addition to the S-Se bioisosteric substitution at the heteroatom level.

## 2. Results and Discussion

### 2.1. Synthesis

First, variations in the classical Gewald reaction [27,28] were employed to synthesize the 2-aminothiophene (2AT) and 2-aminoselenophene (2-AS) derivatives of the present work.

**Scheme 1:**
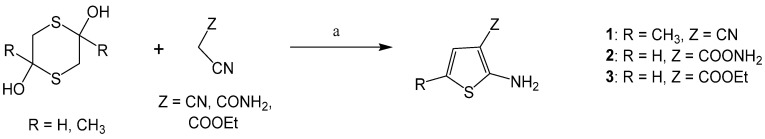
Synthesis of 2-AT using 1,4-dithiane-2,5-diol derivatives: (**a**) morpholine, ethanol, reflux, 2–3 h.

Thus, to synthesize compounds **1**–**3** (4-unsubstitued 2-AT), the fourth version of the Gewald reaction was used. 1,4-Dithiane-2,5-diol derivatives were condensed with α-activated acetonitrile (malononitrile, ethyl cyanoacetate, or cyanoacetamide) in a basic ethanolic medium (Scheme 1).

**Scheme 2:**
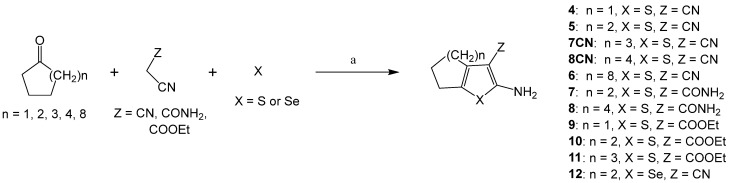
Synthesis of 2-ATs and 2-AS following the Gewald methodology: (**a**) morpholine, ethanol, reflux, 4–48 h.

**Scheme 3:**
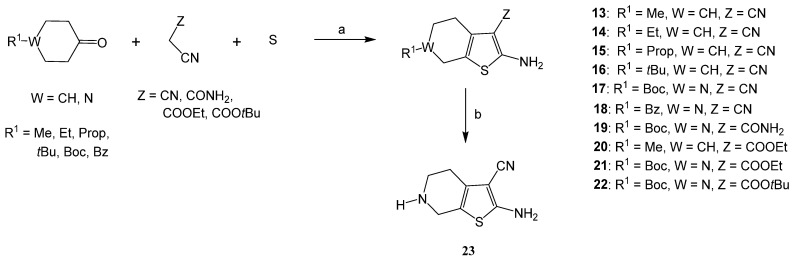
Synthesis of 2-AT following the Gewald methodology starting from 4-alkyl-cyclohexanones, 4-N-benzyl-piperidone, and 4-Boc-piperidone: (**a**) morpholine, ethanol, reflux, 3–22 h; (**b**) CH_2_Cl_2_/TFA (8:2), 0–5 °C, 20 min.

All other Gewald adducts were obtained by reacting different α-activated acetonitrile (malononitrile, ethyl- and tert-butyl-cyanoacetate, or cyanoacetamide) with cycloalkylketones (cyclopentanone, cyclohexanone, cycloheptanone, cyclooctanone, and cycloundecanone) for compounds **4**–**12**, **7CN**, and **8CN**; 4-alkyl-cyclohexanones for compounds **13**–**16** and **20**; 4-Boc-piperidone for compounds **17**, **19**, **21**, and **22**; 4-N-benzyl-piperidone for compound **18**; and sulfur or selenium in a basic ethanolic medium. Compound **23** was obtained by the Boc deprotection of compound **17** using a mixture of dichloromethane and trifluoroacetic acid (8:2) (Scheme 2 and Scheme 3).

**Scheme 4:**
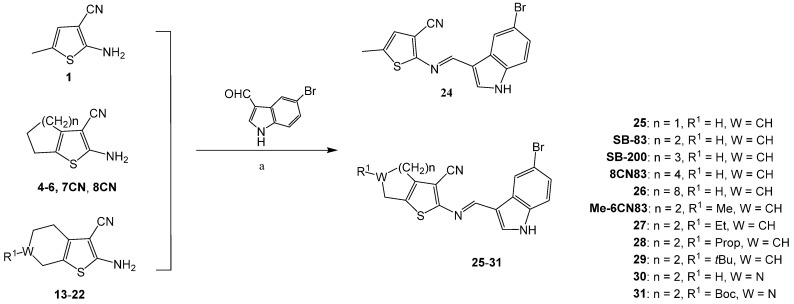
Synthesis of 2-aminothiophene-5-bromoindole hybrids: (**a**) ethanol, acetic acid, reflux, 2–24 h.

**Scheme 5:**
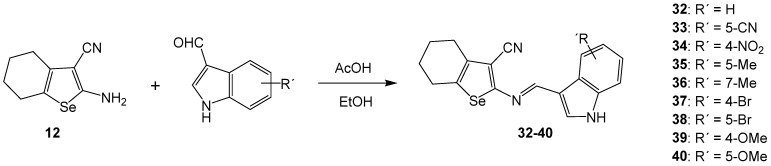
Synthesis of 2-aminoselenophene-indole hybrids: ethanol, acetic acid, reflux, overnight.

The condensation reactions of 5-bromoindole-3-carboxaldehyde with 2-aminothiophenes yielded the 2-aminothiophene-indole hybrids **24**-**31**, **SB-83**, **SB-200**, **8CN83**, and **Me-6CN83** (Scheme 4), and the condensation of substituted-indole-3-carboxaldehyde with 2-aminoselenophene **12** yielded the 2-aminoselenophene-indole hybrids **32**–**40** (Scheme 5), according to a previously described procedure [22,26].

After the purification and determination of the physicochemical characteristics, all the new compounds had their structures confirmed by Nuclear Magnetic Resonance (^13^C NMR or DEPT and ^1^H NMR), and by High Resolution Mass Spectra (HRMS) (all spectra are available in the Supplementary Materials). The previously described compounds were resynthesized and their structures were confirmed by comparing their physicochemical characteristics and ^1^H NMR.

### 2.2. Pharmacomodulation Study

#### 2.2.1. Effect of Cycloalkyl Substituent Attached to C-4/C-5

Based on our previous results, we decided to first evaluate the influence of alkyl and cycloalkyl substituents linked at the C-4 and C-5 positions of the thiophene ring on the anti-promastigote and anti-amastigote activities of *L. amazonensis*, keeping the 5′-bromo-indole substituent fixed. With this aim, 12 compounds were synthesized and evaluated. Their chemical structures and IC_50_ and CC_50_ values are presented in Table 1.

According to Table 1, all compounds considered active (IC_50_ ≤ 10.0 µM) against at least one of the evolutionary forms of *L. amazonensis* showed superior antileishmanial activity to pentavalent antimony (Meglumine antimoniate) and inferior to Amphoterecin B (AmpB). Despite the lower activity when compared to AmpB, all active compounds were much less cytotoxic and had a greater therapeutic safety margin.

This first set of results confirms the results initially observed in the study conducted by Luna et al. (2023) [26] and shows that the nature of the substituent linked to the C-4 and C-5 positions of the thiophene ring modulates the antileishmanial activity (anti-promastigote and anti-amastigote) of this class of compounds.

The exclusion of the cycloalkyl ring linked to the C-4 and C-5 positions, as observed in compound **24** (which a methyl radical at C-5), results in the loss of antileishmanial activity, indicating that the presence of cycloalkyl rings appears to be important for the activity.

Regarding the nature of the cycloalkyl fused to the thiophene ring, the size of the ring also seems to indicate ideal limits for maintaining antileishmanial activity.

Smaller, five-membered rings, as observed in compound **25**, and larger, 12-membered rings, as observed in **26**, were inactive against the two evolutionary forms of the parasite. However, 6-, 7-, and 8-membered rings, as respectively observed in **SB-83**, **SB-200** (previously published in Rodrigues et al. (2015) [22]), and **8CN83**, are active.

Considering the antileishmanial potential against the two evolutionary forms of the parasite, the compound **8CN83** (8-membered ring) proved to be more promising, presenting the lowest IC_50_ value against the amastigote form (IC_50_ = 3.8 µM), associated with the absence of cytotoxicity up to 100 µM. Here, it is important to mention that this activity pattern corroborates the results found by Félix et al. (2016) [24]. We believe that these limits may be related to an optimal size range to bind to potential biological targets and/or to optimal solubility and/or logP limits.

The bioisosteric substitution of a methylene (-CH_2_-) of the compound **SB-83** (6-membered ring) by an amine (-NH), in **30**, was also negative and resulted in the complete loss of activity, indicating that the presence of a group presenting a hydrogen bond donor in this portion of the molecule does not seem to be favorable to antileishmanial activity.

Finally, evaluating the influence of the presence of alkyl substituents (methyl (**Me-6CN83**), ethyl (**27**), propyl (**28**), and tert-butyl (**29**)) at the C-6 position of the tetrahydrocyclobenzo[b]thiophene (**SB83**) demonstrates that the substitution is tolerable, but not positive. Half of the compounds (**Me-6CN83** and **28**) showed antileishmanial activity with IC_50_ values below 10 µM, but with higher values than the unalkylated cycloalkyl compound **SB-83**. Similarly, the presence of the Boc group in **31** did not result in an increase in the activity of its N-unsubstituted analogous (compound **30**).

#### 2.2.2. Chemical Stability Assay

The promising results obtained for some of these 2-AT derivatives encouraged conducting chemical stability tests on these compounds in order to evaluate the identification of potential metabolites (among others). Thus, five 2-AT derivatives possessing anti-Leishmania activity (**TN8-1**, **TN8-2**, **SB-44**, **SB-83**, and **SB-200**) were selected to perform chemical stability assays.

The preliminary in silico analysis using the Zeneth Lhasa software (Version 7.1.3), demonstrated that the imine was a hydrolysable group at both pH 1.2 and 7.4 (as expected), but with more pronounced effects at the acidic pH.

The formation of degradation products was observed and confirmed by conducting chemical stability assays in buffer solutions at pH 1.2 and 7.4. This behavior is consistent with that reported for imines. Next, the stability of the imine bond between amphotericin and a polyethylene glycol carrier was observed at the alkaline pH, with a loss of only 5% over 24 h. Conversely, there was a rapid release of free amphotericin at pH 5.5 due to the cleavage of the imine bond, with a half-life of less than 5 min for all tested derivatives [29]. However, it was not possible to calculate the decay half-life in our assay as the reaction occurred immediately, probably due to the even lower pH.

**Figure 2:**
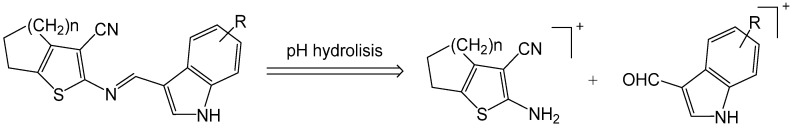
Synthetic precursors identified by UPLC-MS/MS in the chemical stability assays in buffer solutions at pH 1.2 and 7.4.

Metabolites (degradation products) were then identified by UPLC-MS/MS (Ultra-High Performance Liquid Chromatography coupled to high Resolution Mass Spectrometry), where the mass/charge (*m*/*z*) peaks corresponding to the synthetic precursors 2-AT (Gewald aducts) and substituted indoles were detected (as shown in Figure 2).

#### 2.2.3. Effect of the Absence of the Indole Substituent

Based on these stability results, and considering that all 2-AT derivatives described in Table 2 were synthesized from the same indole aldehyde (5-bromoindole), the observed difference in IC_50_ values indicates that the nature of the thiophene ring exerts influence on modulating antileishmanial activity.

Therefore, we decided to investigate whether Gewald adducts (simpler 2-amino-thiophenes) could also have anti-Leishmania properties. The confirmation of this investigation would allow us to provide evidence that 2-aminothiophene-indole hybrids could act as reciprocal prodrugs.

Thus, the Gewald adducts **4, 5**, **7CN**, **8CN**, **13**-**18**, and **23** were synthesized and had their anti-Leishmania activity evaluated against *L. amazonensis* (Table 2).

The results described in Table 2 confirm most of the results observed in Table 1 and largely validate the hypothesis that 2-aminothiophene-indole hybrids can effectively be prodrugs.

Table 2 shows that the absence of a cycloalkyl ring fused to the thiophene ring at the C-4 and C-5 positions, as observed in **1**, results in the loss of antileishmanial activity. It is also confirmed that increasing the cycloalkyl ring size promotes the antileishmanial activity, with compound **8CN** (8-membered rings) having the best profile, presenting IC_50_ values equal to 1.2 and 2.6 µM against promastigotes and amastigotes, respectively.

Table 2 also validates that the presence of alkyl substituents at the C-6 position is tolerable, but not positive. Only compound **15** showed antileishmanial activity with IC_50_ values below 10 µM against the two evolutionary forms of the parasite, confirming the results obtained in Table 2, for its derivative **28**. However, the same analogy was not observed for compound **13**, which had a loss of activity when compared to its derivative **Me-6CN83**.

The substitution of the cyclohexyl ring by a piperidinyl ring (in **23**) also did not promote gains in activity (IC_50_ >10 µM) and also confirmed the results observed in Table 1 for compound **30**.

However, the substitution of the secondary amine of the piperidinyl ring generated ambiguous results. While the presence of the benzyl group, as observed in **18**, did not increase the activity (inactive compound), the presence of the tert-butyloxycarbonyl (Boc) group in **17** resulted in a compound with antileishmanial activity against the promastigote form (IC_50_ = 8.61 µM), therefore being more active than its inactive indole-hybrid derivative **31** (Table 1: IC_50_ >10 µM).

These results suggest that the presence of a free secondary amine in this region, which can act as a hydrogen bond donor group, is not favorable to anti-Leishmania activity, but N-substitution has the potential to generate active compounds, thus opening new perspectives for exploring substitution patterns in this position.

#### 2.2.4. Effect of Substituent at C-3

After confirming that the simplest Gewald adducts possessed antileishmanial activity, we decided to expand the study of pharmacomodulation and evaluate the impact on modifying different functional groups at the C-3 position. To this end, we promoted the synthesis of different Gewald adducts containing ester and amide groups at the C-3 position and evaluated their antileishmanial potential against *L. amazonensis* (Table 3).

As observed in Table 3, and comparing the results with the data of Table 2, it is generally observed that replacing the 3-carbonitrile group (-CN) with 3-carboxamide (-CONH_2_) (as observed for compounds **2**, **7**, and **8**) and 3-carboxylates (COOR) (as observed for compounds **3**, **9**–**11**, and **20**) is not favorable for the antileishmanial activity of Gewald adducts. None of these derivatives showed antileishmanial activity below the established cut-off point (10 µM) and additionally resulted in the complete loss of activity of compound **7CN** (substituted with 3-CN) against the two evolutionary forms of *L. amazonensis*.

The substitution of carbonitrile at C-3 by 3-carboxamide and 3-carboxylate only showed positive pharmacomodulation for anti-Leishmania activity in the 2-aminothiophenes containing the N-Boc-piperidinyl moiety linked to C-4 and C-5 (compounds **19**, **21**, and **22**). The maintenance of anti-promastigote activity (IC_50_ 8.61 versus 9.35 µM) was observed when the 3-carbonitrile in **17** was replaced by 3-carboxamide in **19**.

An increase in anti-promastigote activity and anti-amastigote activity was observed for the 3-carboxylate-substituted derivatives (**21** and **22**) when compared to the 3-carbonitrile-substituted analogue (**17**), showing that this modification was very favorable for the activity.

The activity was more pronounced for compound **21** (IC_50_ = 3.1 and 2.9 µM, for promastigotes and amastigotes, respectively), which was substituted by a 3-ethyl-ester group. The substitution of the radical ethyl by the tert-butyl, as observed in compound **22**, promotes a decrease in activity by more than 2-fold (IC_50_ = 6.52 and 8.35 µM, for promastigotes and amastigotes, respectively), indicating the existence of limits to the size/volume of the alkyl radical of the aliphatic portion.

#### 2.2.5. Effect of S-Se Bioisosterism

The last planned structural modification aimed to evaluate the importance of the presence of sulfur (S) in the pharmacomodulation of antileishmanial activity by performing a classic bioisosteric S/Se, yielding 2-aminoselenophene-indole hybrids.

For this purpose, new 2-aminoselenophene-indole compounds were obtained and had their anti-promastigote potential against *L. amazonensis* evaluated (Table 4) and compared against the respective 2-AT-indole hybrids obtained in our previous studies [22,24].

The data presented in Table 4 indicate that six of the nine 2-aminoselenophenes showed potential to inhibit the promastigote forms of *L. amazonensis* at a concentration lower than 10 μM. Only compounds **32** (indole) and **35** and **40** (5-methyl- and 5-methoxy-indole, respectively) were not active up to the highest concentration evaluated.

The most active 2-AS derivatives were compounds **34** (4-NO_2_), **36** (7-CH_3_), **38** (5-Br), and **39** (4-OCH_3_), which presented IC_50_ values below to 3.5 μM.

These results confirm the already observed importance of the 5-bromoindole radical (as observed in **38**) for anti-Leishmania activity [26], as well as demonstrating that the presence of other electron–donor and electron–withdrawn radicals in different positions of the indole ring is tolerable, as already observed in the study conducted by Félix et al. (2016) [24].

The effect of the S/Se bioisosteric substitution for these 2-aminoselenophenes derivatives seems to be favorable for antileishmanial activity, generally generating compounds with equivalent or superior activity to their respective 2-amino-thiophenes analogues.

Four 2-AS—**33** (IC_50_ = 9.32 μM), **34** (IC_50_ = 2.14 μM), **36** (IC_50_ = 2.92 μM), and **39** (IC_50_ = 2.15 μM)—showed higher activity than their respective 2-AT analogues published in Félix et al. (2016) [24], **TN6-7** (IC_50_ = 14.2 μM), **TN6-4** (IC_50_ = 85.3 μM), **TN6-3** (IC_50_ = 100.8 μM), and **TN6-2** (IC_50_ = 17.9 μM).

Two 2-AS—**32** (IC_50_ > 10.0 μM) and **38** (IC_50_ = 3.49 μM)—have equipotent activity to their respective 2-AT analogues, **SB-83** (IC_50_ = 3.37 μM) and **TN6** (IC_50_ = 14.7 μM). In addition, only compound **35** (IC_50_ > 10.0 μM) showed lower activity than its respective 2-AT, compound **TN6-1** (IC_50_ = 7.2 μM).

Furthermore, and contrary to the prediction of increased cytotoxicity of organoelenium compounds [30,31], it was observed that all analyzed 2-AS presented high selectivity indexes (SIs) ranging between 21.83 and 83.00, being much higher than the SI of the reference drugs AmpB and pentamidine (SI = 1.1 and 6.9, respectively). Therefore, they are drug candidates with a large margin of therapeutic safety.

We also highlight that, although both bioisosters **38** and **SB-83** presented equivalent anti-Leishmania activity, the 2AS derivative **38** (SI = 68) presented twice the safety margin of its bioisoster 2AT **SB-83** (SI = 33.6). This demonstrates that the bioisosteric modification S/Se, having promoted an increase in anti-Leishmania activity, and also contributed to reduce the cytotoxicity.

## 3. Materials and Methods

### 3.1. Chemistry/Synthesis

#### 3.1.1. Synthesis of 4-Unsubstituted 2-Amino-Thiophenes (**1**–**3**)

2,5-dimethyl-2,5-dihydroxy-1,4-dithiane or 1,4-dithiane-2,5-diol (1 eq., 13.0 mmol) and the respective α-activated acetonitriles (malononinitrile, cyanoacetamide or ethyl cyanoacetate) (2 eq.) were added to a round-bottom flask and solubilized in ethanol (15 mL). The reaction flask was placed in an ice bath to drip morpholine (1 eq.). At the end of the base addition, the reaction remained in the ice bath for 1 h and was then heated to 50 °C for a further 1–2 h. The reactions were monitored by thin-layer chromatography (TLC). After cooling, a precipitate was formed and purified by filtration and washing with ethanol. Further purification was performed by recrystallization in ethanol when necessary.

#### 3.1.2. Synthesis of 4,5-Cyclosubstituted-2-Amino-Thiophene-3-Substituted and 2-Amino-Cyclohexyl[b]Selenophene-3-Carbonitrile Compounds (**7CN**, **8CN**, and **4**–**22**)

The respective cyclic ketones (1 eq., 11.0 mmol), the α-activated acetonitriles (1 eq.), and the chalcogen (sulfur or selenium) (1 eq.) were added in a round-bottom flask. Next, ethanol (30 mL) was added and the reactional flask was placed in an ice bath to drip morpholine (1.3 eq.). Then, 30 min after adding the base, the reactions was kept stirring at 50–60 °C. All reactions were monitored by TLC, with their endpoints noted after 3–48 h.

The expected derivatives precipitated for the majority of compounds after cooling and were then purified by filtration and washing with ice-cold ethanol. Further purification was conducted by recrystallization in ethanol or traditional column chromatography in a hexane/ethyl acetate eluent system when necessary.

A dark-colored crude oil was formed for the compounds **8CN** and **6**, after the first purification attempts, leading to produce its solid forms after dissolution in an 1:1 ethanol/water solution and a few days of cooling.

The crude mixture for the selenophenic derivative **12** was cooled to room temperature at the end of the reaction to add activated carbon and kept under stirring for about 15 min in order to adsorb the remaining selenium. At the end of this process, the reaction mixture was vacuum-filtered under celite, providing a dark-colored liquid that was concentrated. The result was a reddish-brown liquid that was subjected to liquid–liquid extraction with dichloromethane and distilled water. The organic phase was concentrated, providing a new viscous liquid, which was solubilized in diethyl ether, and led to forming a solid after cooling. Further purification was conducted by recrystallization in isopropanol, yielding a brownish-red solid.

#### 3.1.3. Deprotection Reaction of 2-Amino-6-N-Boc-Piperidine[b]Thiophene-3-Carbonitrile (**17**)

The deprotection of amine **17** (1 eq., 0.71 mmol) was performed by submitting it to an acidic medium with 7.1 mL of a dichloromethane:trifluoroacetic acid solution (8:2). This mixture was added to an ice bath and left in this condition under stirring for 20 min. The reaction was monitored by TLC, and once it was complete, the solvent was concentrated, yielding a solid. Further purification was performed by recrystallization in ethanol, providing an amorphous crystal of **23**.

#### 3.1.4. Synthesis of 2-Aminothiophene Derivatives Condensed with 5-Bromoindol-3-Carboxaldehyde (**SB-83**, **SB-200**, **8CN83**, **Me-6CN83**, and **25**–**31**)

The synthesized Gewald adducts (**7CN**, **8CN**, **4**–**6**, **13**–**17**, and **23**) (1 eq.) and 5-bromo-indol-3-carboxaldehyde (1 eq.) were added to a round-bottom flask, followed by solubilization in ethanol (5 mL) and dripping of the acetic acid catalyst (10 drops). The reactions were kept stirring at room temperature and monitored by TLC. The expected derivatives precipitated in the reaction medium and were then purified by filtration and washing with ice-cold ethanol.

#### 3.1.5. Synthesis of 2-Aminoselenophene-Indole Hybrids (**32**–**40**)

The Gewald adduct **14** (1 eq., 1.1 mmol) and the respective substituted indolic aldehydes (1 eq.) were added to a round-bottom flask and solubilized in ethanol (5 mL). The acetic acid catalyst was dripped into the reaction medium (10 drops), and the reactions were kept stirring at room temperature and monitored by TLC. Once completed, the obtained precipitates were purified by filtration and washing with cold ethanol.

#### 3.1.6. Structural Identification of the Derivatives Obtained

Compounds **1** [32]; **2** and **3** [33]; **4** [34]; **5** and **7CN** [35]; **8CN** [36]; **7** [37]; **8** and **19** [38]; **9**–**11** [39]; **12** [40]; **13** and **17** [41]; **14** and **16** [42]; **15** [43]; **18** [44]; **20** [45]; **21** and **22** [46]; **23** [47]; **25**, **8CN83**, **26**, **Me-6CN83**, and **30** [26]; **SB83** and **SB200** [22] had their structures confirmed by comparison with NMR spectral data from the respective literature.

All of the other new compounds (**6**, **24**, **27**–**29**, and **31**–**40**) were characterized to confirm their chemical structures, and their physicochemical properties and spectroscopic and spectrometric data are described in the Supplementary Materials.

### 3.2. In Vitro Anti-Leishmania and Cytotoxicity Assays

#### 3.2.1. Parasite and Cell Cultures

The in vitro tests utilized *Leishmania amazonensis* parasites (IFLA/BR/67/PH8). These leishmania cell lines were obtained from Oswaldo Cruz Foundation (FIOCRUZ)/Brazil. The promastigote forms were cultivated in Schneider’s insect medium at 26 °C in a biological oxygen demand (BOD) incubator. Schneider’s insect medium was supplemented with 20% fetal bovine serum (FBS) and 1% penicillin (100 U/mL) and streptomycin (µg/mL) (complete Schneider). Next, we differentiated them from promastigote forms by changing the conditions to Schneider’s insect medium supplemented with 5% FBS, pH 4.6, and a temperature of 32 °C to obtain axenic amastigote forms of *L. amazonensis*. Meanwhile, RAW 264.7 macrophages were cultured in 750 cm^2^ cell culture flasks using DMEM medium supplemented with 10% FBS, 1% penicillin 100 U/mL, and 100 μg/mL streptomycin (complete DMEM) and incubated at 37 °C and 5% CO_2_.

#### 3.2.2. Antileishmanial Activity of Promastigote and Axenic Amastigote Forms of *L. amazonensis*

Parasite growth inhibition was evaluated using the methodology described by Rodrigues et al. (2015) [22]. Cultures of logarithmically growing promastigote and axenic amastigote forms (1 × 10^6^ parasites/mL) were incubated in 96-well plates containing 100 µL of supplemented Schneider’s medium in triplicate. Serial concentrations of 2-amino-thiophene and 2-amino-selenophene derivatives (10.00–0.156 μM) and the reference drugs meglumine antimoniate (500–40,000 μg/mL), amphoterecinB (0.1–2 μM), and pentamidine (3.12–100 µM) were added. BNP was used as a negative control. The cultures were incubated in a biological oxygen demand oven (model Eletrolab EL202, São Paulo, Brazil) at 26 °C for promastigotes and 32 °C for axenic amastigotes for 72 h. Then, 10 μL of MTT (5 mg/mL) was added to each well to colorimetrically evaluate growth inhibition. The plate was subsequently incubated for a further 4 h, followed by adding 50 μL of 10% sodium dodecyl sulfate solution for formazan crystal formation before the optical density of the culture was read on a microplate spectrophotometer reader. The reading was taken at the test and reference wavelengths of 540 and 620 nm using a microplate spectrophotometer. The negative control consisted of 0.2% Schneider’s medium with BNP, considered to be 100% viable.

#### 3.2.3. Determination of Cytotoxicity in Macrophages and Selectivity Index (SI)

First, 100 μL DMEM medium complete with 1 × 10^6^ macrophages (RAW 264.7) was added to each well of a 96-well plate. RAW 264.7 macrophages were obtain from ATCC: code TIB-71^TM^. The plate was placed in an incubator (37 °C, 5% CO_2_) (Sanyo, model COM-15A, Sanyo Electric Co., Osaka, Japan) for 4 h to promote cell adhesion. After this period, two washes were conducted with previously heated DMEM medium (37 °C) to remove non-adhered cells. 2-amino-thiophene and 2-amino-selenophene derivatives were added to wells at serial concentrations of 400 to 25 µM and then incubated for 48 h. Next, 10 μL of MTT at a final concentration of 5 mg/mL were added to each well and incubated for 4 h. The plate was then shaken for 30 min and read at the test and reference wavelengths of 550 and 620 nm, respectively, in a plate reader. DMEM medium supplemented with 0.5% DMSO was used as a negative control and considered as 100% macrophage viability. The selectivity index for each treatment was calculated as the ratio between the concentration of cytotoxicity of RAW 264.7 macrophages (CC_50_) and the 50% inhibitory concentration (IC_50_) or effective concentration (EC_50_), as measured for the different evolutionary forms of *L. amazonensis*.

### 3.3. Chemical Stability Assays

The stability assay was conducted in triplicate at pH 1.2 (Clark-Lubs buffer) and pH 7.4 (commercial phosphate-buffered saline). Solutions of the 2-AT derivatives were prepared to achieve a concentration of 1500 ng/mL using an intermediate acetonitrile solution of 150 µg/mL. The mixture was kept at 37 °C in a water bath with gentle agitation in 2 mL plastic microtubes. After starting the experiment, aliquots of 50 µL were collected and transferred to Max Recovery vials for analysis.

The analysis was performed using an Acquity H-Class UPLC (Waters, Milford, MA, USA) coupled to a Xevo G2-S QT high-resolution mass spectrometer with ESI ionization (Waters, Milford, MA, USA). Data acquisition was performed using the MassLynx 4.1 software. Chromatographic separation utilized an HSS SB C18 column (2.1 × 100 mm, 1.7 µm) at 35 °C with a gradient method over 11 min and a 2 µL injection volume. Mass spectrometry detection parameters were optimized through direct infusion experiments, with key settings including a capillary voltage of 3.5 kV, cone voltage of 40 V, source temperature of 150 °C, and desolvation gas flow of 500 L/h. Data were acquired in the MSE mode, alternating between low-energy (4 V) and high-energy (10–40 V) collision settings for comprehensive ion detection over the 0–11 min run time.

## 4. Conclusions

The present study allowed us to expand the exploration of the pharmacophore related to the antileishmanial activity of 2-aminothiophene derivatives and to identify favorable substitution patterns for the activity, which will provide great support for designing new series of compounds with even more pronounced activities.

The evidence that Gewald adducts (simpler 2-aminothiophenes), synthetic precursors of 2-aminothiophene-indole hybrids with antileishmanial activity, also have biological activity reinforces the hypothesis that 2-aminothiophene-indole hybrids can act as reciprocal prodrugs.

Evaluating the effect of the substituent at C-3 on anti-*Leishmania* activity indicated that replacing the carbonitrile (-CN) by carboxamide (-CONH_2_) and carboxylates (-COOR) is not favorable for anti-*Leishmania* activity in the vast majority of cases. However, this observation does not exclude the possibility of investigating the evaluation of the potential of compounds containing *N*-substituted amides.

It was also possible to highlight the importance of the presence and size of cycloalkyl/piperidinyl chains for evaluating the effect of cycloalkyl substituent attached to C-4/C-5 on anti-*Leishmania* activity, and their role in modulating this activity, with emphasis on cycloocta[*b*]thiophene derivatives (8-membered ring).

With regard to the piperidinyl ring linked at C-4/C-5 (containing nitrogen at C-6), it was observed that the *N*-substitution is essential for maintaining antileishmanial activity, thus opening new perspectives for exploring *N*-substitution patterns. In addition to the *N*-piperidinyl substitution, the presence of 3-carboxamide and 3-carboxylate groups in these compounds also produce a positive pharmacomodulation.

Finally, the exploration of the effects of S-Se bioisosteric substitution also proved to be a very favorable strategy to obtain compounds without elevated cytotoxicity and with equivalent or superior antileishmanial activity when compared to their respective 2-aminothiophenes prototypes. This approach has already shown comparable success in recent studies, including for developing new anti-chagasic drugs [48] and adenosine A2A receptor inhibitors [49]. Moreover, it imparts antioxidant properties and enhances the lipophilicity of the molecules [31].

These punctual chemical modifications performed at different positions of the 2-aminothiophene scaffold indicate a rational direction for the synthesis of new derivatives with more promising activities, in a true Hit-to-Lead (H2L) optimization process.

The main findings indicate that compounds with a more promising profile should take into account: (1) replacement of sulfur by selenium; (2) the presence of carbonitrile at C-3; (3) the presence of large cycloalkyl chains linked to C-4/C-5, such as cycloocta[*b*]thiophene; (4) alternatively, the presence of *N*-substituted piperidinyl chains attached to C-4/C-5, associated with the presence of carboxylates at C-3.

In another direction, we understand the urgent need to conduct drug target deconvolution studies, which allow us to determine the primary target or targets of 2-aminothiophene derivatives.

Far beyond the simple and important identification of the biological target related to anti-Leishmania activity, which would allow the elucidation of the mechanism of action, target deconvolution has the potential to contribute to the better establishment and association of the observed activities with the respective phenotypes, while at the same time allowing the use of structure-based drug design (SBDD) approaches to direct the synthesis [50,51,52].

## Figures and Tables

**Table 1:** Chemical structures, anti-*Leishmania* activity against promastigotes and axenic amastigotes of *L. amazonensis*, and cytotoxicity in macrophages of 5-bromoindole 2-aminothiophene hybrid derivatives (IC_50_ and CC_50_ values in µM).

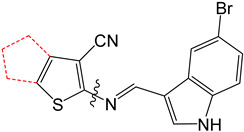				
Compound	Chemical Structures	IC_50_		CC_50_
		Promastigotes	Amastigotes	
**24**	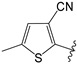	>10.0	>10.0	n.t.
25 *	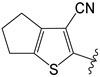	>10.0	>10.0	n.t.
**SB-83** ^#^	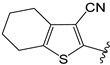	3.37	18.50	113.4
**SB-200** ^#^	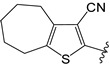	3.65	20.09	32.0
8CN83 *	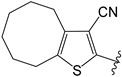	4.75	3.80	>100
26 *	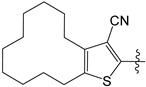	>10.0	>10.0	n.t.
Me-6CN83 *	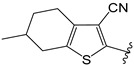	9.90	8.72	>100
**27**	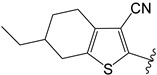	>10.0	>10.0	n.t.
**28**	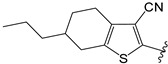	8.12	>10.0	n.t.
**29**	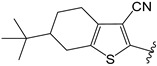	>10.0	>10.0	n.t.
30 *	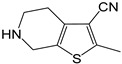	>10.0	>10.0	n.t.
**31**	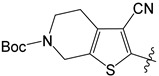	>10.0	>10.0	n.t.
**Meglumine antimoniate**		70.33	2.7	2.7
**Amphotericin B**		0.17	0.23	0.18

* Compounds published in Luna et al. (2023) [26]; ^#^ Compounds published in Rodrigues et al. (2015) [22]; n.t. = not tested.

**Table 2:** Antileishmanial activity of Gewald adducts (2-aminothiophenes) and reference drugs against promastigotes and axenic amastigotes forms of *L. amazonensis* and cytotoxicity in macrophages (IC_50_ and CC_50_ values in µM).

Compound	Chemical Structures	IC_50_		CC_50_
		Promastigotes	Amastigotes	
**1**	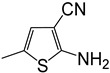	>10.0	>10.0	n.t.
**4**	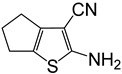	>10.0	>10.0	n.t.
5 *	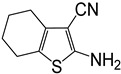	>10.0	n.t.	125.40
7CN *	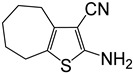	3.14	2.79	99.52
8CN *	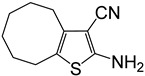	1.20	2.60	43.90
**13**	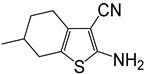	>10.0	>10.0	n.t.
**14**	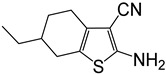	>10.0	>10.0	n.t.
**15**	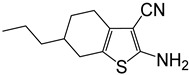	9.2	9.3	n.t.
**16**	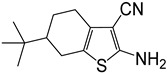	>10.0	>10.0	n.t.
**23**	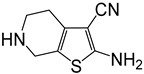	>10.0	>10.0	n.t.
**17**	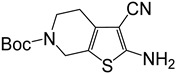	8.61	>10.0	n.t.
**18**	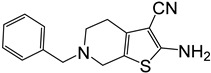	>10.0	>10.0	n.t.
**Meglumine antimoniate**		58.9	2.7	44.9
**Amphotericin B**		0.30	0.23	0.30

n.t. = not tested. * Antileishmanial data of compounds **5**, **7CN**, and **8CN** were previously published [26].

**Table 3:** Antileishmanial evaluation of 3-substituted Gewald adducts and reference drugs against the promastigotes and axenic amastigotes forms of *L. amazonensis* and cytotoxicity in macrophages (IC_50_ and CC_50_ values in µM).

Compounds	Chemical Structures	IC_50_		CC_50_
		Promastigotes	Amastigotes	
**2**	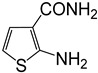	>10.0	>10.0	n.t.
**7**	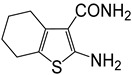	>10.0	>10.0	n.t.
**8**	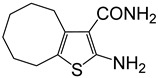	>10.0	>10.0	n.t.
**19**	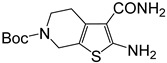	9.35	>10.0	309.7
**3**	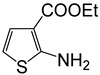	>10.0	>10.0	n.t.
**9**	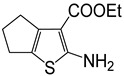	>10.0	n.t.	n.t.
**10**	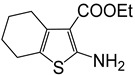	>10.0	n.t.	n.t.
**11**	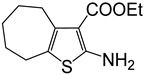	>10.0	n.t.	n.t.
**20**	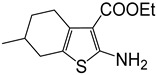	>10.0	n.t.	n.t.
**21**	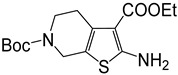	3.1	2.9	220.83
**22**	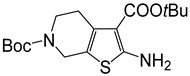	6.52	8.35	194.56
**Meglumine antimoniate**		67.9	2.77	2.77
**Anfotericina B**		0.36	0.23	0.38

n.t.: not tested.

**Table 4:** Anti-promastigote activity of 2-aminoselenophene-indole hybrids and reference drugs against *L. amazonensis* and cytotoxicity in macrophages (IC_50_ and CC_50_ values in µM).

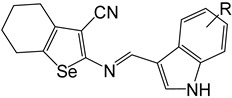				
Compounds	R	Anti-Promastigotes	Cytotoxicity	SI
		IC_50_	CC_50_	
**32**	-H	>10.0	n.t.	-
**33**	5-CN	9.32	203.46	21.83
**34**	4-NO_2_	2.14	189.37	88.49
**35**	5-CH_3_	>10.0	n.t.	-
**36**	7-CH_3_	2.92	185.89	63.66
**37**	4-Br	4.50	241.70	53.71
**38**	5-Br	3.49	238.94	68.45
**39**	4-OCH_3_	2.15	178.46	83.00
**40**	5-OCH_3_	>10.0	n.t.	-
**Amphotericin B**		0.36	0.38	1.10
**Pentamidine**		42.32	293.79	6.90

n.t.: Not tested; SI = CC_50_/IC_50_.

## Data Availability

The original contributions presented in this study are included in the article/Supplementary Materials. Further inquiries can be directed to the corresponding author.

## Supplementary Materials

The following supporting information can be downloaded at: https://www.mdpi.com/article/10.3390/ph18010125/s1. Figure S1: ^1^H NMR full spectra of **6**; Figure S2: ^1^H NMR expanded spectra of **6**; Figure S3: ^1^H NMR expanded spectra of **6**; Figure S4: ^13^C NMR spectra of **6**; Figure S5: Mass spectra of **6**; Figure S6: ^1^H NMR full spectra of **24**; Figure S7: ^1^H NMR expanded spectra of **24**; Figure S8: ^13^C NMR spectra of **24**; Figure S9: Mass spectra of **24**; Figure S10: ^1^H NMR full spectra of **27**; Figure S11: ^13^C NMR spectra of **27**; Figure S12: Mass spectra of **27**; Figure S13: ^1^H NMR full spectra of **28**; Figure S14: ^1^H NMR expanded spectra of **28**; Figure S15: ^1^H NMR expanded spectra of **28**; Figure S16: ^13^C NMR spectra of **28**; Figure S17: Mass spectra of **28**; Figure S18: ^1^H NMR full spectra of **29**; Figure S19: ^13^C NMR spectra of **29**; Figure S20: Mass spectra of **29**; Figure S21: ^1^H NMR full spectra of **31**; Figure S22: ^13^C NMR spectra of **31**; Figure S23: Mass spectra of **31**; Figure S24: ^1^H NMR full spectra of **32**; Figure S25: ^1^H NMR expanded spectra of **32**; Figure S26: ^13^C NMR spectra of **32**; Figure S27: Mass spectra of **32**; Figure S28: ^1^H NMR full spectra of **33**; Figure S29: ^1^H NMR expanded spectra of **33**; Figure S30: ^13^C NMR spectra of **33**; Figure S31: Mass spectra of **33**; Figure S32: ^1^H NMR full spectra of **34**; Figure S33: ^1^H NMR expanded spectra of **34**; Figure S34: ^13^C NMR spectra of **34**; Figure S35: Mass spectra of **34**; Figure S36: ^1^H NMR full spectra of **35**; Figure S37: ^1^H NMR expanded spectra of **35**; Figure S38: ^13^C NMR spectra of **35**; Figure S39: Mass spectra of **35**; Figure S40: ^1^H NMR full spectra of **36**; Figure S41: ^1^H NMR expanded spectra of **36**; Figure S42: ^13^C NMR spectra of **36**; Figure S43: Mass spectra of **36**; Figure S44: ^1^H NMR full spectra of **37**; Figure S45: ^1^H NMR expanded spectra of **37**; Figure S46: ^13^C NMR spectra of **37**; Figure S47: Mass spectra of **37**; Figure S48: ^1^H NMR full spectra of **38**; Figure S49: ^1^H NMR expanded spectra of **38**; Figure S50: ^13^C NMR spectra of **38**; Figure S51: Mass spectra of **38**; Figure S52: ^1^H NMR full spectra of **39**; Figure S53: ^1^H NMR expanded spectra of **39**; Figure S54: ^13^C NMR spectra of **39**; Figure S55: Mass spectra of **39**; Figure S56: ^1^H NMR full spectra of **40**; Figure S57: ^1^H NMR expanded spectra of **40**; Figure S58: ^13^C NMR spectra of **40**; Figure S59: Mass spectra of **40**; Figure S60: Chromatogram of 10 μg/mL of SB-44 before (top) and after (middle and bottom) chemical hydrolysis. Middle and bottom chromatograms are the aminothiophene and indole products, respectively; Figure S61: Spectra of SB-44 products after chemical hydrolysis. A and B show the aminothiophene hydrolysis product at low energy with the parent ion (*m*/*z* 193) in A and its product ions at high energy in B. C and D show the indole hydrolysis product at low energy with the parent ion (*m*/*z* 146) in C and its product ions at high energy in D; Figure S62: Chromatogram of 10 μg/mL of SB-83 before (top) and after (middle and bottom) chemical hydrolysis. Middle and bottom chromatograms are the aminothiophene and indole products, respectively; Figure S63: Spectra of SB-83 products after chemical hydrolysis. A and B show the aminothiophene hydrolysis product at low energy with the parent ion (*m*/*z* 179) in A and its product ions at high energy in B. C, D, and E show the indole hydrolysis product at low energy with the parent ions of the two expected isotopes (*m*/*z* 224 and 226) in C and their respective product ions at high energy in D and E; Figure S64: Chromatogram of 10 μg/mL of SB-200 before (top) and after (middle and bottom) chemical hydrolysis. Middle and bottom chromatograms are the aminothiophene and indole products, respectively; Figure S65: Spectra of SB-200 products after chemical hydrolysis. A and B show the aminothiophene hydrolysis product at low energy with the parent ion (*m*/*z* 193) in A and its product ions at high energy in B, C, D, and E show the indole hydrolysis product at low energy with the parent ions of the two expected isotopes (*m*/*z* 224 and 226) in C and their respective product ions high energy in D and E; Figure S66: Chromatogram of 10 μg/mL of TN8-1 (top) and after (middle and bottom) chemical hydrolysis. Middle and bottom chromatograms are the aminothiophene and indole products, respectively; Figure S67: Spectra of TN8-1 products after chemical hydrolysis. A and B show the indole hydrolysis product at low energy with the parent ion (*m*/*z* 160) in A and its product ions at high energy in B. C and D show the aminothiophene hydrolysis product at low energy with the parent ion (*m*/*z* 207) in C and its product ions at high energy in D; Figure S68: Chromatogram of 10 μg/mL of TN8-2 (top) and after (middle and bottom) chemical hydrolysis. Middle and bottom chromatograms are the aminothiophene and indole products, respectively; Figure S69: Spectra of TN8-2 products after chemical hydrolysis. A and B show the aminothiophene hydrolysis product at low energy with the parent ion (*m*/*z* 207) in A and its product ions at high energy in B. C and D show the indole hydrolysis product at low energy with the parent ion (*m*/*z* 176) in C and its product ions at high energy in D; Table S1: Product from the aminothiophene derivatives using ultraperformance LC coupled with hybrid quadrupole-time of flight after chemical hydrolysis.
