# Supplementary material for: 2-Aminothiophene Derivatives—New Drug Candidates Against Leishmaniasis: Drug Design, Synthesis, Pharmacomodulation, and Antileishmanial Activity
Source: Pharmaceuticals (Basel). 2025 Jan 17;18(1):125. doi: 10.3390/ph18010125 (PMC11768359; doi:10.3390/ph18010125)
Supplement: Supplementary file 1 [file pharmaceuticals-18-00125-s001.zip › pharmaceuticals-3414285-supplementary.pdf]

pharmaceuticals

MDPI

Article

2-Aminothiophene Derivatives—New Drug Candidates Against Leishmaniasis: Drug Design, Synthesis, Pharmacomodulation, and Antileishmanial Activity

Rodrigo Santos Aquino de Araújo [1,2], Vitória Gaspar Bernardo [1,2], Robert da Silva Tibúrcio [3], Danilo Cesar Galindo Bedor [4], Michel Leandro de Campos [5], Roberto Pontarolo [6], Julyanne Maria Saraiva de Sousa [7], Klinger Antonio da Franca Rodrigues [6], Marcus Tullius Scotti [2], Anuraj Nayarisseri [8], Pascal Marchand [9] and Francisco Jaime Bezerra Mendonça-Junior [1,2,*]

1 Laboratory of Synthesis and Drug Delivery, Department of Biological Sciences, State University of Paraíba, João Pessoa 58071-160, Brazil
2 Postgraduate Program in Natural and Synthetic Bioactive Products, Federal University of Paraíba, João Pessoa 58051-900, Brazil
3 Laboratory of Planning and Synthesis in Medicinal Chemistry, Pharmaceutical Sciences Department, Federal University of Pernambuco, Recife 50740-520, Brazil
4 Pharmaceutical and Cosmetic Development Center (NUDFAC), Department of Pharmaceutical Science, Federal University of Pernambuco, Recife 50740-520, Brazil
5 Health Sciences Institute, Federal University of Mato Grosso (UFMT), Sinop 78550-000, Brazil
6 Departamento de Farmácia, Universidade Federal do Paraná, Av. Prefeito Lothário Meissner 632, Curitiba 80210-170, Brazil
7 Infectious Disease Laboratory, Campus Ministro Reis Velloso, Federal University of Parnaíba Delta, Parnaíba 64202-020, Brazil
8 In Silico Research Laboratory, Eminent Biosciences, Mahalakshmi Nagar, Indore 452010, India
9 Cibles et Médicaments des Infections et de l'immunité, IICiMed, Nantes Université, UR 1155, F-44000 Nantes, France
* Correspondence: franciscojaime@servidor.uepb.edu.br

Supplementary Material

Structural characterization of the new 2-aminothiophene and 2-aminoselenophene derivatives

For the new derivatives, their physicochemical properties and spectral data are shown below.

*2-amino-cyclododecacyl[b]thiophen-3-carbonitrile (**6**)*

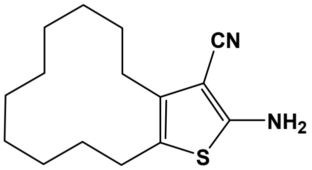

Molecular Formula: $C_{15}H_{22}N_2S$. **Molecular Mass:** 262.4136 g/mol. **R*f*:** 0.68 (7:3 – Hexane/Ethyl acetate). **Appearance:** Black solid. **Melting Point:** 107.6 – 109.5 °C. **Yield:** 33%. **^{1}H NMR** (500 MHz, DMSO-*d6*): δ 6.90 (sl, 2H), 2.54-2.51 (t, *J* = 5.0, 10.0 Hz, 2H), 2.42-2.39 (t, *J* = 5.0, 10.0 Hz, 2H), 1.66-1.62 (qt, 2H), 1.57-1.52 (qt, 2H), 1.40 (m, 4H), 1.32 (m, 4H), 1.29-1.21 (m, 4H). **^{13}C NMR** (125 MHz, DMSO-*d6*): δ 163.46, 133.78, 121.73, 117.18, 84.47, 29.51, 27.08, 24.92, 24.88, 24.49, 24.45, 24.14, 22.52, 22.18. **LC/MS (ESI):** Calculated to $[M+H]^+$: 263.4136 g/mol; Found to $[M+H]^+$: 263.1571 g/mol.

*5-methyl-2-((5′-bromo-indolyl)imino)-thiophen-3-carbonitrile (**24**)*

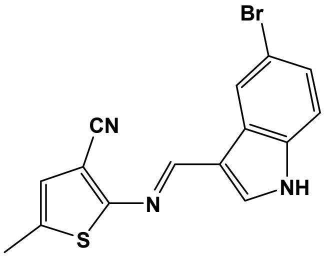

Molecular Formula: $C_{15}H_{10}BrN_3S$. **Molecular Mass:** 342.9779 g/mol. **R*f*:** 0.173 (1:1 – Hexane/Ethyl acetate. **Appearance:** Yellow solid. **Melting Point:** 215.5 – 216.3 °C. **Yield:** 41.6%. **^{1}H NMR** (400 MHz, DMSO-*d6*): δ 12.26 (sl, 1H), 8.81 (s, 1H), 8.59-8.58 (d, 1H, *J* = 4.0 Hz), 8.26-8.25 (d, 1H, *J* = 4.0 Hz), 7.52-7.50 (dd, 1H, *J* = 8.0 Hz), 7.43-7.41 (dd, 1H, *J* = 8.0 Hz), 7.03 (d, 1H), 2.28 (d, 3H). **^{13}C NMR** (100 MHz, DMSO-*d6*): δ 166.03, 156.70, 138.21, 136.66, 136.62, 126.78, 126.58, 124.83, 116.02, 115.50, 115.04, 114.98, 114.16, 105.14, 15.27. **LC/MS (ESI):** Calculated to $[M+H]^+$: 343.9779 g/mol; Found to $[M+H]^+$: 343.9857 g/mol; Found to $[M+2+H]^+$: 345.9832 g/mol.

*(2-(5′-bromo-indolyl)imino)-6-ethyl-cyclohexyl[b]thiophen-3-carbonitrile (**27**)*

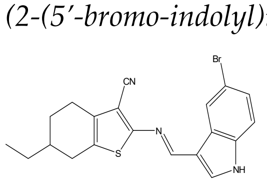

Molecular Formula: $C_{20}H_{18}N_3BrS$. **Molecular Mass:** 411.0405 g/mol. **R*f*:** 0.32 (7:3 – Hexane/Ethyl Acetate). **Appearance:** Yellow solid. **Melting Point:** 265.0 – 268.0 °C. **Yield:** 85.9%. **^{1}H NMR** (400 MHz, DMSO-*d6*): δ 12.22 (sl, 1H), 8.69 (s, 1H), 8.58-8.57 (d, 1H, *J* = 4.0 Hz), 8.22 (s, 1H), 7.51-7.49 (d,

1H, J = 8.0 Hz), 7.42-7,39 (dd, 1H, J = 8.0, 4.0 Hz), 2.79-2.74 (dd, 1H, J = 4.0, 4.0 Hz), 2.62 – 2.53 (m, 1H), 2.52 – 2.50 (m, 1H), 2.30 – 2.28 (m, 1H), 1.93 – 1.90 (m, 1H), 1.68 (m, 1H), 1.40 – 1.34 (m, 3H), 0.94-0.90 (t, 3H, J = 8.0 Hz). **^{13}C NMR** (100 MHz, DMSO-*d6*): δ 163.03, 155.43, 137.76, 136.64, 133.92, 133.90, 130.04, 126.78, 126.77, 126.52, 124.87, 115.30, 114.96, 114.90, 114.25, 103.48, 36.22, 30.95, 28.53, 28.04, 24.13, 11.73, 11.70. **LC/MS (ESI):** Calculated to $[M+H]^+$: 412.0405 g/mol; Found to $[M+2+H]^+$: 414.0447 g/mol.

*(2-(5′-bromo-indolyl)imino)-6-propyl-cyclohexyl[b]thiophen-3-carbonitrile (**28**)*

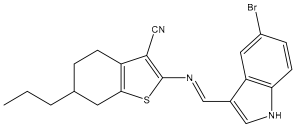

Molecular Formula: $C_{21}H_{20}N_3BrS$. **Molecular Mass:** 425.06 g/mol. **R*f*:** 0.40 (7:3 – Hexane/Ethyl Acetate). **Appearance:** Yellow solid. **Melting Point:** 281.0 – 285.0 °C. **Yield:** 53.2%. **^{1}H NMR** (400 MHz, DMSO-*d6*): δ 12.22 (sl, 1H), 8.69 (s, 1H), 8.58–8.57 (dd, 1H, J = 4.0 Hz), 8.22 (s, 1H), 7.51-7.48 (dd, 1H, J = 8.0, 4.0 Hz), 7.42-7.39 (dd, 1H, J = 8.0, 4.0 Hz), 2.78–2.73 (dd, 1H, J = 12.0, 4.0 Hz), 2.66–2.61 (m, 1H), 2.57–2.53 (m, 1H), 2.30–2.23 (dd, 1H, J = 12.0, 4.0 Hz), 1.92–1.88 (m, 1H), 1.79-1.73 (m, 1H), 1.44-1.27 (m, 5H), 0.90–0.87 (m, 3H, J = 8.0, 4.0 Hz). **^{13}C NMR** (100 MHz, DMSO-*d6*): δ 163.02, 155.43, 137.76, 136.64, 133.91, 130.03, 126.78, 126.52, 124.88, 115.30, 114.97, 114.89, 114.25, 103.49, 38.07, 34.21, 31.26, 28.37, 24.11, 19.92, 14.61. **LC/MS (ESI):** Calculated to $[M+H]^+$: 426.06 g/mol; Found to $[M+H]^+$: 426.0633 g/mol; and Found to $[M+2+H]^+$: 428.0616 g/mol.

*(2-(5′-bromo-indolyl)imino)-6-tert-butyl-cyclohexyl[b]thiophen-3-carbonitrile (**29**)*

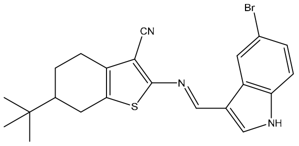

Molecular Formula: $C_{22}H_{22}N_3BrS$. **Molecular Mass:** 439.0718 g/mol. **R*f*:** 0.27 (1:1 – Hexane/Ethyl Acetate). **Appearance:** Yellow solid. **Melting Point:** 280.8 – 282.2 °C. **Yield:** 84.4%. **^{1}H NMR** (400 MHz, DMSO-*d6*): δ 12.23 (sl, 1H), 8.69 (s, 1H), 8.58-8.57 (d, 1H, J = 4.0 Hz), 8.23 (s, 1H), 7.51-7.48 (d, 1H, J = 12.0 Hz), 7.42-7.39 (dd, 1H, J = 8.0, 4.0 Hz), 2.73–2.68 (m, 2H), 2.48–2.42 (m, 2H), 2.02–1.99 (m, 1H), 1.51–1.48 (m, 1H), 1.32–1.28 (m, 1H), 0.91 (s, 9H). **^{13}C NMR** (100 MHz, DMSO-*d6*): δ 163.10, 155.40, 137.80, 136.66, 136.64, 133.94, 130.89, 126.78, 126.52, 124.88, 115.36, 114.97, 114.96, 114.90, 114.25, 103.32, 45.03, 32.77, 27.55, 26.72, 25.26, 23.76. **LC/MS (ESI):** Calculated to $[M+H]^+$: 440.0718 g/mol; Found to $[M+2+H]^+$: 442.0759 g/mol.

*(2-(5′-bromo-indolyl)imino)-6-N-Boc-piperidine[b]thiophen-3-carbonitrile (**31**)*

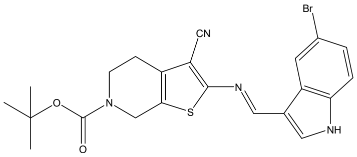

Molecular Formula: $C_{22}H_{21}O_2N_4BrS$. **Molecular Mass:** 484.0569 g/mol. **R*f*:** 0.34 (1:1 – Hexane/Ethyl Acetate). **Appearance:** Yellow solid. **Melting Point:** 245.0 – 247.0 °C. **Yield:** 73.4%. **^{1}H NMR** (400 MHz, DMSO-*d6*): δ 12.25 (sl, 1H), 8.71 (s, 1H), 8.55-8.54 (d, 1H, *J* = 4.0 Hz), 8.24 (s, 1H), 7.49-7.47 (d, 1H, *J* = 8.0 Hz), 7.40-7.38 (dd, 1H, *J* = 8.0, 4.0 Hz), 4.49 (s, 2H), 3.65-3.63 (t, 2H, *J* = 4.0 Hz), 2.65–2.63 (m, 2H), 1.42 (s, 9H). **^{13}C NMR** (100 MHz, DMSO-*d6*): δ 164.04, 156.16, 138.24, 136.64, 132.77, 126.74, 126.57, 124.80, 115.05, 114.94, 114.88, 114.16, 103.05, 80.02, 28.48. **LC/MS (ESI):** Calculated to $[M+H]^+$: 485.0569 g/mol; Found to $[M+H]^+$: 485.0640 g/mol.

*2-((indolyl)imino)-cyclohexyl[b]selenophen-3-carbonitrile (**32**)*

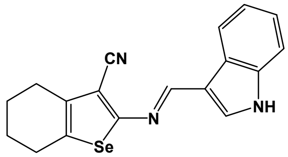

Molecular Formula: $C_{18}H_{15}N_3Se$. **Molecular Mass:** 353.04 g/mol. **R*f*:** 0.71 (1:1 – Hexane/Ethyl acetate). **Appearance:** Golden yellow solid. **Melting Point:** 196 °C. **Yield:** 44%. **^{1}H NMR** (400 MHz, DMSO-*d6*): δ 12.12 (sl, 1H), 8.54 (s, 1H), 8.40-8.39 (d, 1H, *J* = 4.0 Hz), 8.20 (d, 1H, *J* = 1.36 Hz), 7.53-7.52 (d, 1H, *J* = 4.0 Hz), 7.30-7.27 (t, 1H, *J* = 8.0, 4.0 Hz), 7.27-7.24 (t, 1H, *J* = 8.0, 4.0 Hz), 2.76 (m, 2H), 2.55 (m, 2H), 1.80 (m, 4H). **^{13}C NMR** (100 MHz, DMSO-*d6*): δ 170.43, 156.76, 137.98, 137.35, 134.89, 134.33, 125.08, 124.08, 122.56, 122.33, 116.40, 114.83, 112.93, 106.46, 27.53, 25.77, 23.60, 22.17. **LC/MS (ESI):** Calculated to $[M+Na]^+$: 376.03 g/mol; Found to $[M+Na]^+$: 376.02 g/mol.

*2-((5′-cyano-indolyl)imino)-cyclohexyl[b]selenophen-3-carbonitrile (**33**)*

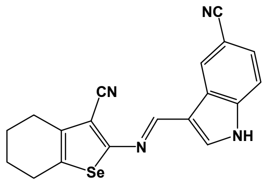

Molecular Formula: $C_{19}H_{14}N_4Se$. **Molecular Mass:** 378.04 g/mol. **R*f*:** 0.42 (1:1 – Hexane/Ethyl acetate). **Appearance:** Yellow solid. **Melting Point:** 178 °C. **Yield:** 57%. **^{1}H NMR** (400 MHz, DMSO-*d6*): δ 12.13 (sl, 1H), 8.78 (s, 1H), 8.60 (s, 1H), 8.34 (s, 1H), 7.72-7.70 (d, *J* = 8.0 Hz), 7.66-7.65 (d, 1H, *J* = 4.0 Hz), 2.76 (m, 2H), 2.55 (m, 2H), 1.80 (m, 4H). **^{13}C NMR** (100 MHz, DMSO-*d6*): δ 169.34, 156.05, 139.81, 138.72, 136.27, 134.45, 127.47, 126.87, 124.95, 120.61, 116.24, 114.96, 114.45, 107.75, 104.41, 27.53, 25.73, 23.53, 22.09. **LC/MS (ESI):** Calculated to $[M+Na]^+$: 401.03 g/mol; Found to $[M+Na]^+$: 401.02 g/mol.

*2-((4′-nitro-indolyl)imino)-cyclohexyl[b]selenophen-3-carbonitrile (**34**)*

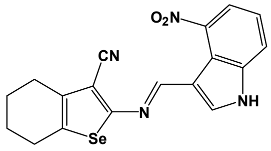

Molecular Formula: $C_{18}H_{14}N_4O_2Se$. **Molecular Mass:** 398.03 g/mol. **R*f*:** 0.74 (1:1 – Hexane/Ethyl acetate). **Appearance:** Reddish-orange solid. **Melting Point:** 280 °C. **Yield:** 45%. **^{1}H NMR** (400 MHz,

DMSO-*d6*): δ 12.92 (sl, 1H), 8.83 (s, 1H), 8.56 (s, 1H), 8.01-7.99 (dd, 1H, *J* = 8.24, 0.32 Hz), 7.97-7.96 (dd, 1H, *J* = 5.32, 0.40 Hz), 7.44-7.41 (t, 1H, *J* = 5.32 Hz), 2.52-2.50 (m, 2H), 2.55 (m, 2H), 1.79 (m, 4H). **^{13}C NMR** (100 MHz, DMSO-*d6*): δ 169.46, 158.02, 142.59, 140.09, 135.97, 135.90, 134.79, 122.57, 120.16, 119.75, 117.70, 115.95, 112.74, 106.94, 27.47, 25.79, 23.51, 22.11. **LC/MS (ESI):** Calculated to $[M+Na]^+$: 421.02 g/mol; Found to $[M+Na]^+$: 421.01 g/mol.

*2-((5′-methyl-indolyl)imino)-cyclohexyl[b]selenophen-3-carbonitrile (**35**)*

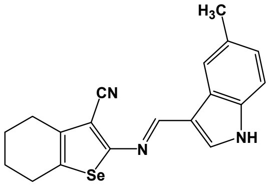

Molecular Formula: $C_{19}H_{17}N_3Se$. **Molecular Mass:** 367.06 g/mol. **R*f*:** 0.71 (1:1 – Hexane/Ethyl acetate). **Appearance:** Reddish-orange solid. **Melting Point:** 210 °C. **Yield:** 53%. **^{1}H NMR** (400 MHz, DMSO-*d6*): δ 12.01 (sl, 1H), 8.50 (s, 1H), 8.25 (s, 1H), 8.13 (s, 1H), 7.40-7.39 (d, 1H, *J* = 5.48 Hz), 7.11-7.10 (d, 1H, *J* = 5.48 Hz), 2.76 (m, 2H), 2.55-2.50 (m, 2H), 2.43 (s, 3H), 1.79 (m, 4H). **^{13}C NMR** (100 MHz, DMSO-*d6*): δ 170.40, 156.53, 137.12, 136.26, 134.70, 134.24, 131.14, 125.41, 122.61, 116.44, 114.52, 112.51, 106.41, 27.54, 25.78, 23.61, 22.17, 21.89. **LC/MS (ESI):** Calculated to $[M+Na]^+$: 390.05 g/mol; Found to $[M+Na]^+$: 390.04 g/mol.

*2-((7′-methyl-indolyl)imino)-cyclohexyl[b]selenophen-3-carbonitrile (**36**)*

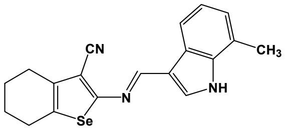

Molecular Formula: $C_{19}H_{17}N_3Se$. **Molecular Mass:** 367.06 g/mol. **R*f*:** 0.80 (1:1 – Hexane/Ethyl acetate). **Appearance:** Yellow-green solid. **Melting point:** 250 °C. **Yield:** 37%. **^{1}H NMR** (400 MHz, DMSO-*d6*): δ 12.14 (sl, 1H), 8.53 (s, 1H), 8.24-8.22 (d, 1H, *J* = 5.2 Hz), 8.20 (s, 1H), 7.17-7.14 (t, 1H, *J* = 5.08, 4.92 Hz), 7.09-7.08 (d, 1H, *J* = 4.76 Hz), 2.74 (m, 2H), 2.54 (m, 2H), 2.51 (s, 3H), 1.79 (m, 4H). **^{13}C NMR** (100 MHz, DMSO-*d6*): δ 170.48, 156.76, 137.44, 137.10, 134.81, 134.32, 124.88, 124.71, 122.51, 122.17, 120.15, 116.41, 115.22, 106.39, 27.53, 25.76, 23.59, 22.16, 17.09. **LC/MS (ESI):** Calculated to $[M+Na]^+$: 390.05 g/mol; Found to $[M+Na]^+$: 390.04 g/mol.

*2-((4′-bromo-indolyl)imino)-cyclohexyl[b]selenophen-3-carbonitrile (**37**)*

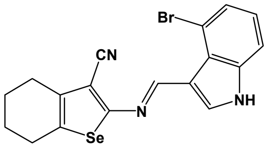

Molecular Formula: $C_{18}H_{14}BrN_3Se$. **Molecular Mass:** 430.95 g/mol. **R*f*:** 0.85 (1:1 – Hexane/Ethyl acetate). **Appearance:** Reddish-orange solid. **Melting Point:** 205 °C. **Yield:** 41%. **^{1}H NMR** (400 MHz, DMSO-*d6*): δ 12.62 (sl, 1H), 9.28 (s, 1H), 8.42 (s, 1H), 7.60-7.58 (d, 1H, *J* = 5.36 Hz), 7.46-7.45 (d, 1H, *J* = 5.00 Hz), 7.19-7.16 (t, 1H, *J* = 5.24, 5.2 Hz), 2.74 (m, 2H), 2.53 (m, 2H), 1.79 (m, 4H). **^{13}C NMR** (100

MHz, DMSO-*d6*): δ 170.04, 155.54, 138.86, 135.43, 134.87, 132.45, 126.39, 124.81, 124.27, 116.16, 113.77, 113.29, 112.84, 106.36, 27.49, 25.80, 23.51, 22.10. **LC/MS (ESI):** Calculated to $[M+Na]^+$: 453.94 g/mol; Found to $[M+Na]^+$: 453.93 g/mol.

*2-((5′-bromo-indolyl)imino)-cyclohexyl[b]selenophen-3-carbonitrile (**38**)*

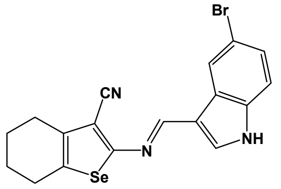

Molecular Formula: $C_{18}H_{14}BrN_3Se$. **Molecular Mass:** 430.95 g/mol. **R*f*:** 0.65 (1:1 – Hexane/Ethyl acetate). **Appearance:** Light brown solid. **Melting Point:** 141 °C. **Yield:** 54%. **^{1}H NMR** (400 MHz, DMSO-*d6*): δ 12.25 (sl, 1H), 8.58 (s, 1H), 8.55 (s, 1H), 8.23 (s, 1H), 7.50-7.49 (d, 1H, *J* = 5.68 Hz), 7.42-7.40 (d, 1H, *J* = 5.68 Hz), 2.76 (m, 2H), 2.56 (m, 2H), 1.80 (m, 4H). **^{13}C NMR** (100 MHz, DMSO-*d6*): δ 169.82, 156.37, 137.96, 136.65, 135.49, 134.40, 126.83, 126.58, 124.91, 116.25, 115.04, 114.94, 114.23, 107.09, 27.54, 25.77, 23.57, 22.14. **LC/MS (ESI):** Calculated to $[M+Na]^+$: 453.94 g/mol; Found to $[M+Na]^+$: 453.94 g/mol.

*2-((4′-methoxy-indolyl)imino)cyclohexyl[b]selenophen-3-carbonitrile (**39**)*

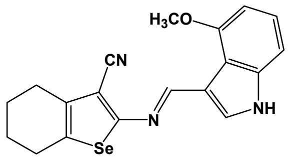

Molecular Formula: $C_{19}H_{17}N_3OSe$. **Molecular Mass:** 383.05 g/mol. **R*f*:** 0.74 (1:1 – Hexane/Ethyl acetate). **Appearance:** Reddish-brown solid. **Melting Point:** 154 °C. **Yield:** 47%. **^{1}H NMR** (400 MHz, DMSO-*d6*): δ 12.29 (sl, 1H), 8.87 (s, 1H), 8.18 (s, 1H), 7.18-7.15 (t, 1H, *J* = 5.32, 5.12 Hz), 7.13-7.12 (d, 1H, *J* = 5.36 Hz), 6.76-6.75 (d, 1H, *J* = 5.08 Hz), 3.96 (s, 3H), 2.75 (m, 2H), 2.53 (m, 2H), 1.80 (m, 4H). **^{13}C NMR** (100 MHz, DMSO-*d6*): δ 170.28, 157.53, 154.36, 138.55, 134.69, 129.39, 124.16, 116.30, 116.20, 113.92, 106.56, 105.73, 102.83, 55.95, 27.48, 25.81, 23.55, 22.14. **LC/MS (ESI):** Claculated to $[M+Na]^+$: 406.04 g/mol; Found to $[M+Na]^+$: 406.04 g/mol.

*2-((5′-methoxy-indolyl)imino)-cyclohexyl[b]selenophen-3-carbonitrile (**40**)*

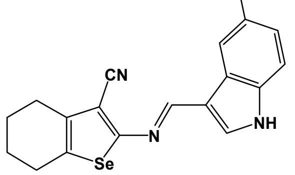

Molecular Formula: $C_{19}H_{17}N_3OSe$. **Molecular Mass:** 383.05 g/mol. **R*f*:** 0.71 (1:1 – Hexane/Ethyl acetate). **Appearance:** Reddish-brown solid. **Melting Point:** 181 °C. **Yield:** 39%. **^{1}H NMR** (400 MHz, DMSO-*d6*): δ 11.99 (sl, 1H), 8.53 (s, 1H), 8.12 (s, 1H), 8.04 (s, 1H), 7.41-7.40 (d, 1H, *J* = 5.84 Hz), 6.90-6.89 (d, 1H, *J* = 5.76 Hz), 3.81 (s, 3H), 2.76 (m, 2H), 2.55 (m, 2H), 1.80 (m, 4H). **^{13}C NMR** (100 MHz, DMSO-*d6*): δ 170.18, 156.34, 155.98, 137.00, 134.75, 134.11, 132.57, 125.98, 116.52, 114.70, 113.94,

113.62, 106.64, 104.51, 55.69, 27.56, 25.74, 23.62, 22.17. **LC/MS (ESI):** Calculated to $[M+Na]^+$: 406.04 g/mol; Found to $[M+Na]^+$: 406.04 g/mol.

Figure S1: ^{1}H NMR full spectra of 6

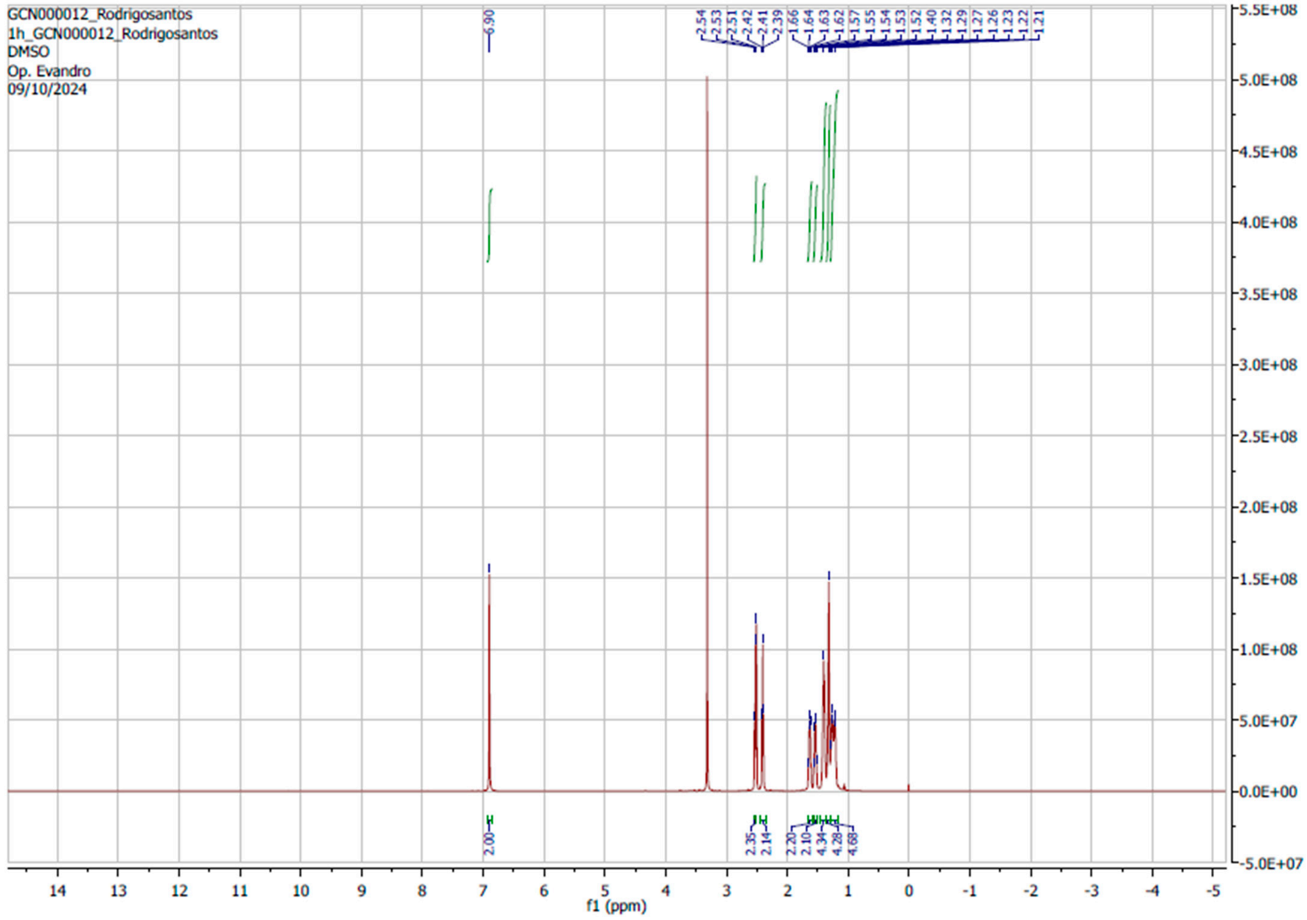

Figure S2: ^{1}H NMR expanded spectra of 6

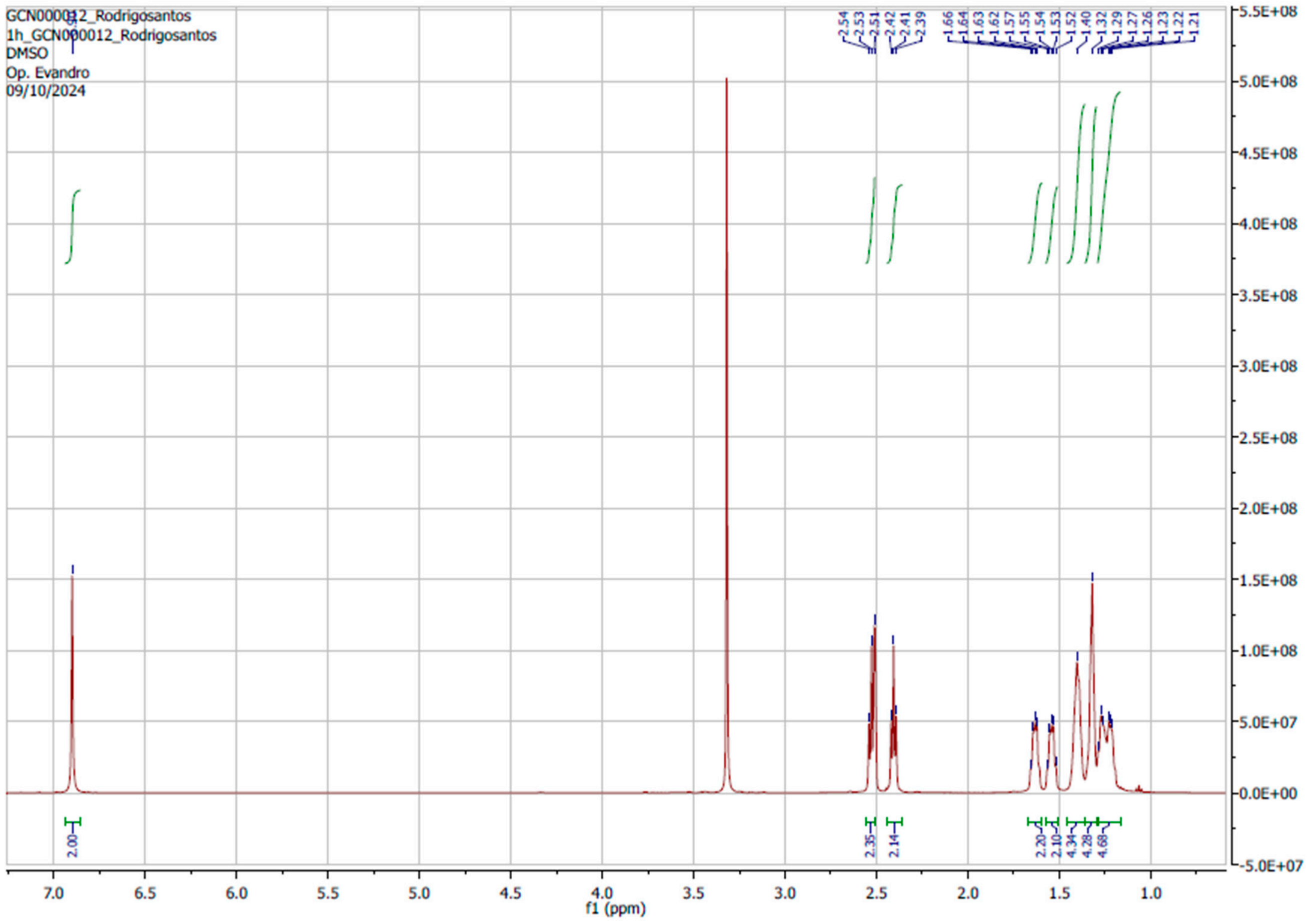

Figure S3: ^{1}H NMR expanded spectra of 6

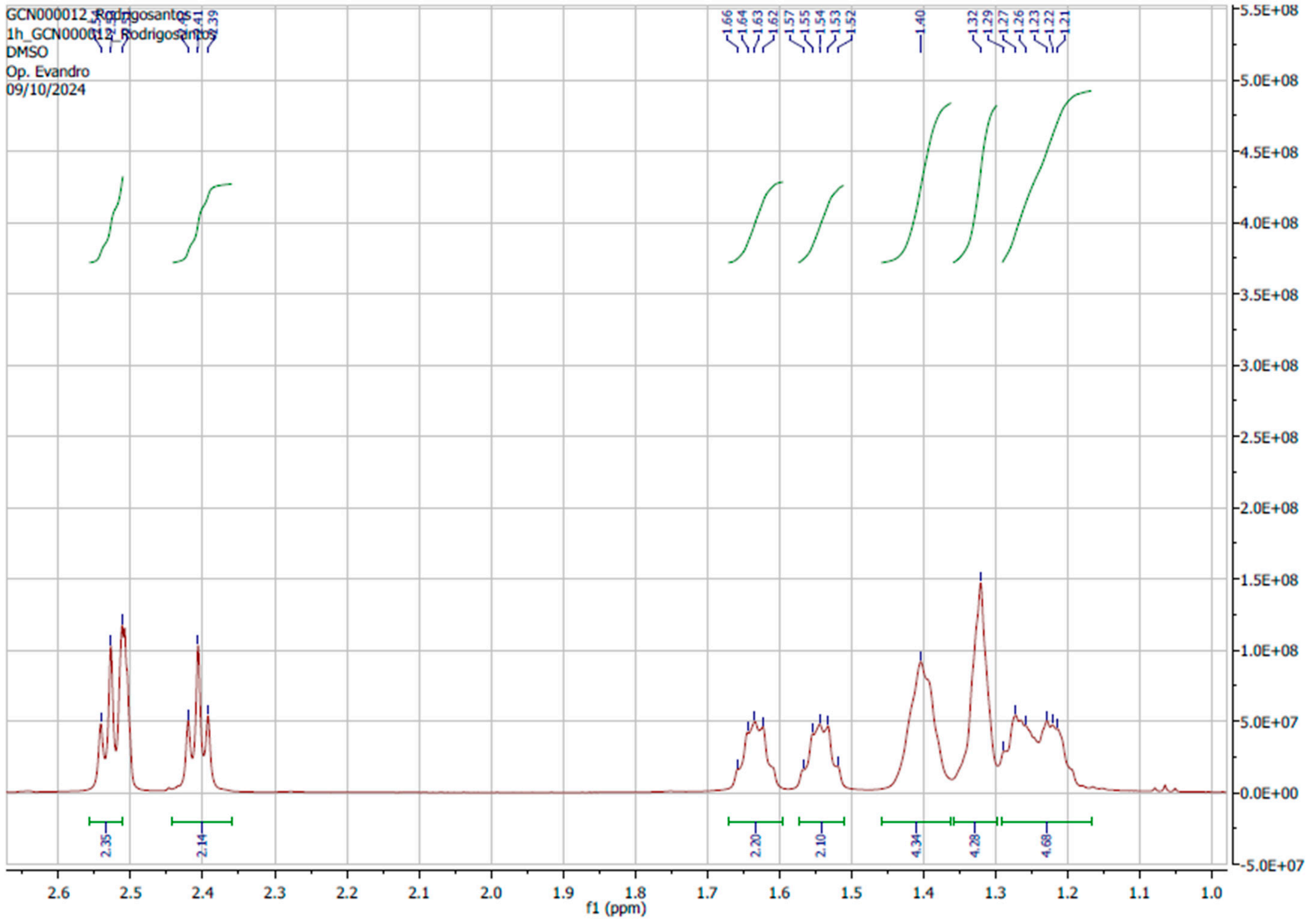

Figure S4: ^{13}C NMR spectra of 6

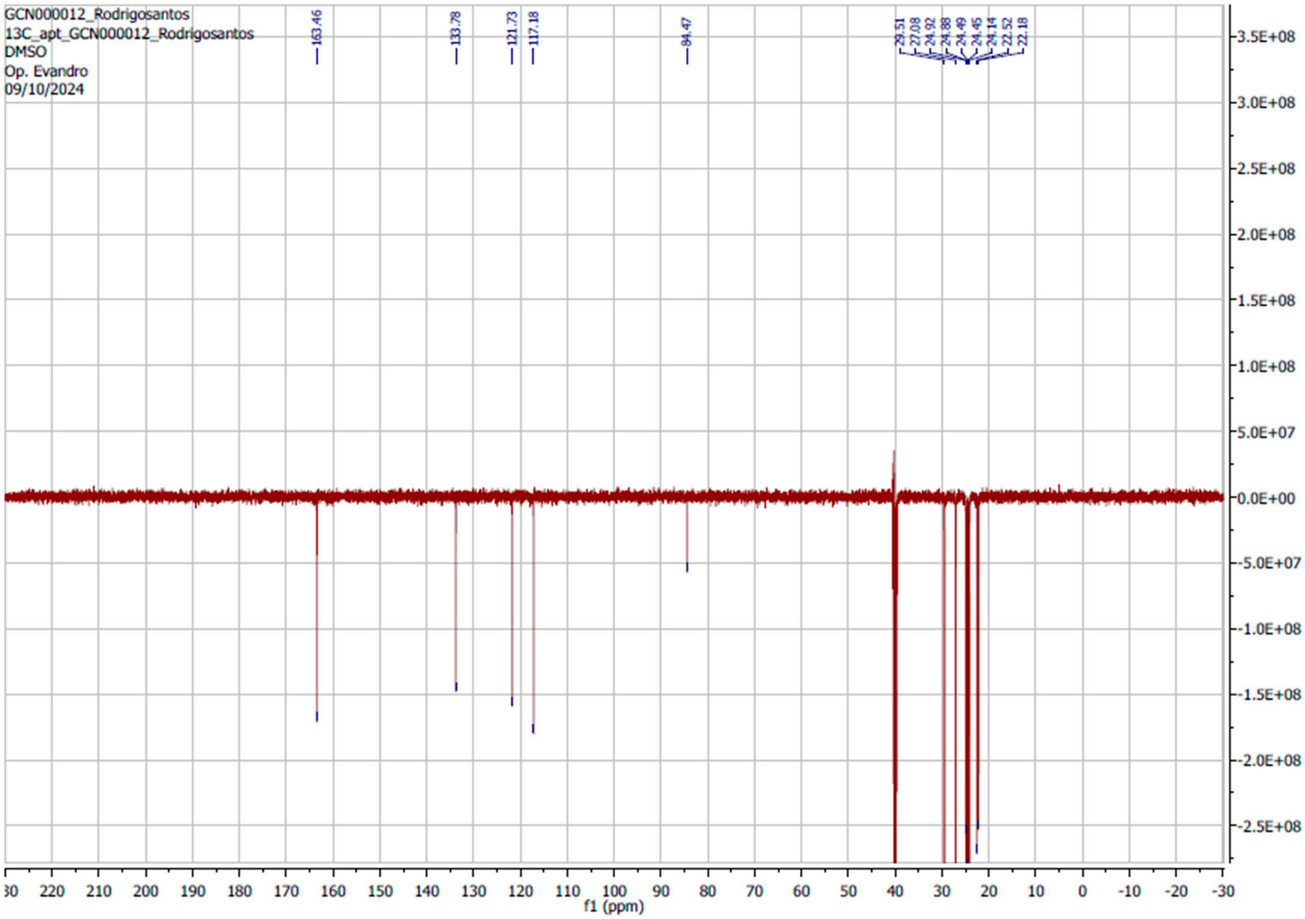

Figure S5: Mass spectra of 6

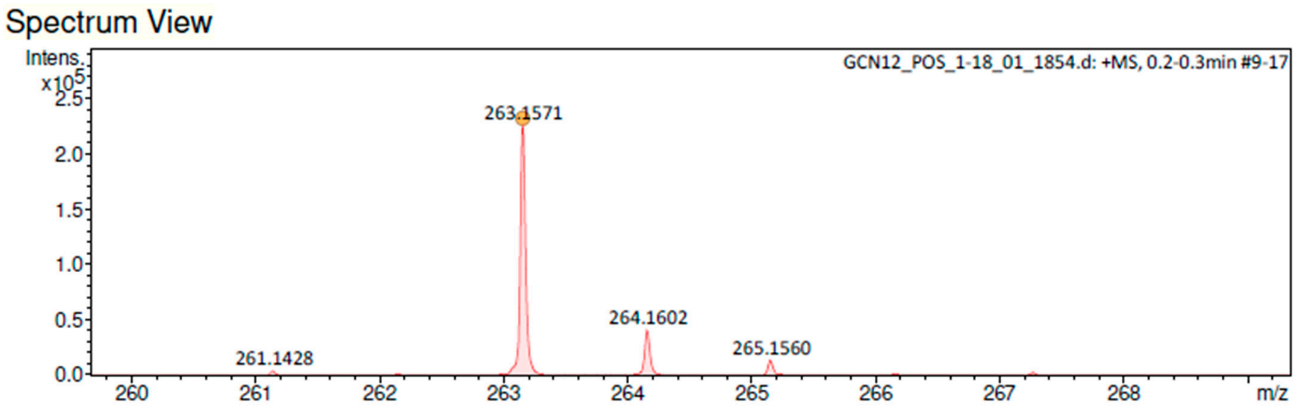

Figure S6: ^{1}H NMR full spectra of 24

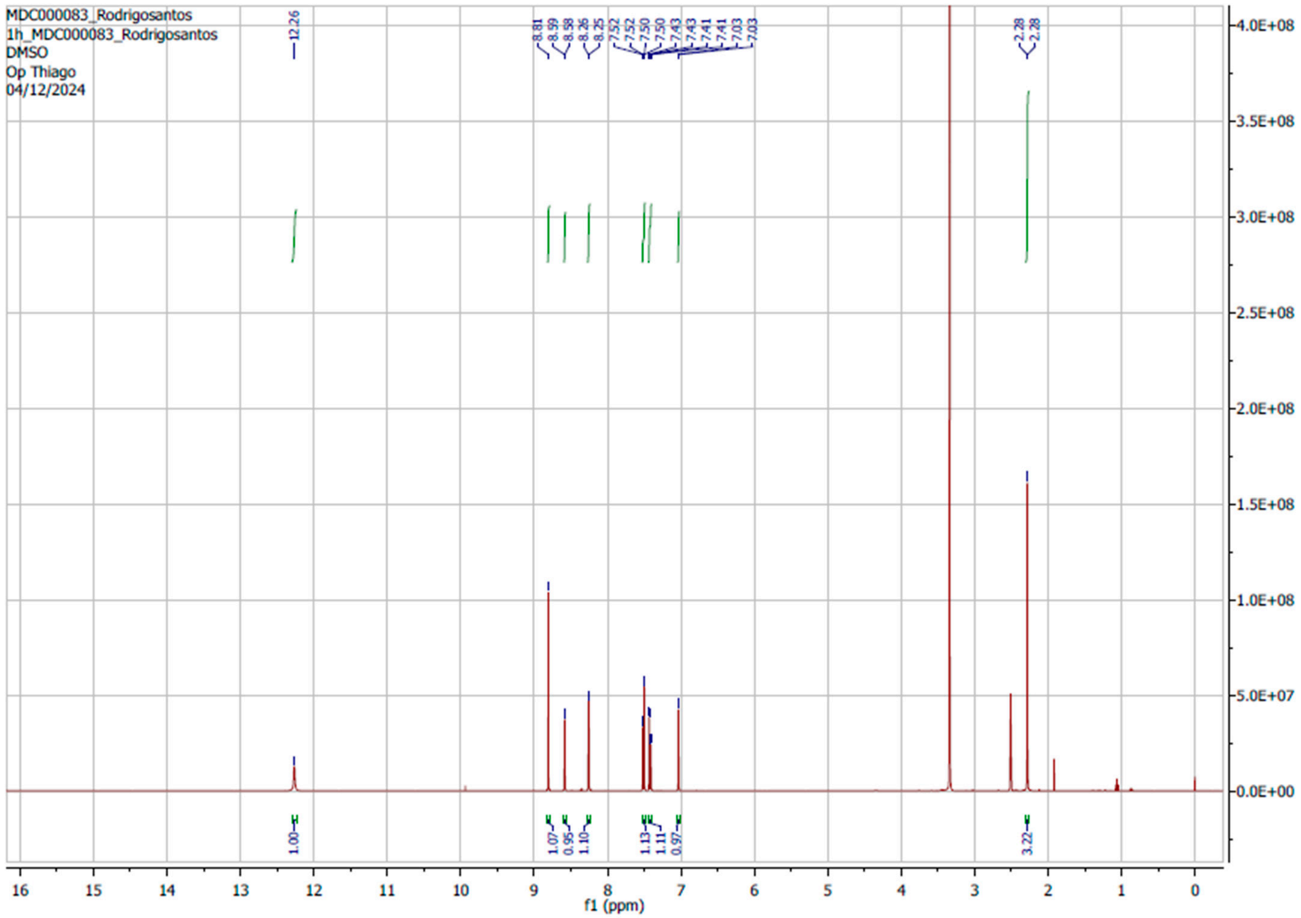

Figure S7: ^{1}H NMR expanded spectra of 24

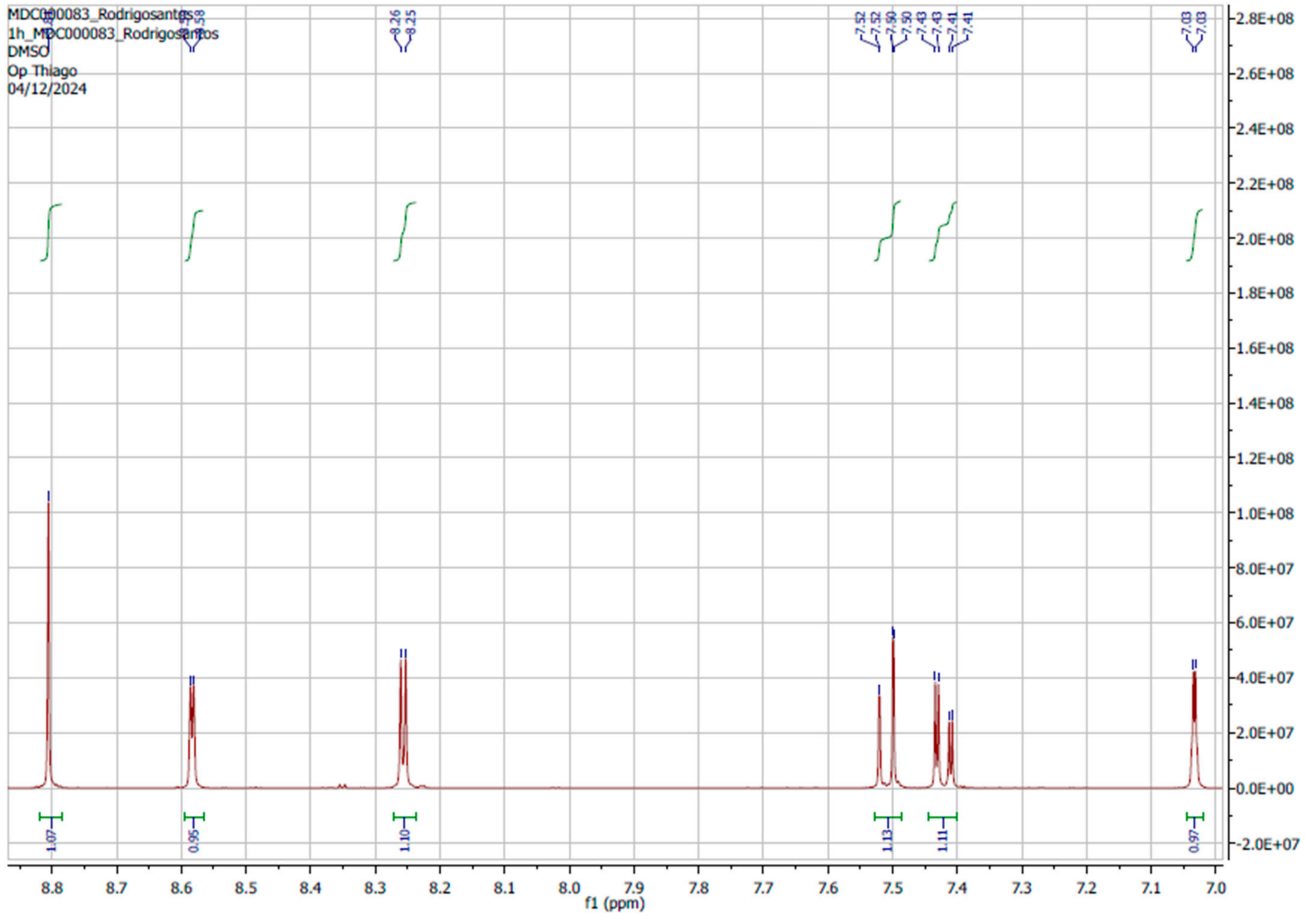

Figure S8: ^{13}C NMR spectra of 24

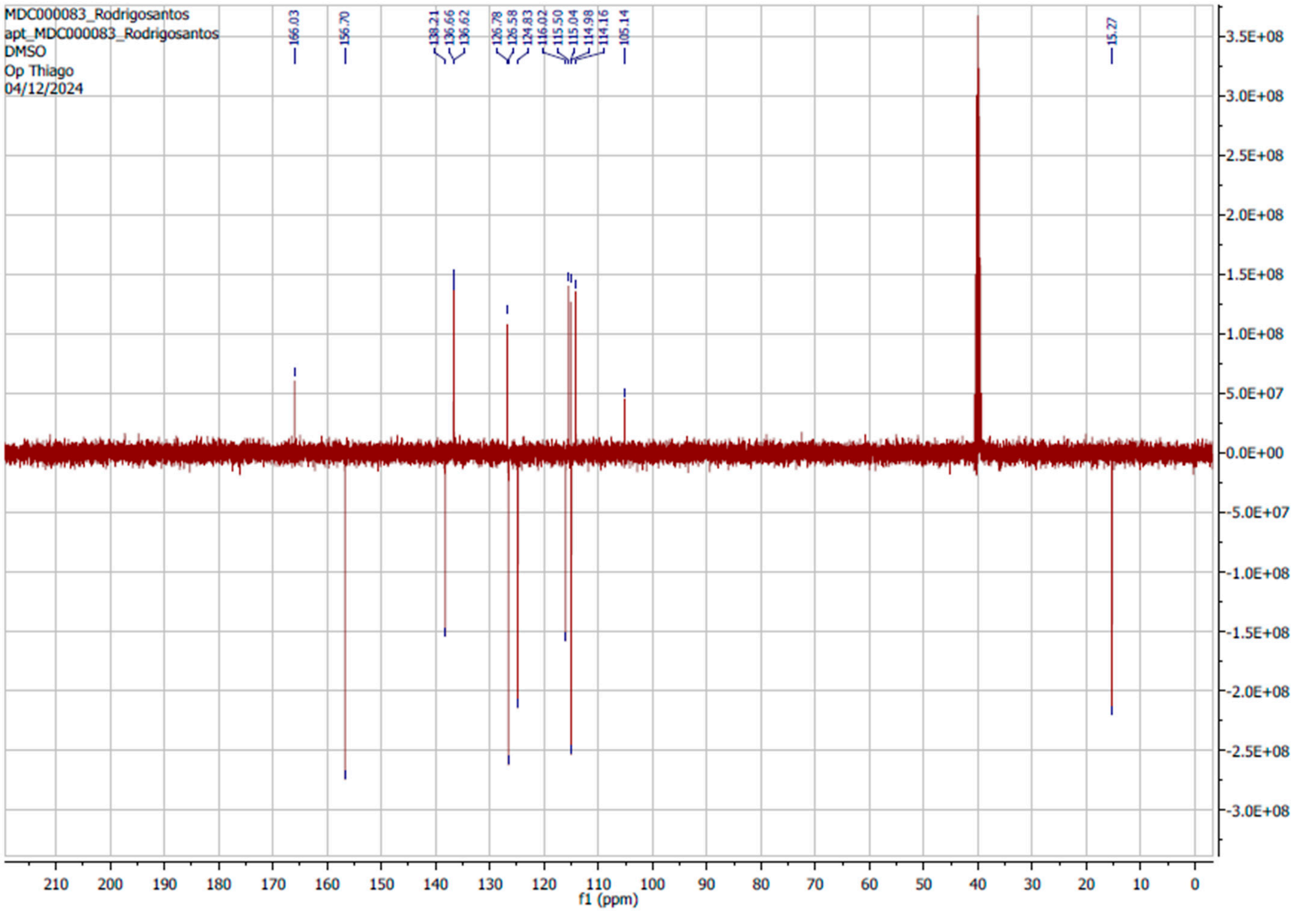

Figure S9: Mass spectra of 24

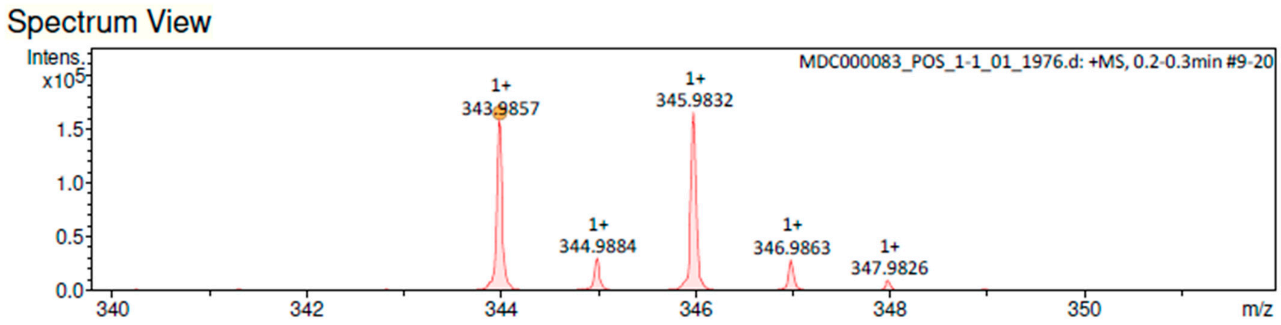

Figure S10: ^{1}H NMR full spectra of 27

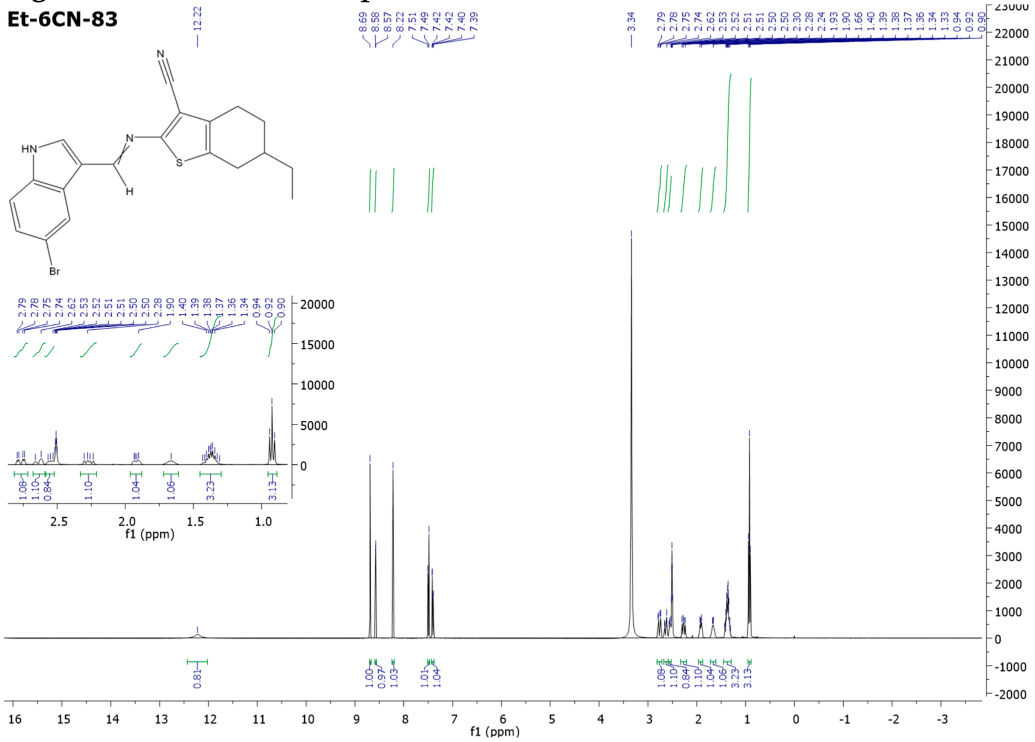

Figure S11: ^{13}C NMR spectra of 27

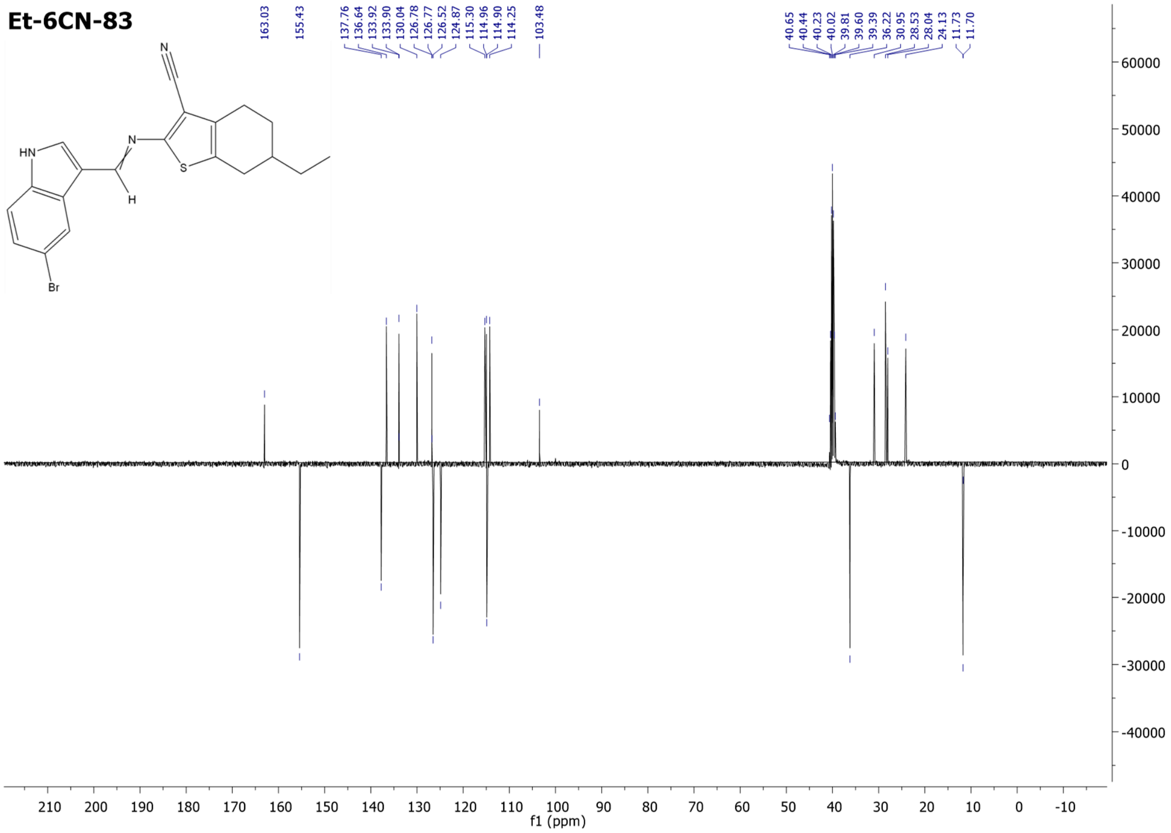

Figure S12: Mass spectra of 27

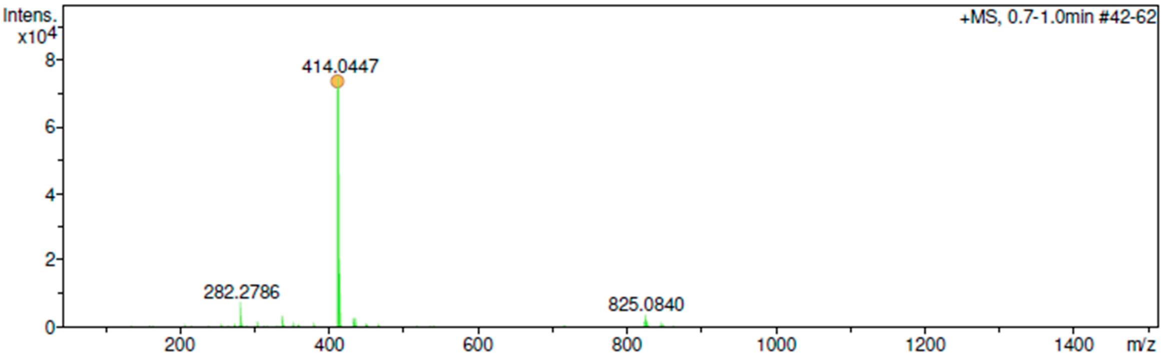

Figure S13: ^{1}H NMR full spectra of 28

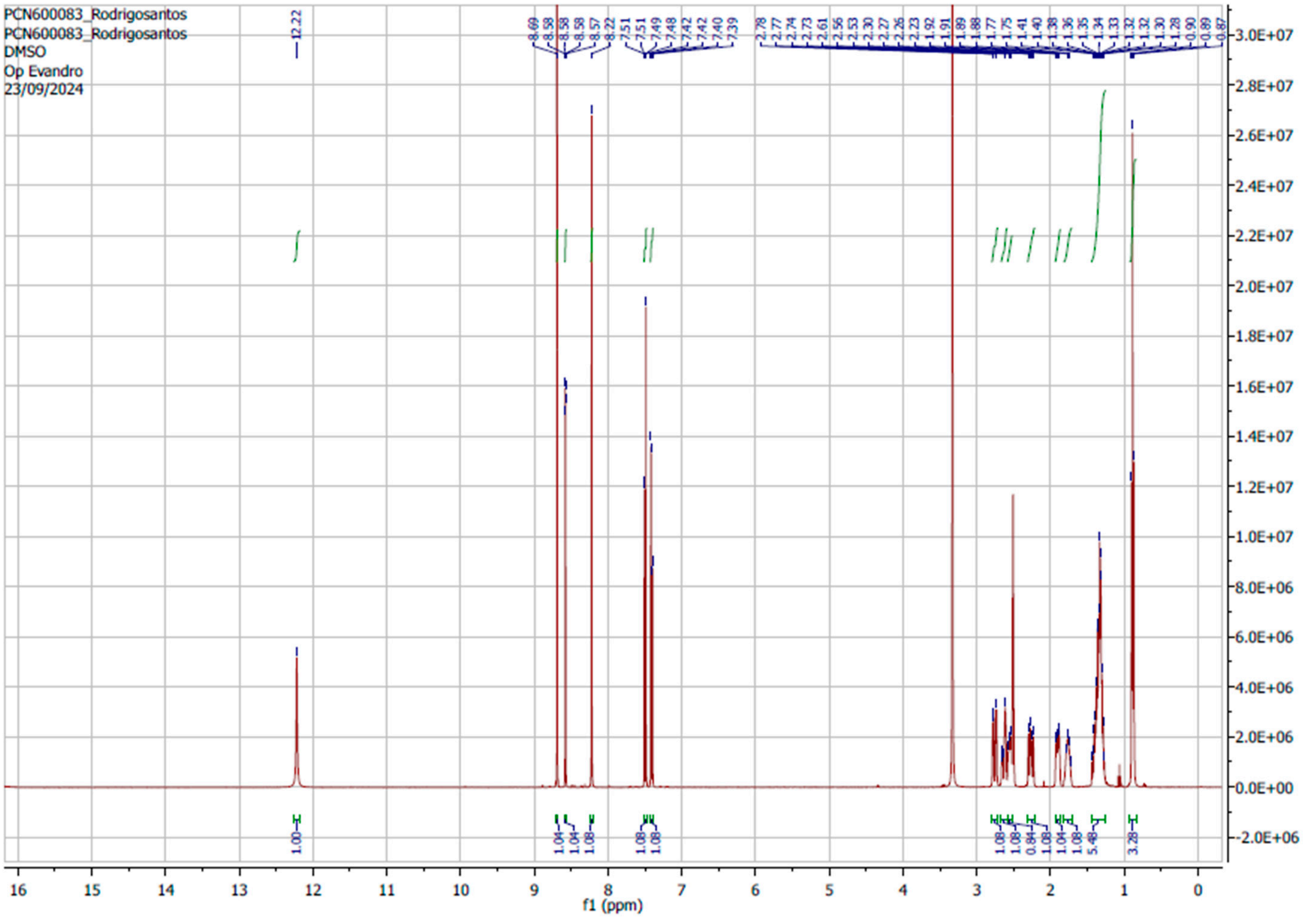

Figure S14: ^{1}H NMR expanded spectra of 28

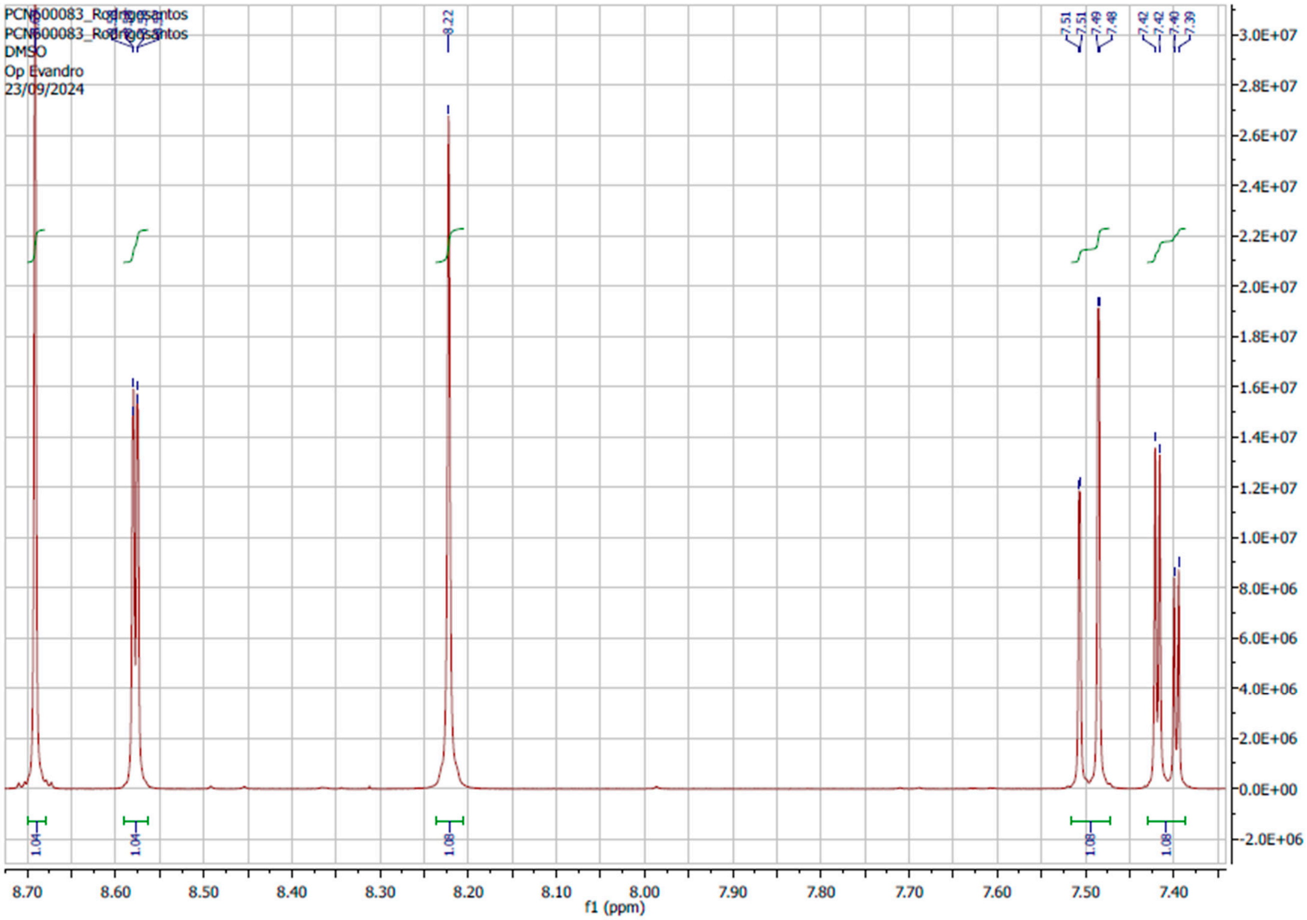

Figure S15: ^{1}H NMR expanded spectra of 28

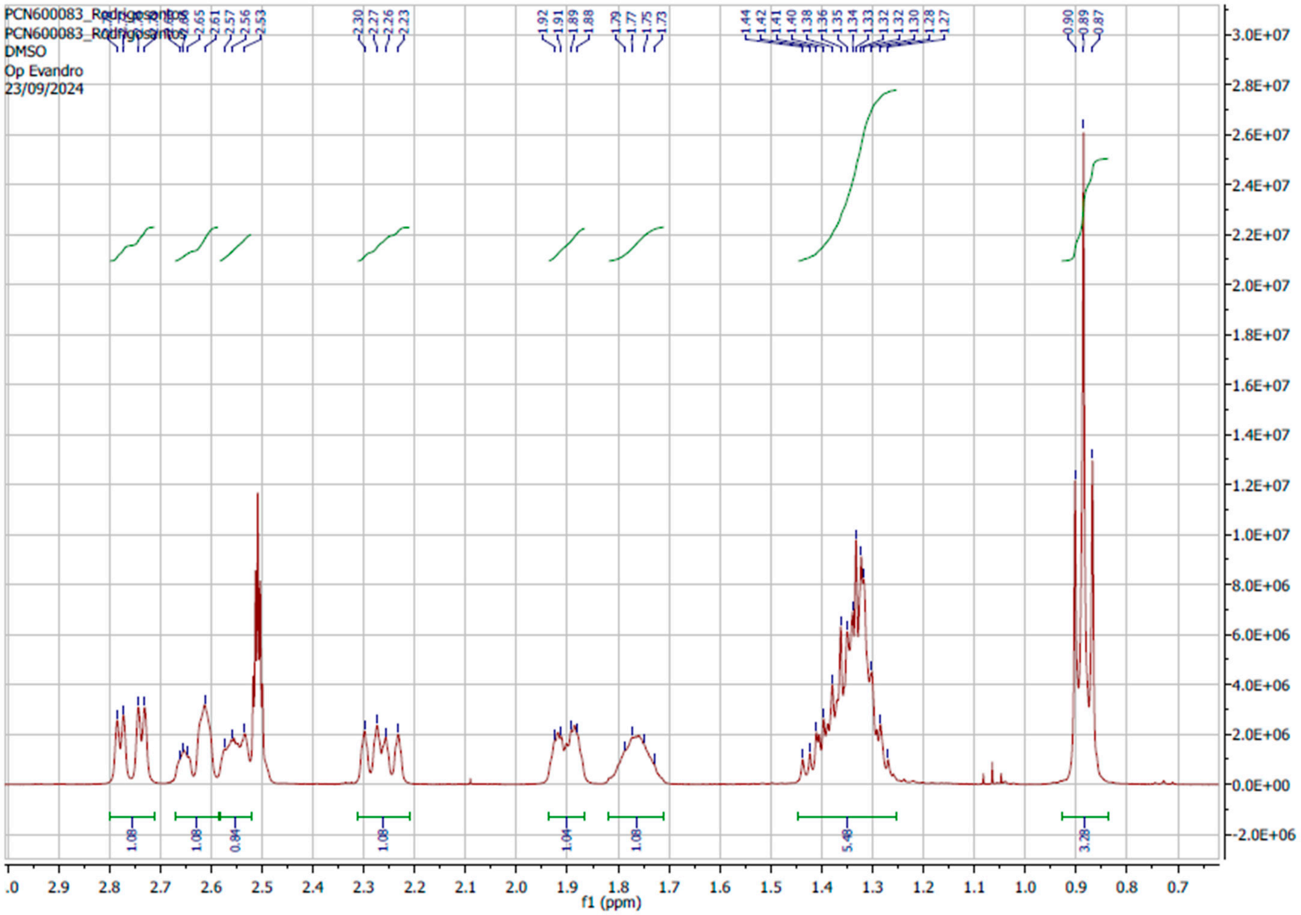

Figure S16: ^{13}C NMR spectra of 28

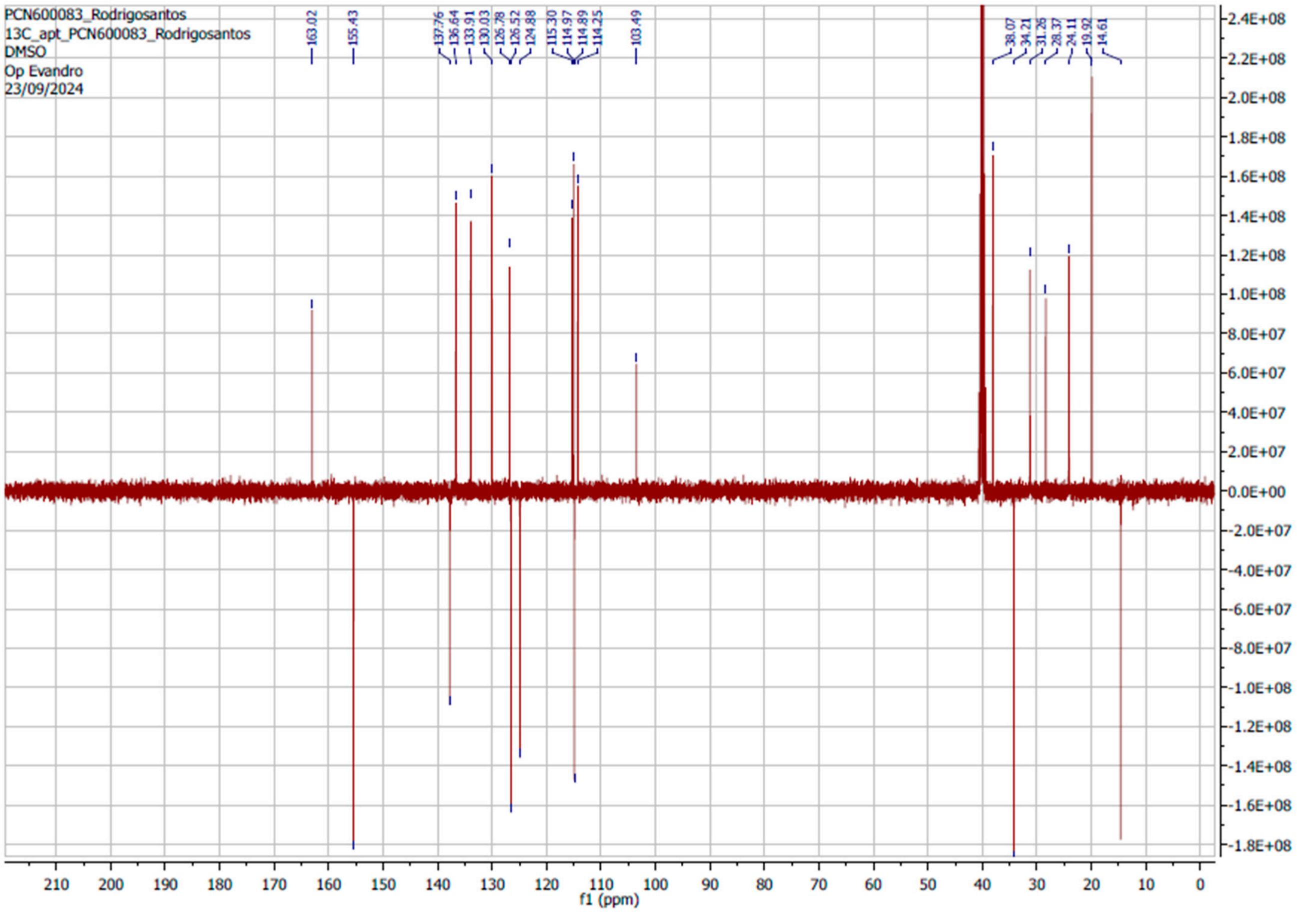

Figure S17: Mass spectra of 28

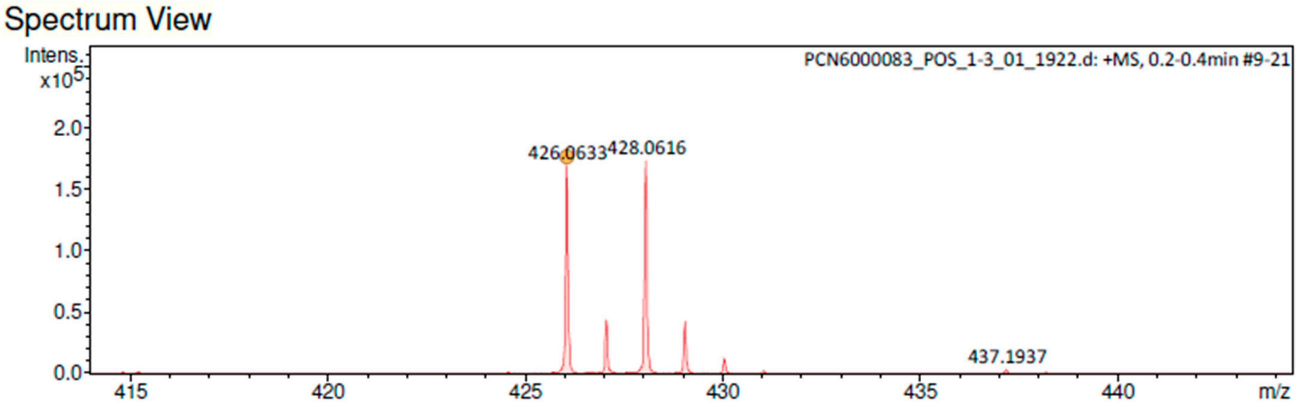

Figure S18: ^{1}H NMR full spectra of 29

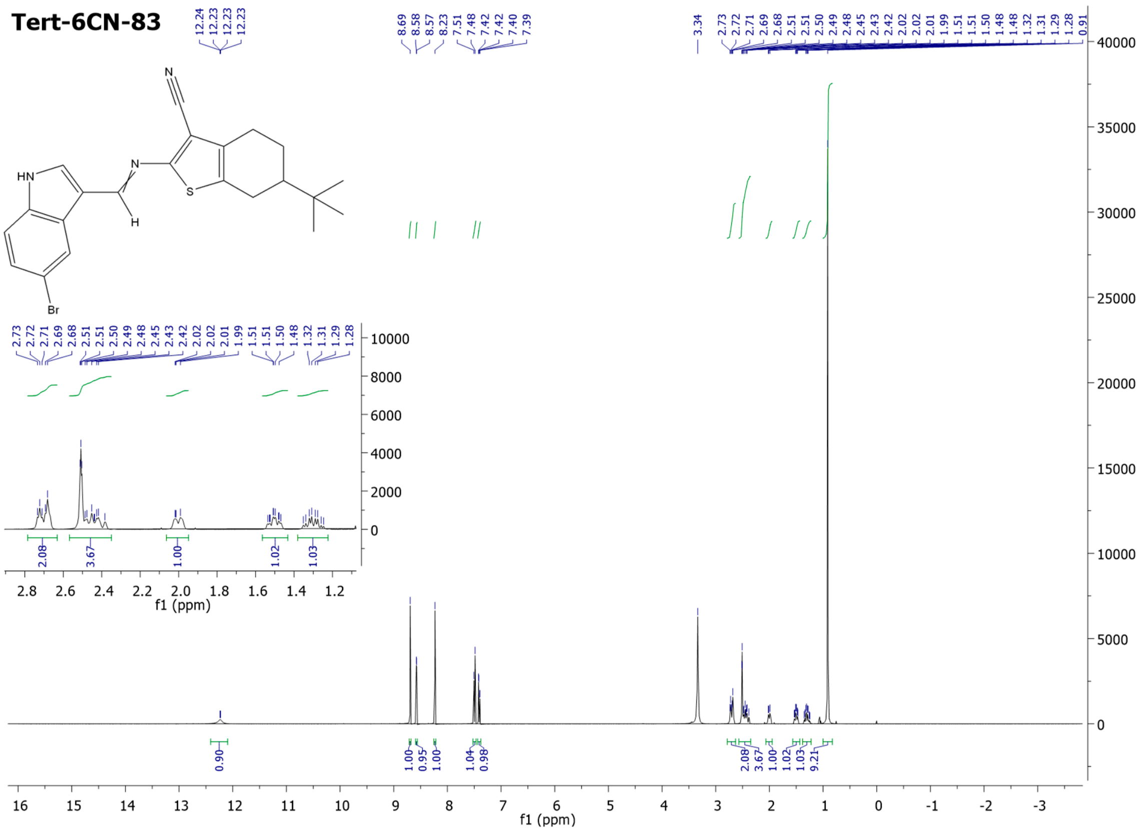

Figure S19: ^{13}C NMR spectra of 29

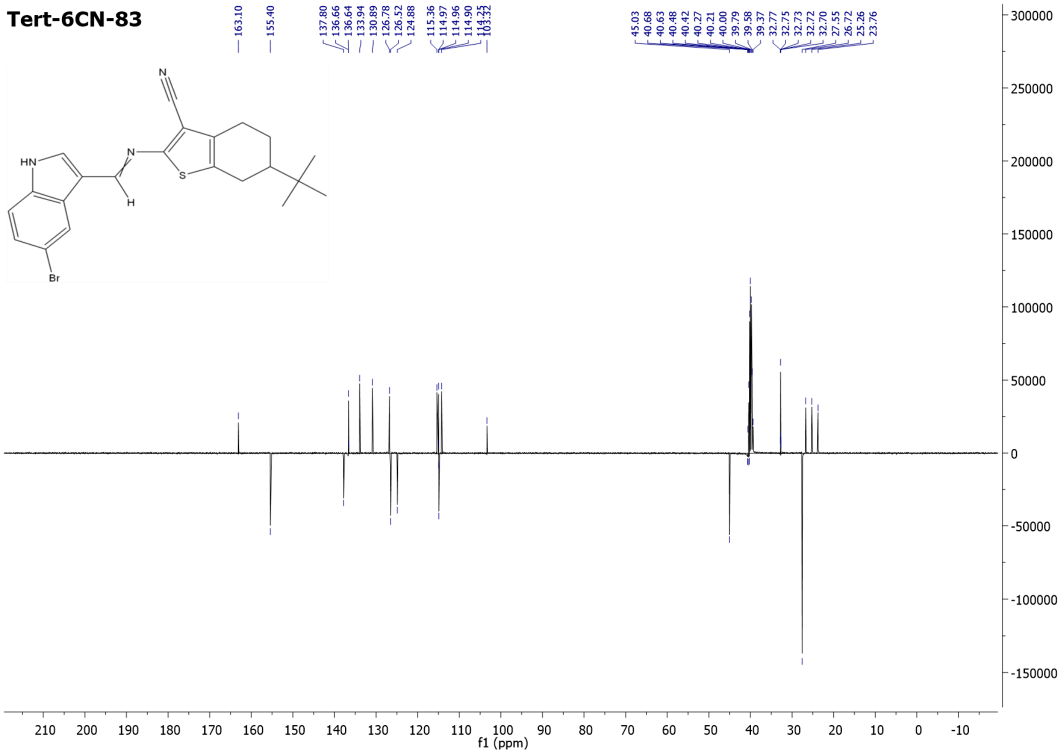

Figure S20: Mass spectra of 29

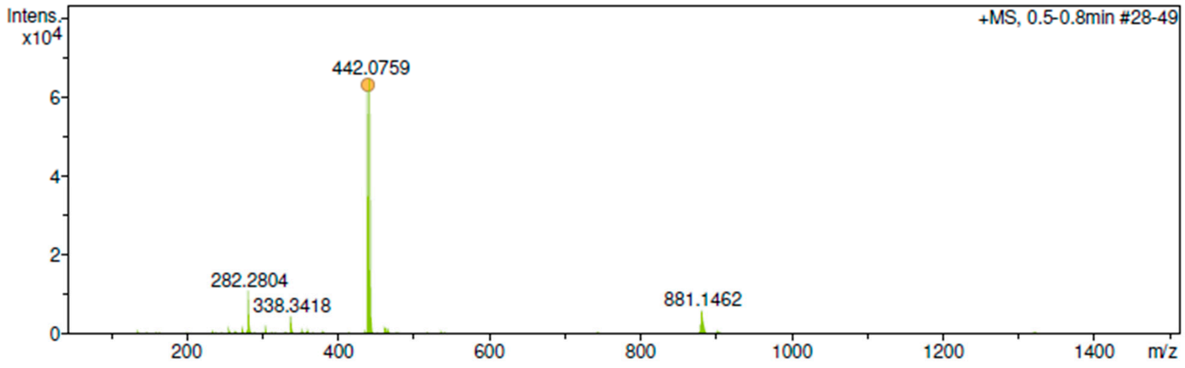

Figure S21: ^{1}H NMR full spectra of 31

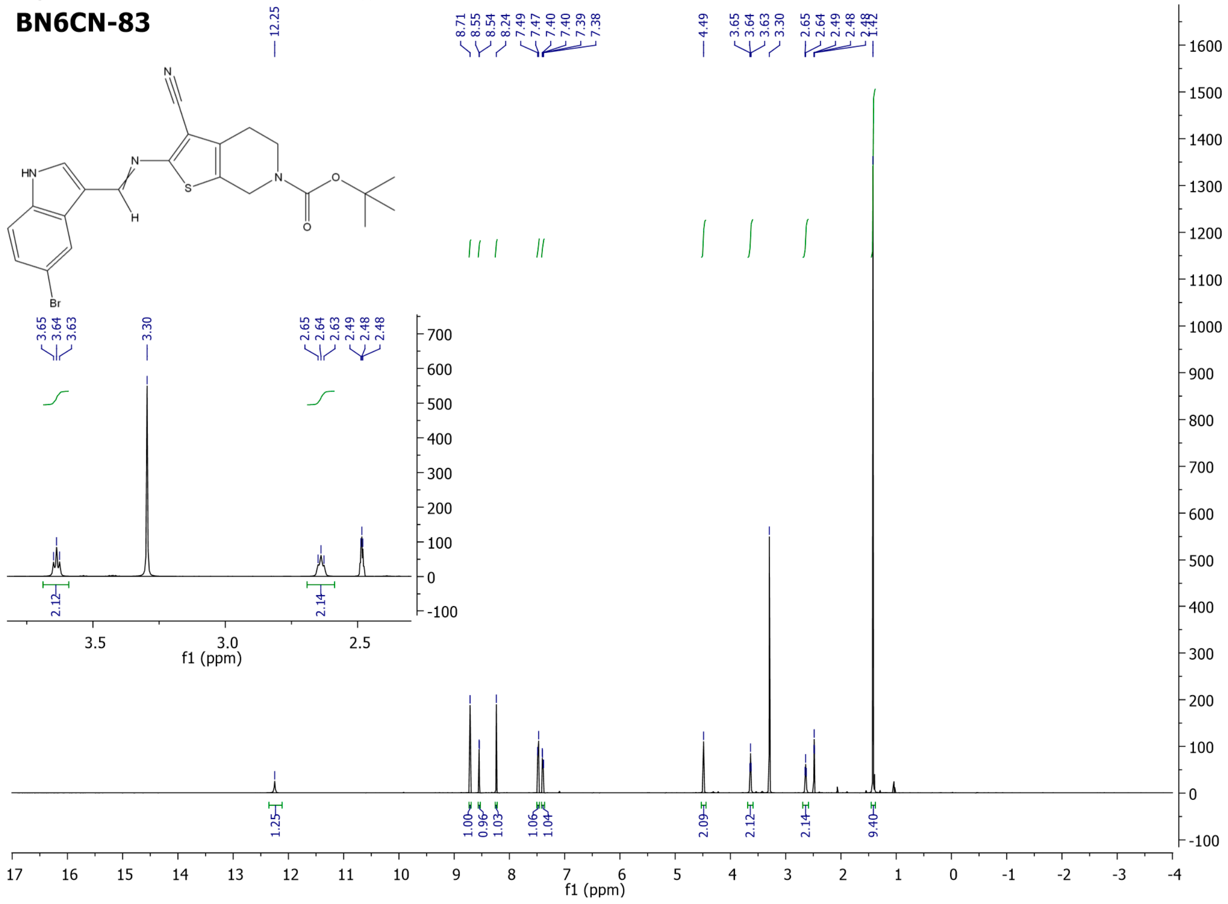

Figure S22: ¹³C NMR spectra of 31

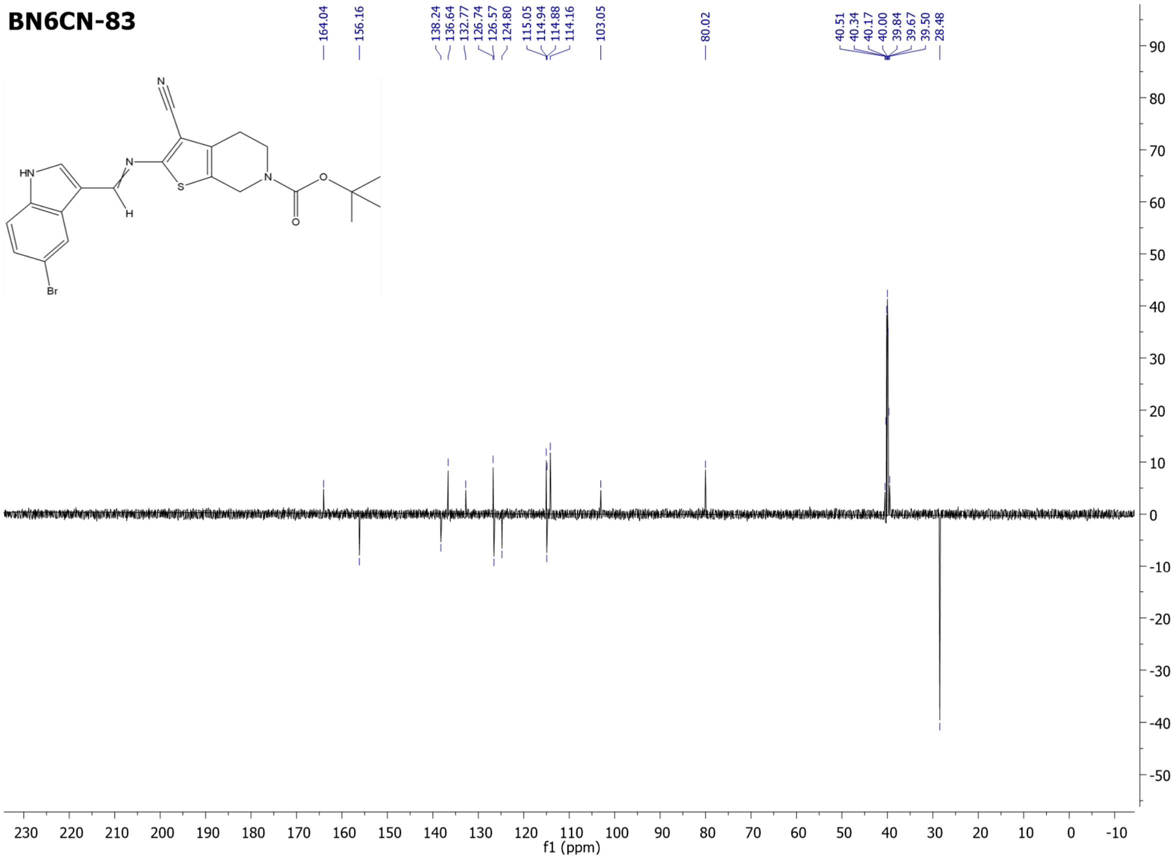

Figure S23: Mass spectra of 31

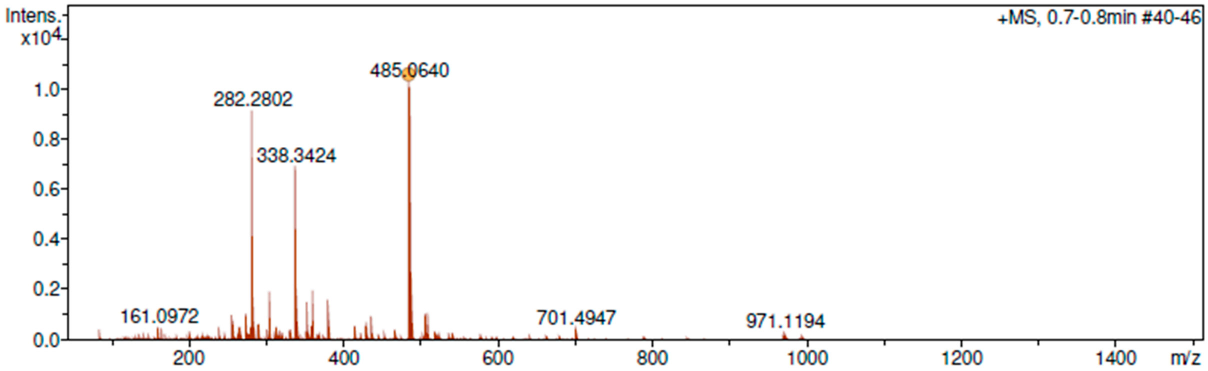

Figure S24: ^{1}H NMR full spectra of 32

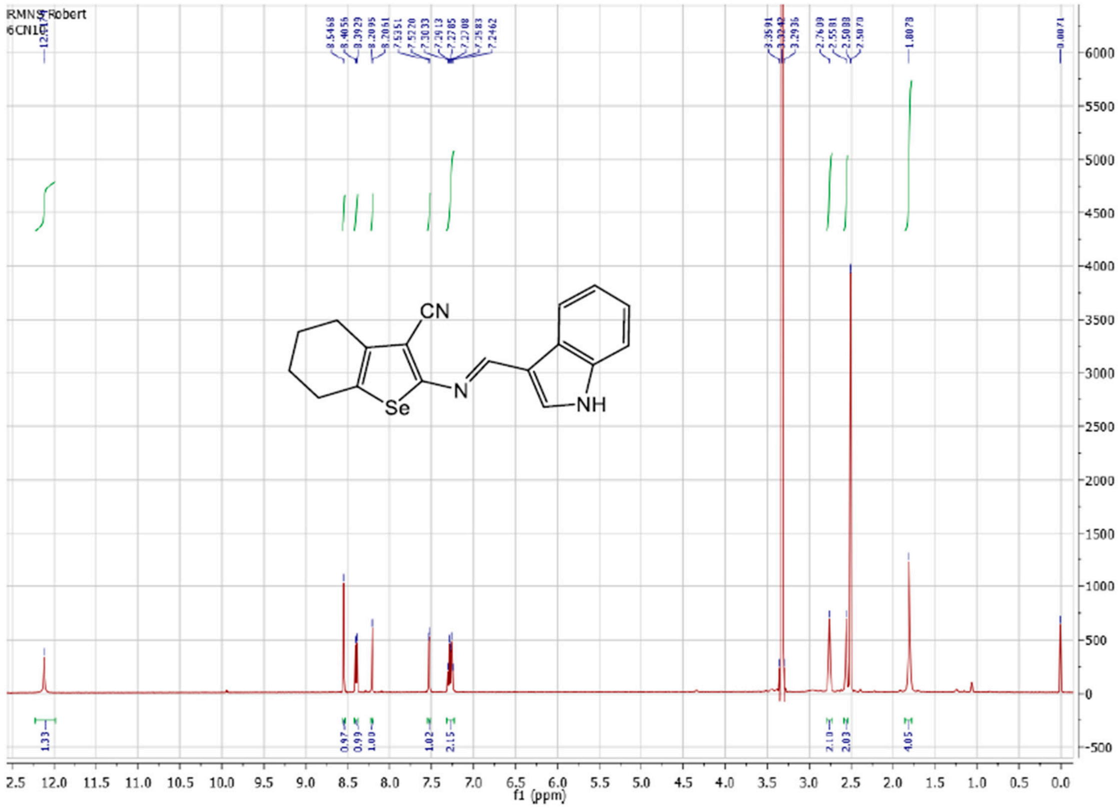

Figure S25: ^{1}H NMR expanded spectra of 32

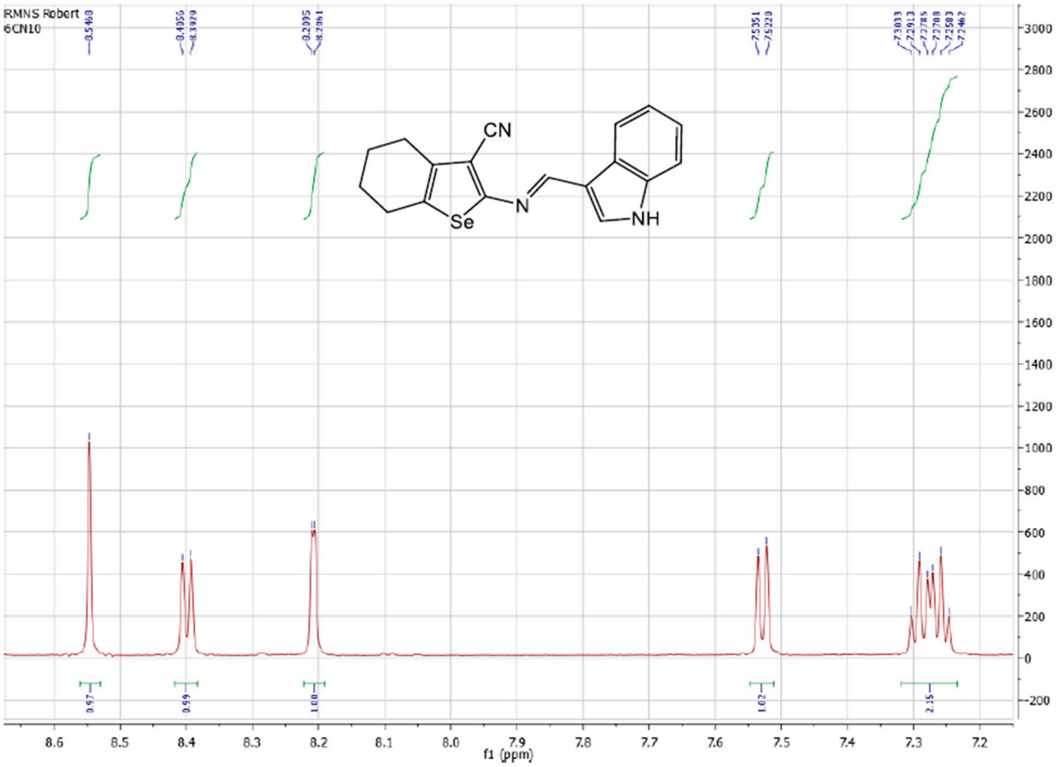

Figure S26: ^{13}C NMR spectra of 32

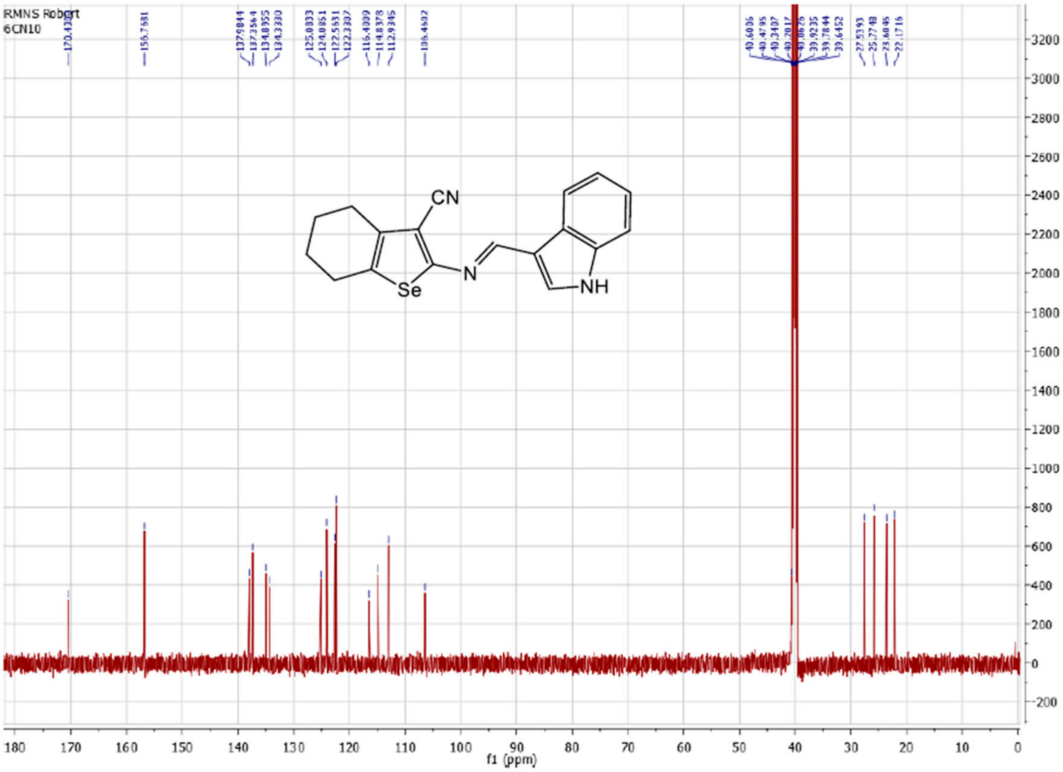

Figure S27: Mass spectra of 32

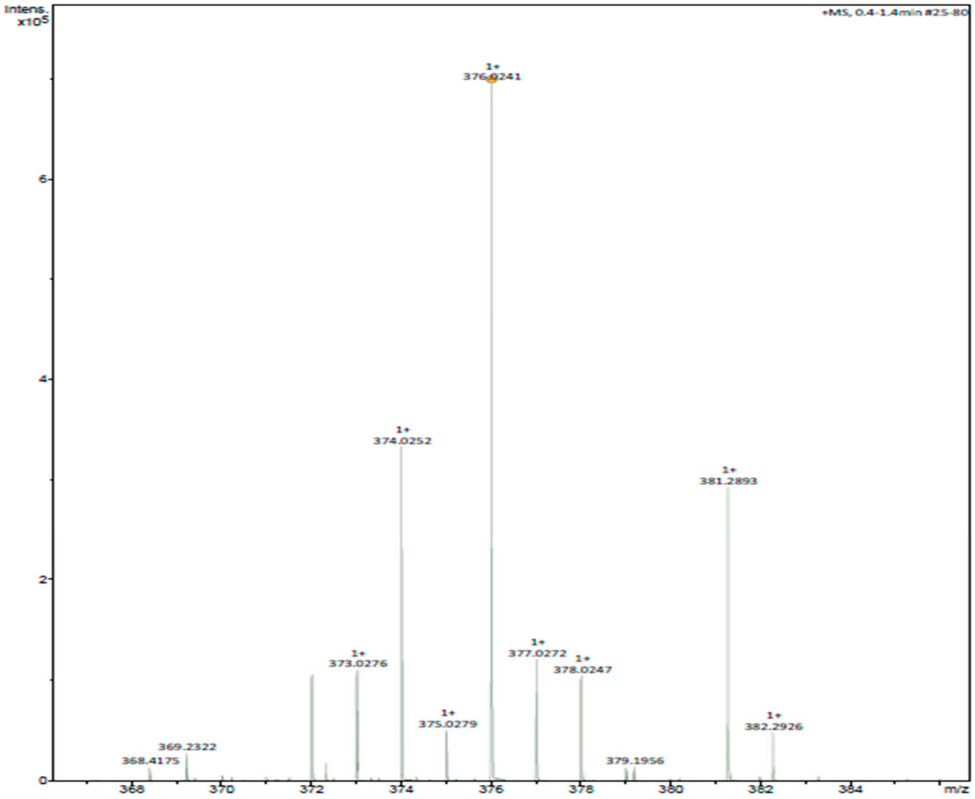

Figure S28: ¹H NMR full spectra of 33

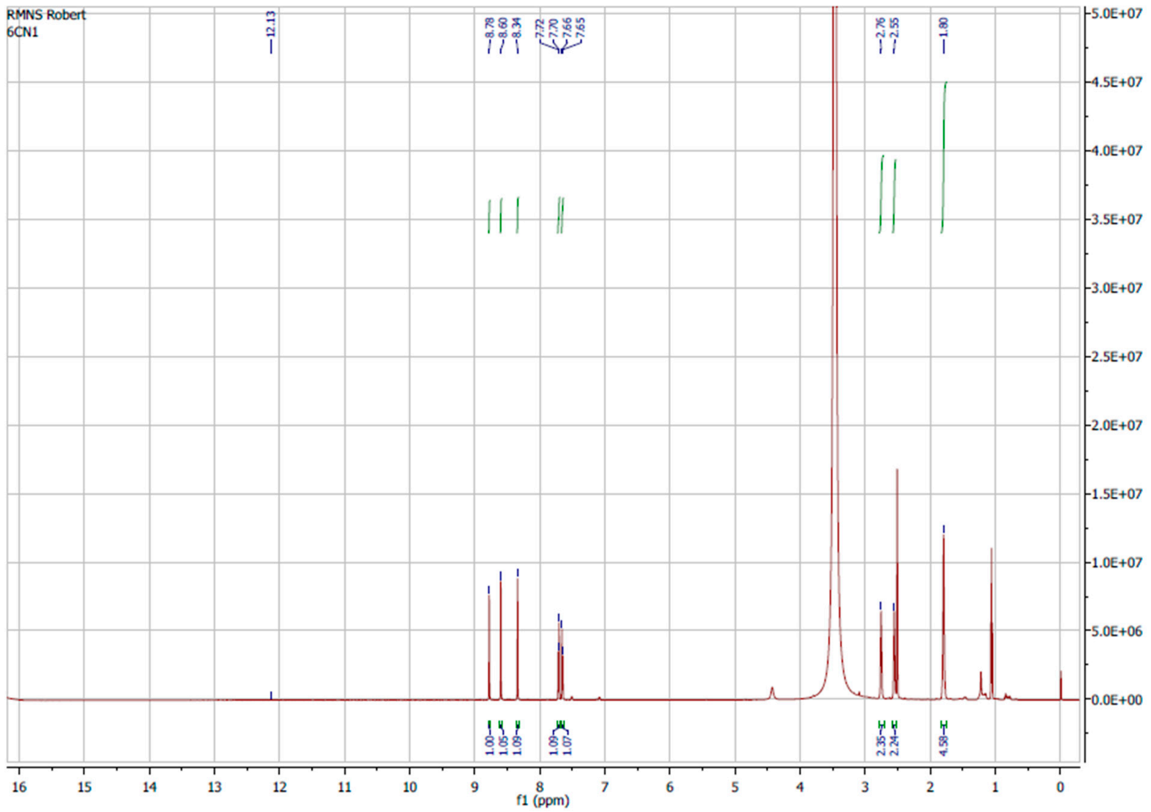

Figure S29: ¹H NMR expanded spectra of 33

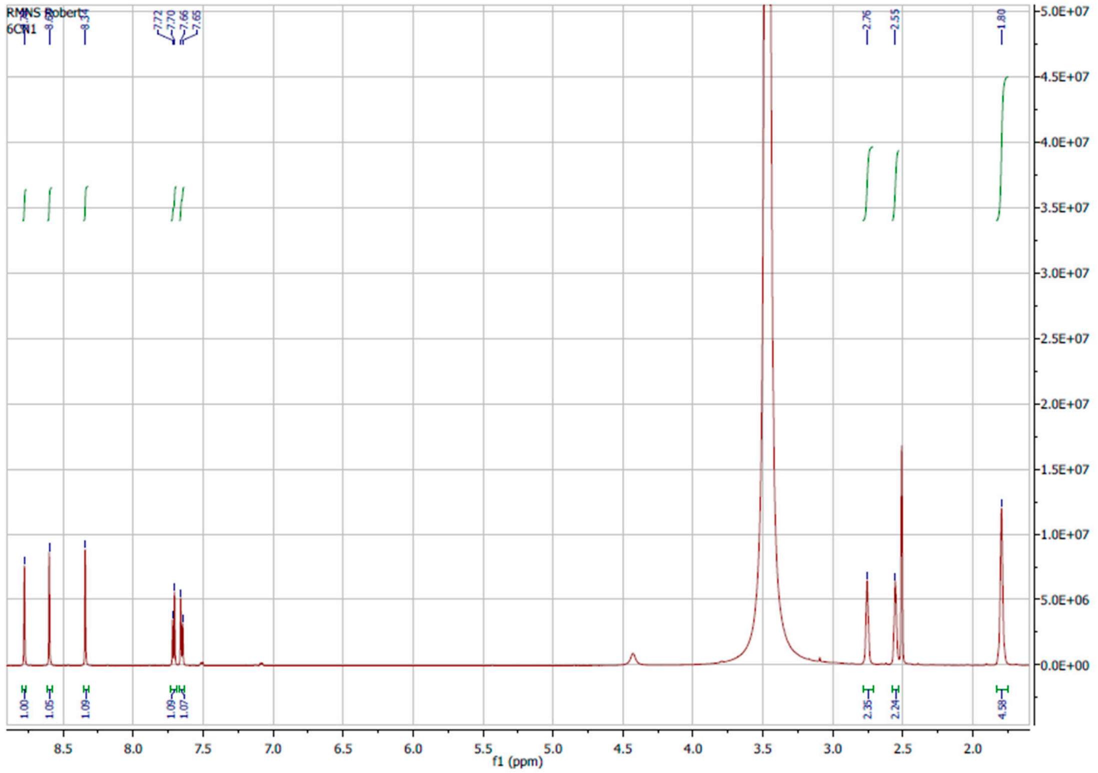

Figure S30: ^{13}C NMR spectra of 33

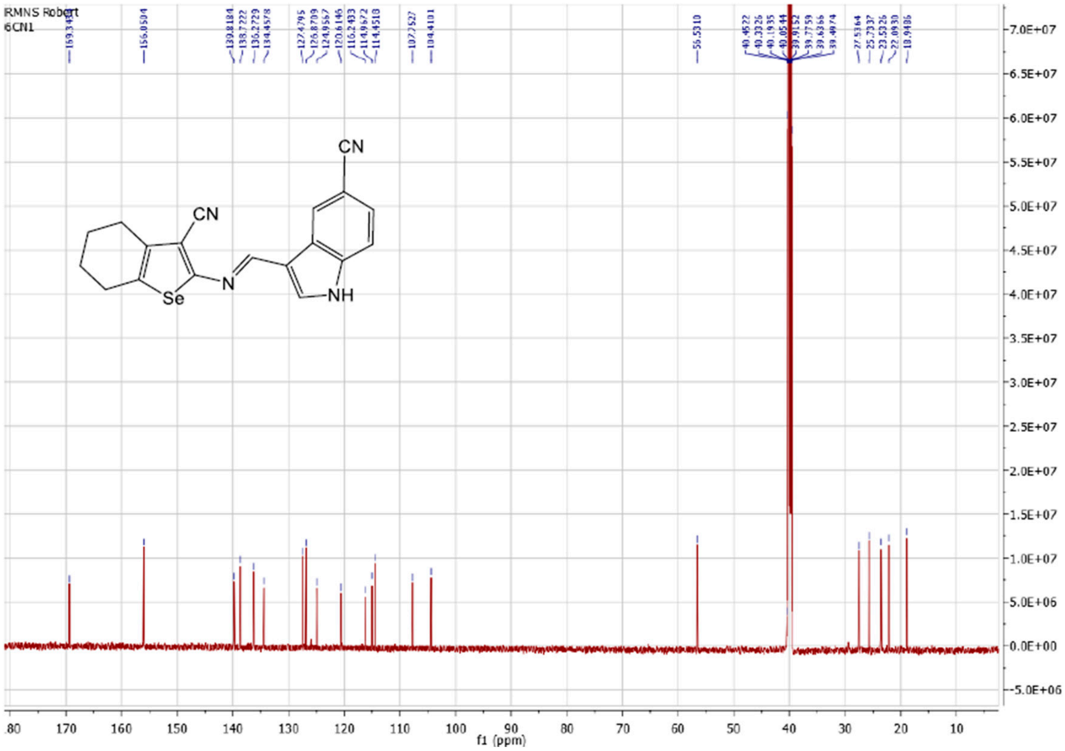

Figure S31: Mass spectra of 33

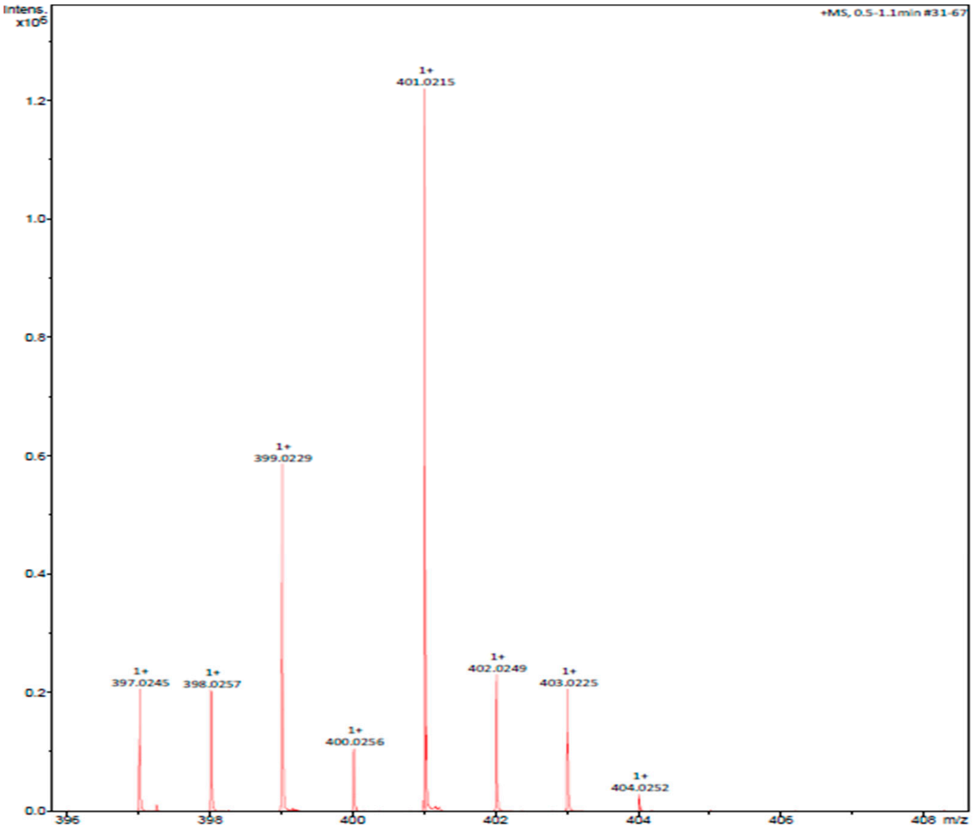

Figure S32: ^{1}H NMR full spectra of 34

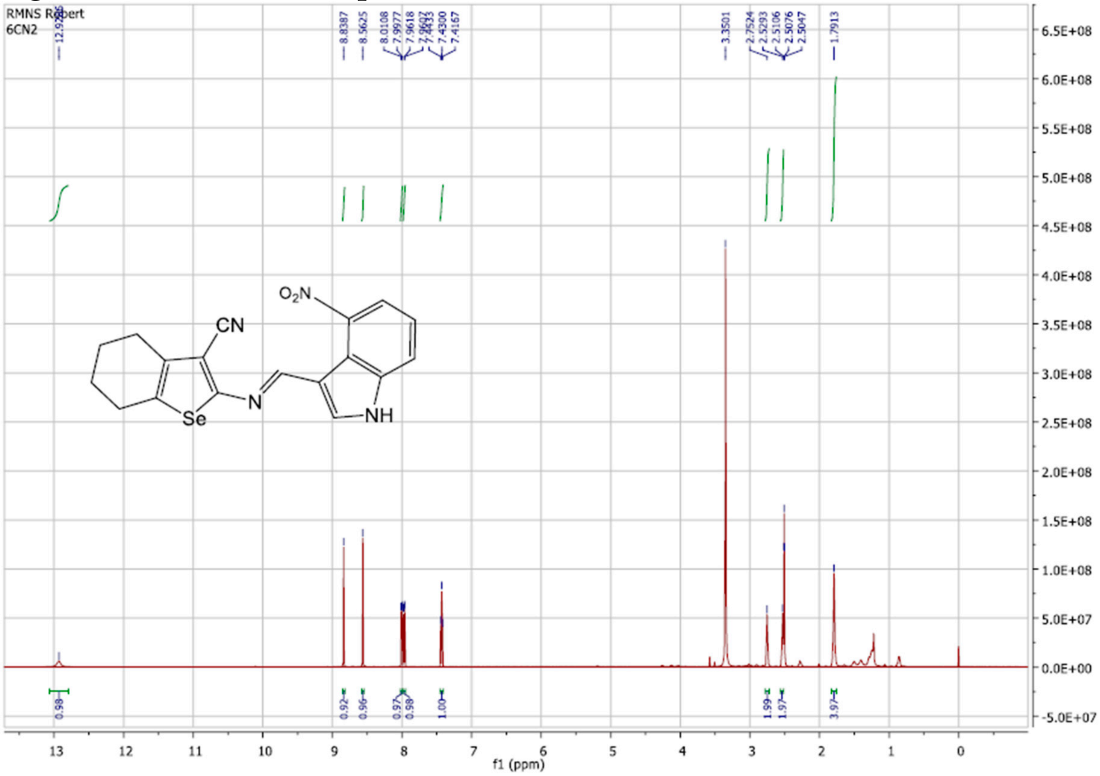

Figure S33: ^{1}H NMR expanded spectra of 34

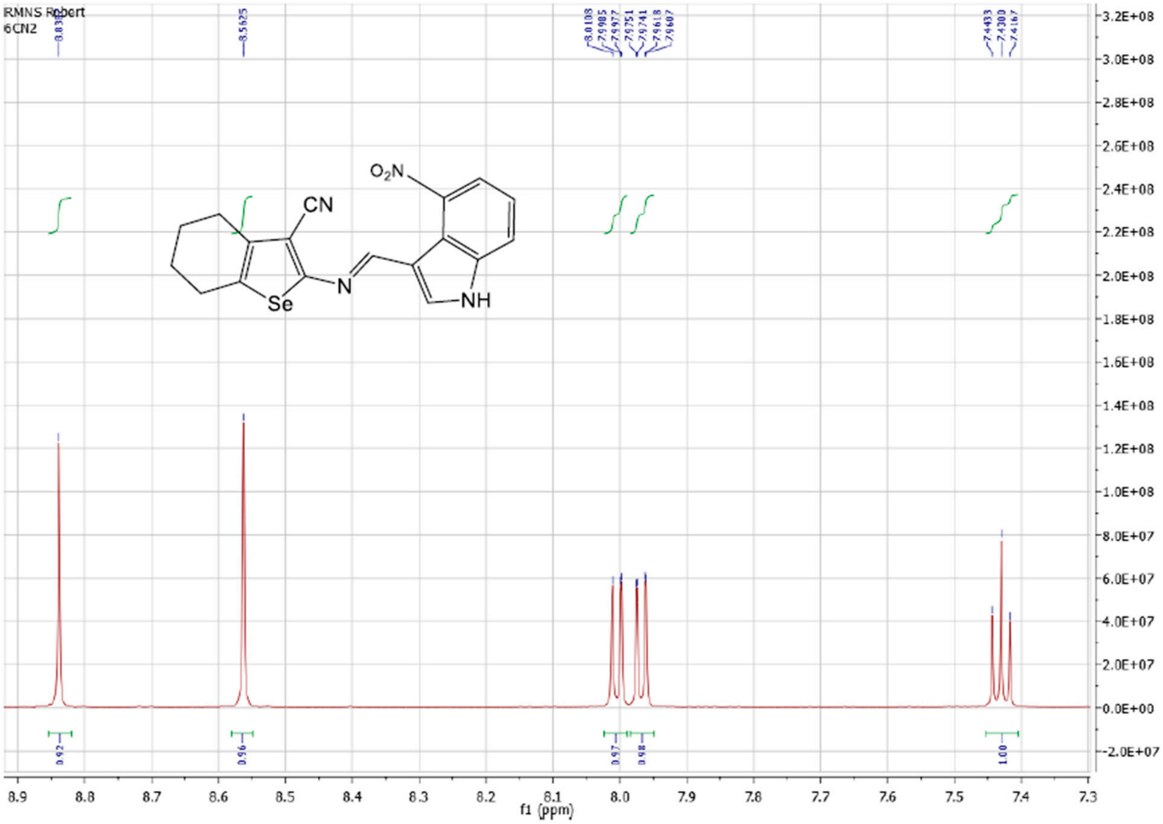

Figure S34: ^{13}C NMR spectra of 34

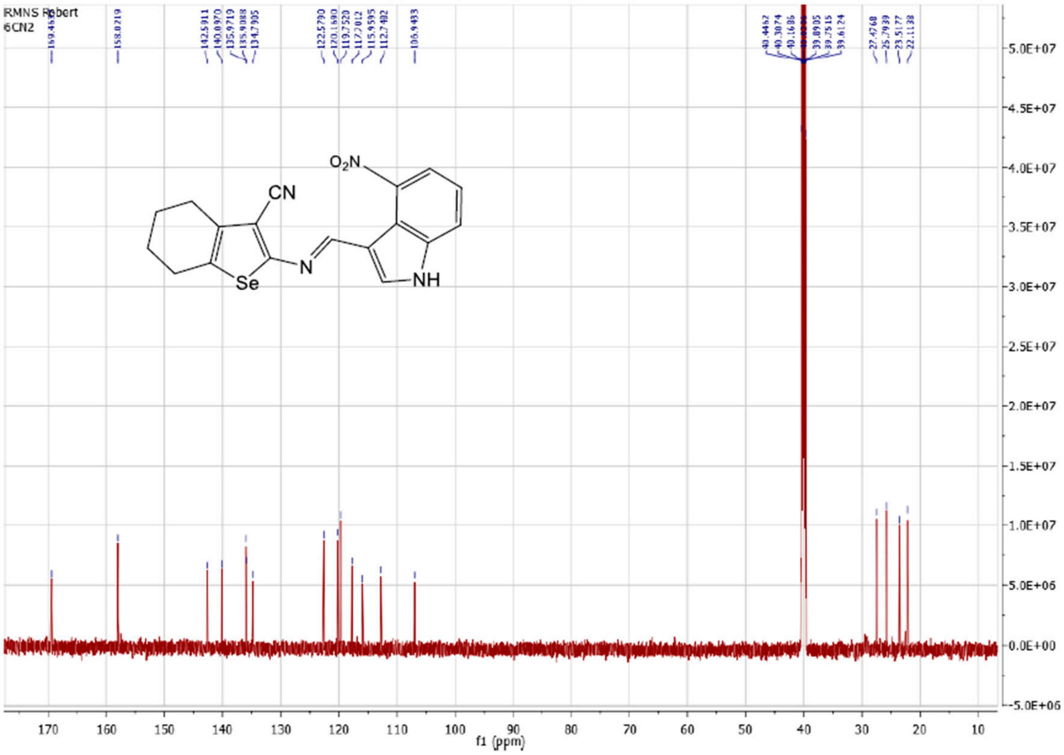

Figure S35: Mass spectra of 34

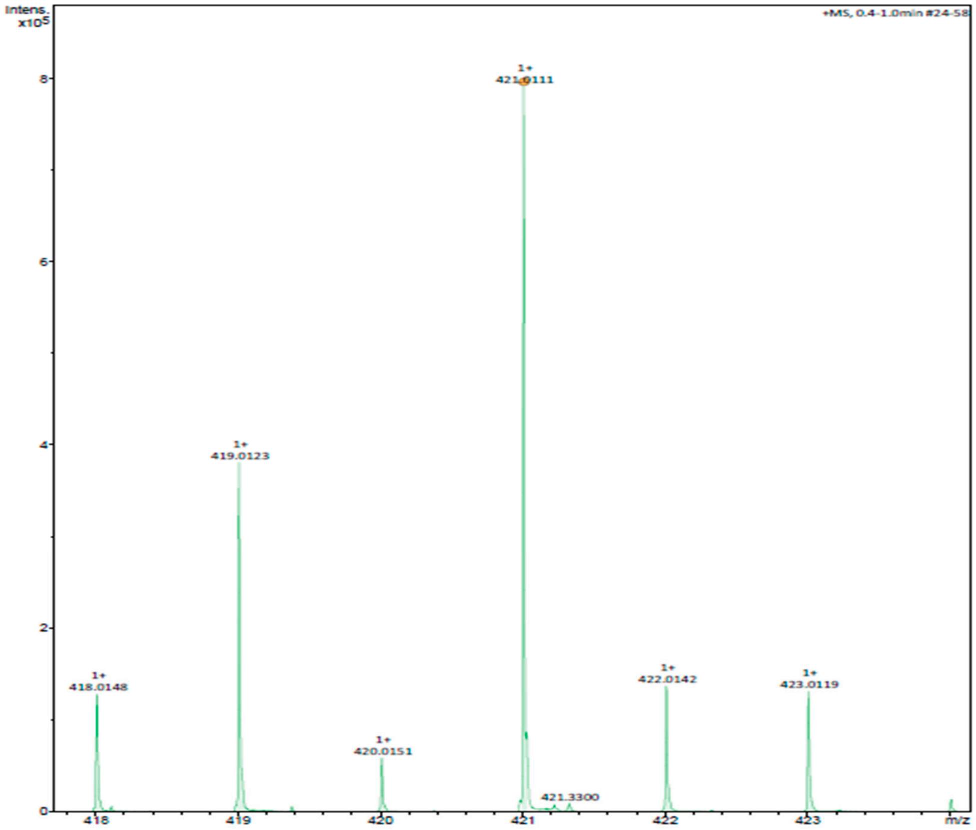

Figure S36: ^{1}H NMR full spectra of 35

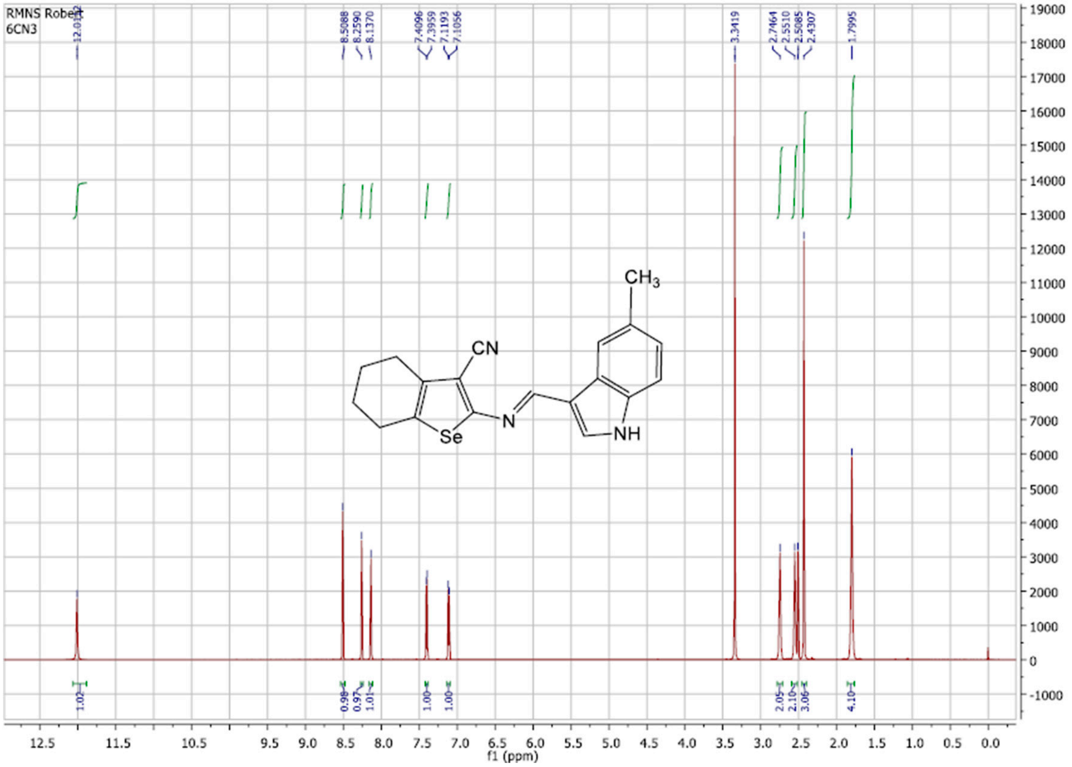

Figure S37: ^{1}H NMR expanded spectra of 35

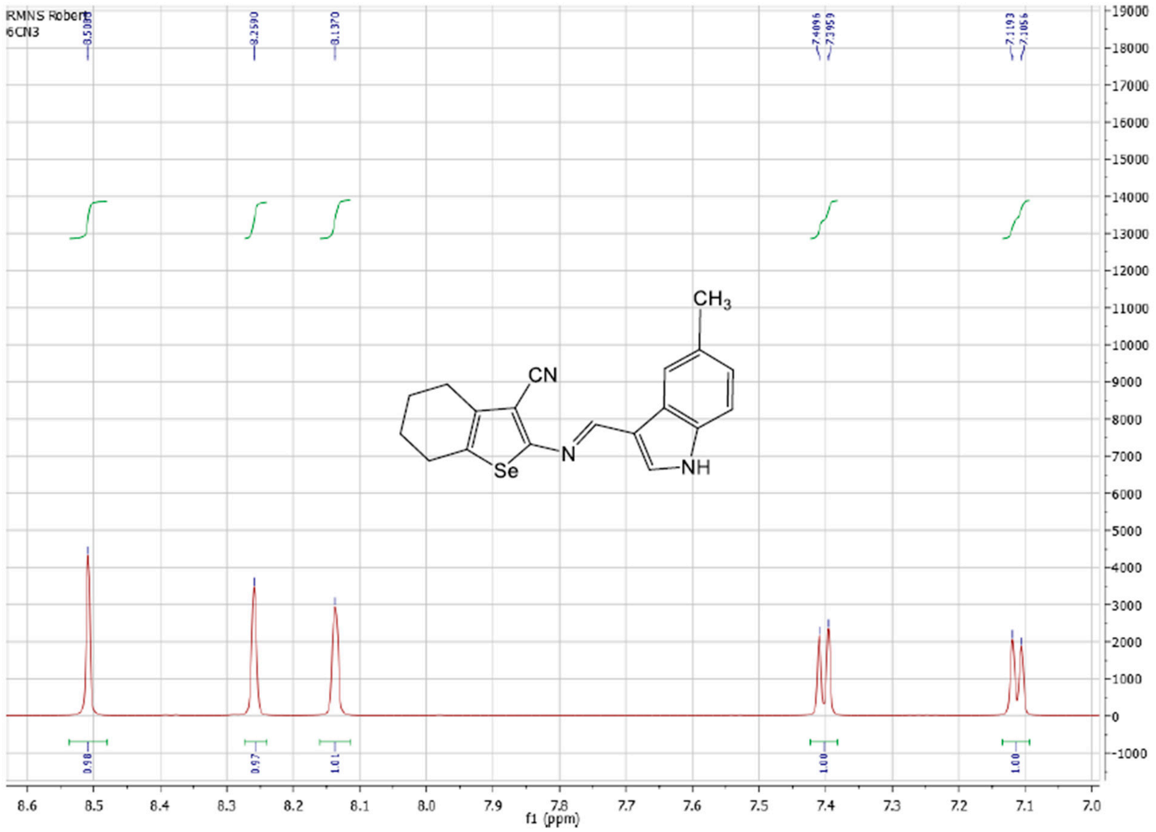

Figure S38: ^{13}C NMR spectra of 35

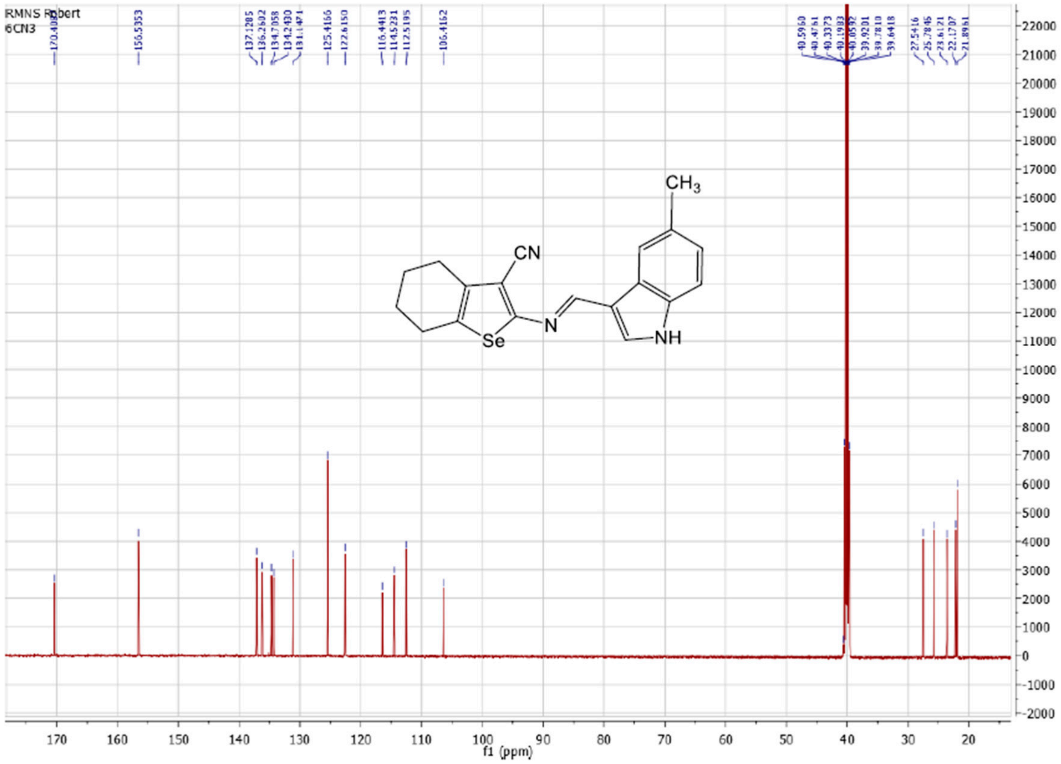

Figure S39: Mass spectra of 35

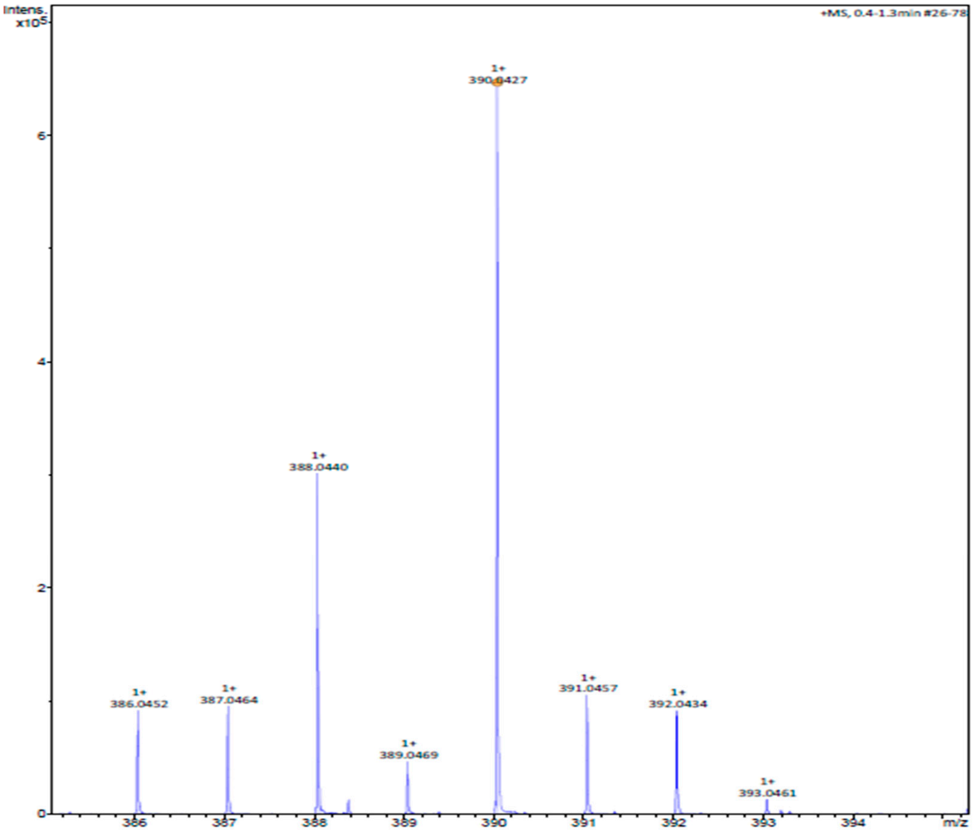

Figure S40: ¹H NMR full spectra of 36

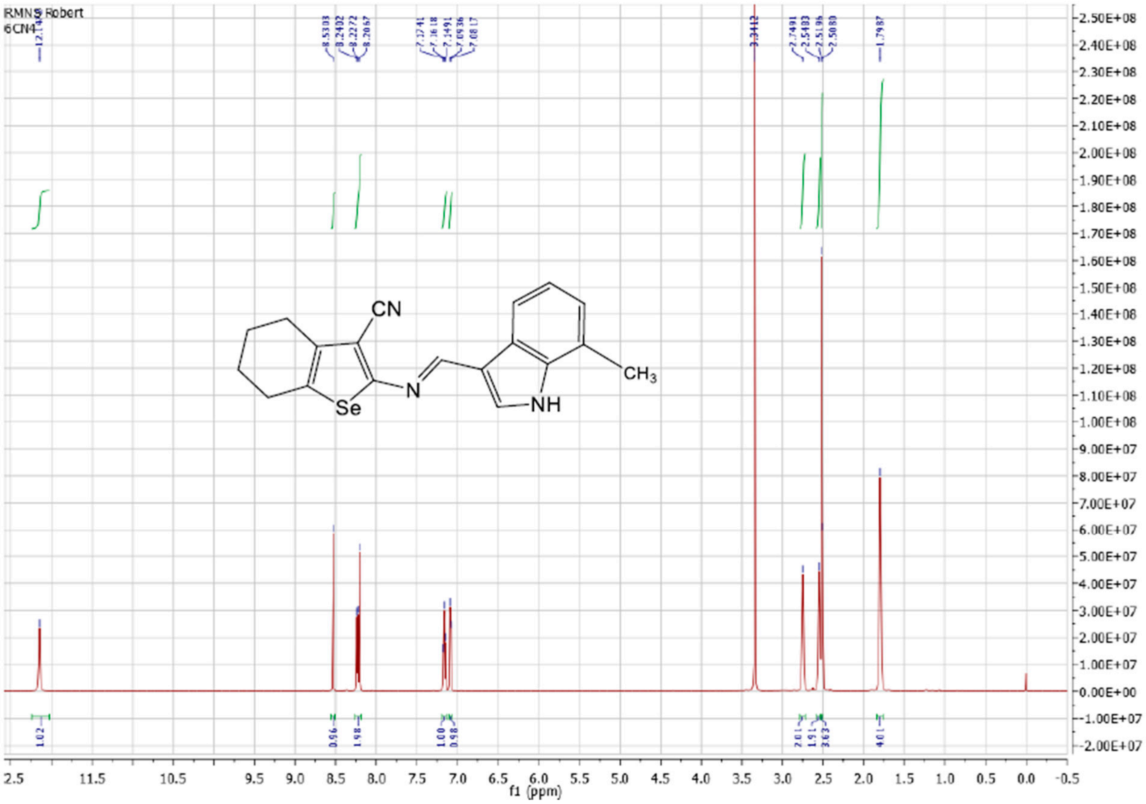

Figure S41: ¹H NMR expanded spectra of 36

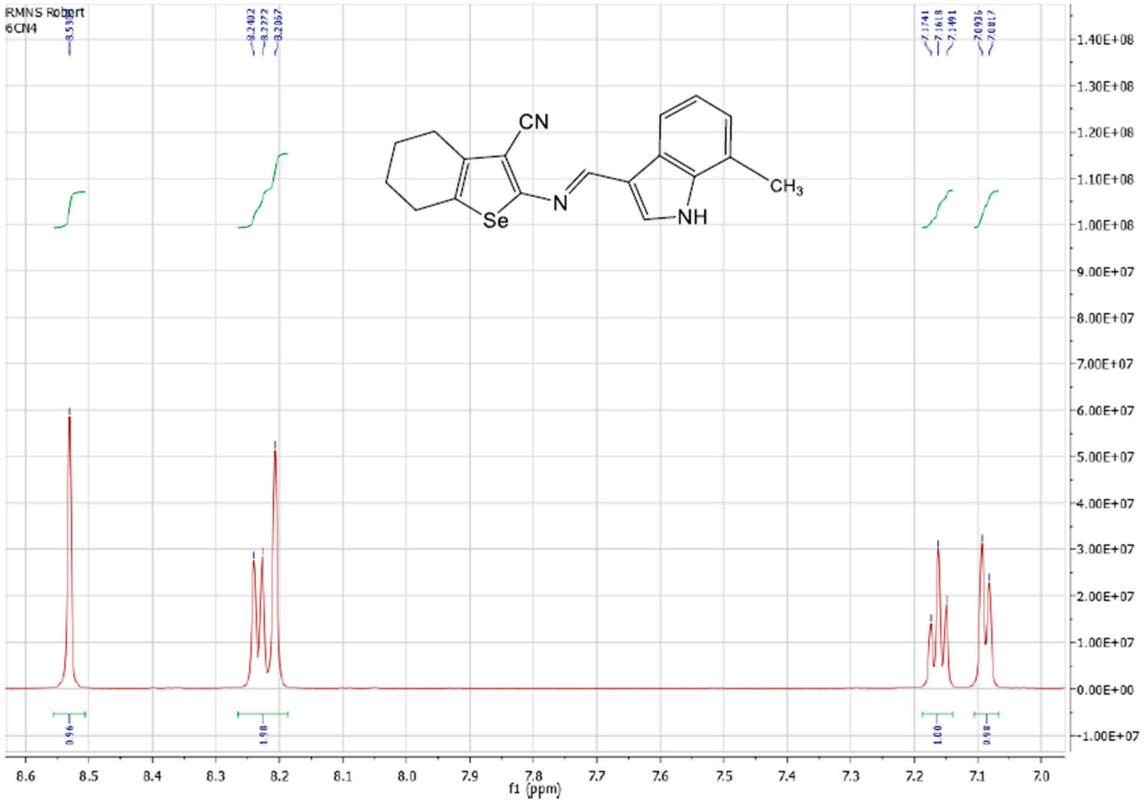

Figure S42: ^{13}C NMR spectra of 36

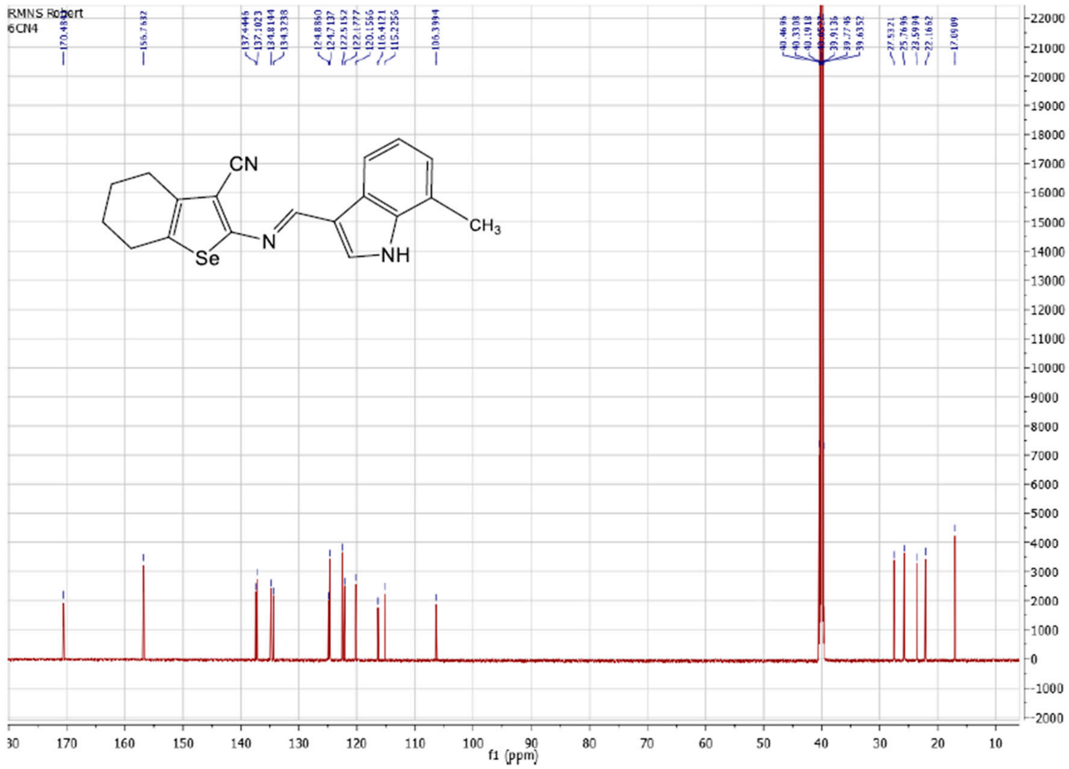

Figure S43: Mass spectra of 36

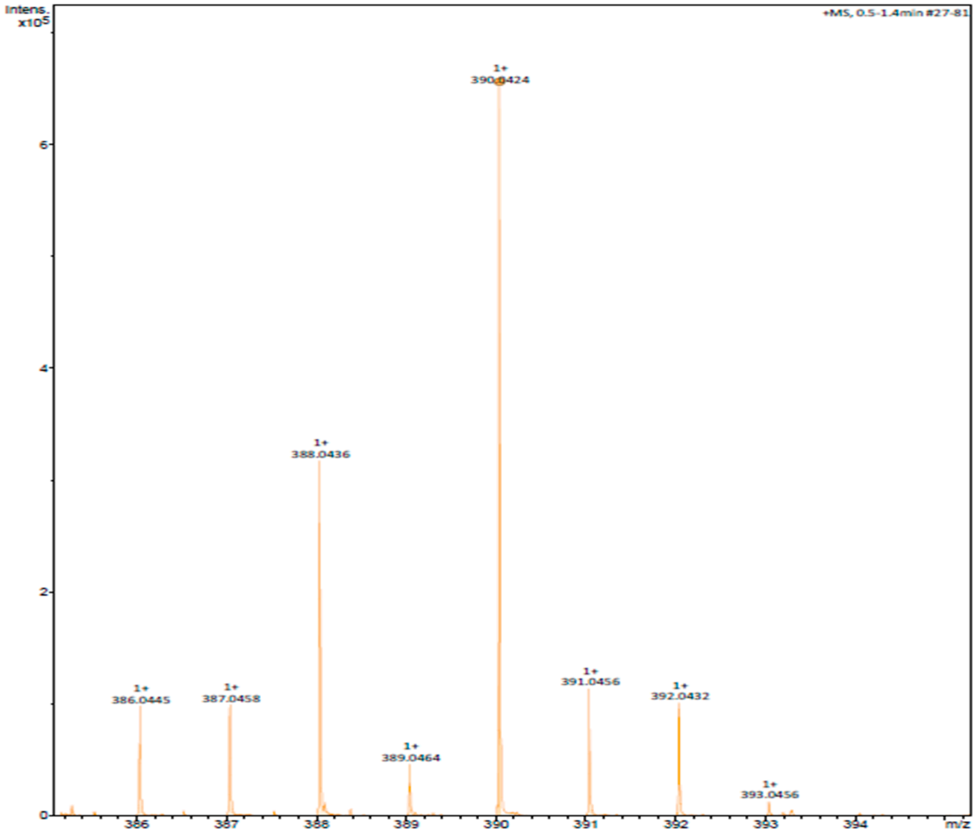

Figure S44: ^{1}H NMR full spectra of 37

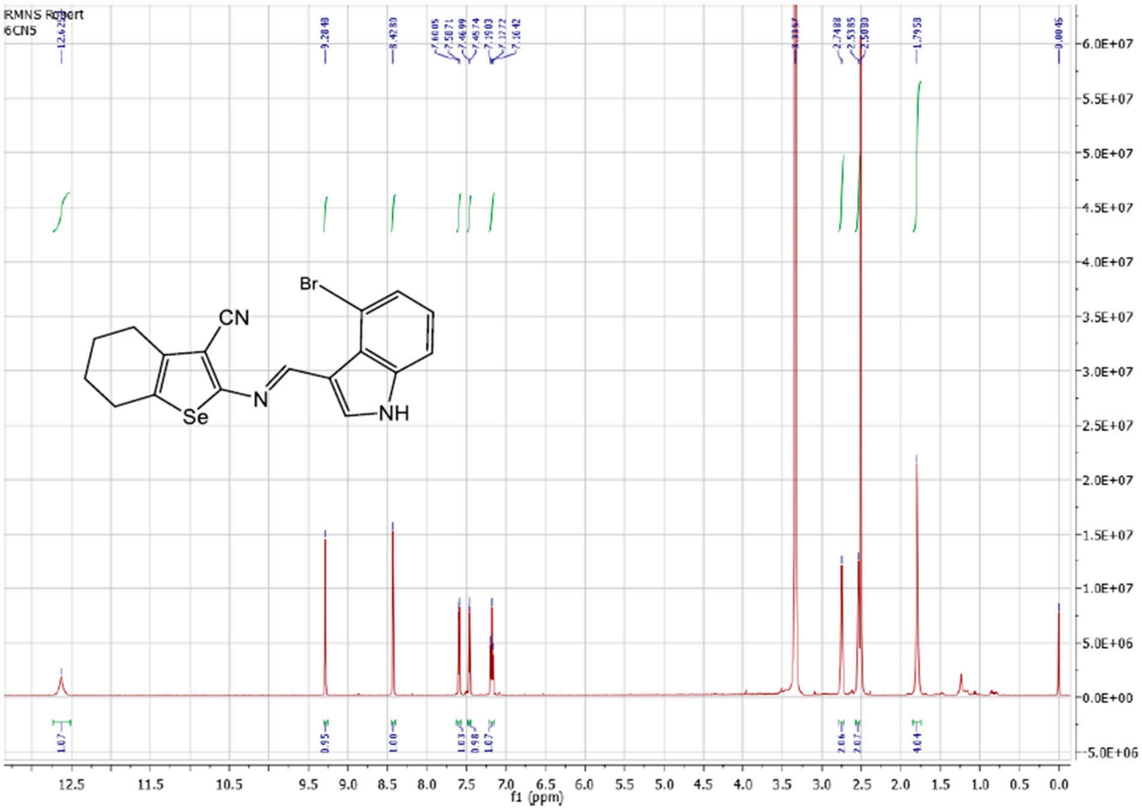

Figure S45: ^{1}H NMR expanded spectra of 37

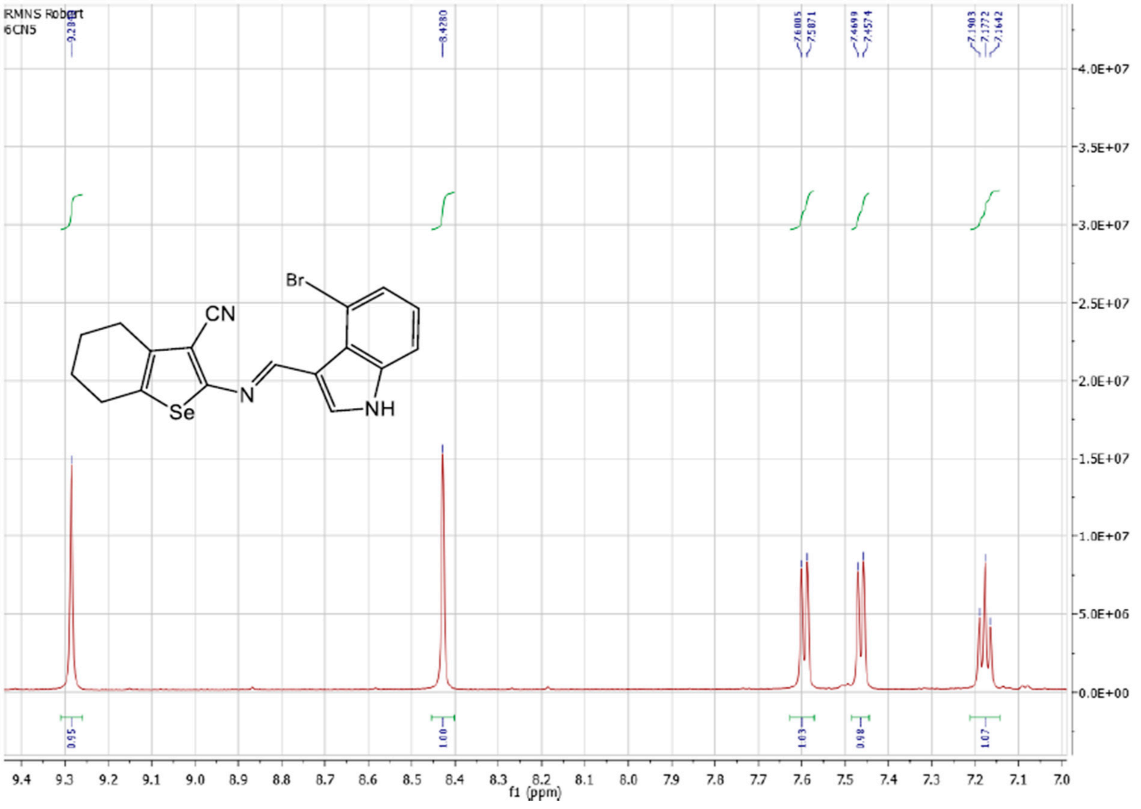

Figure S46: ^{13}C NMR spectra of 37

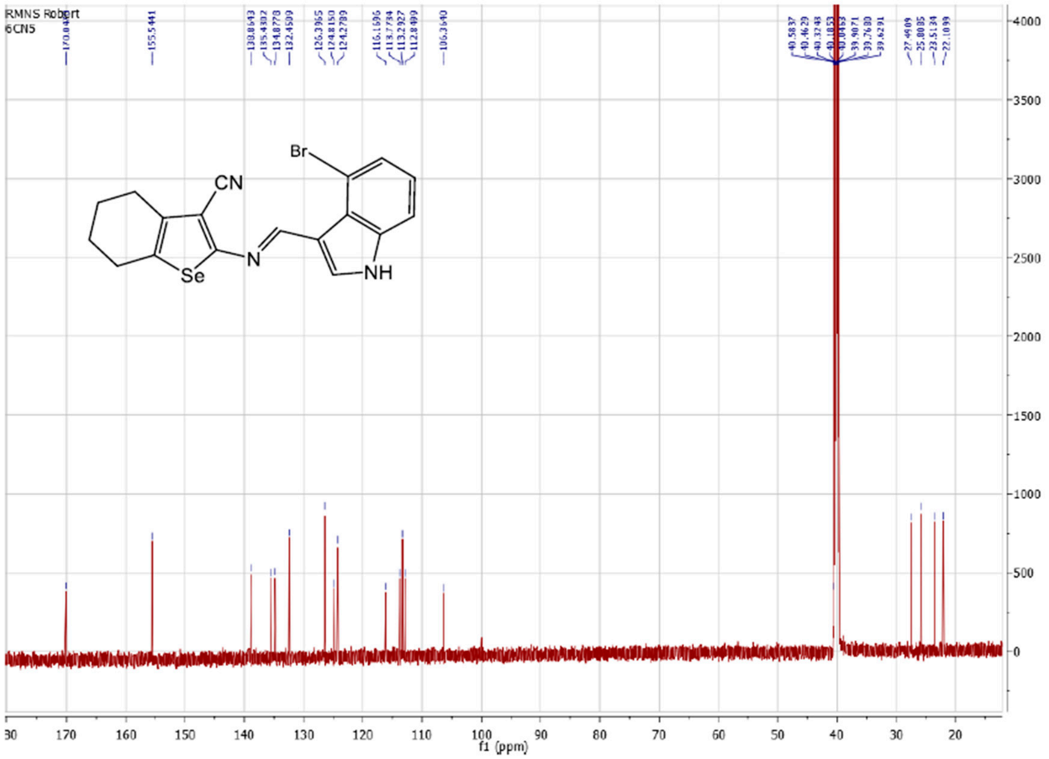

Figure S47: Mass spectra of 37

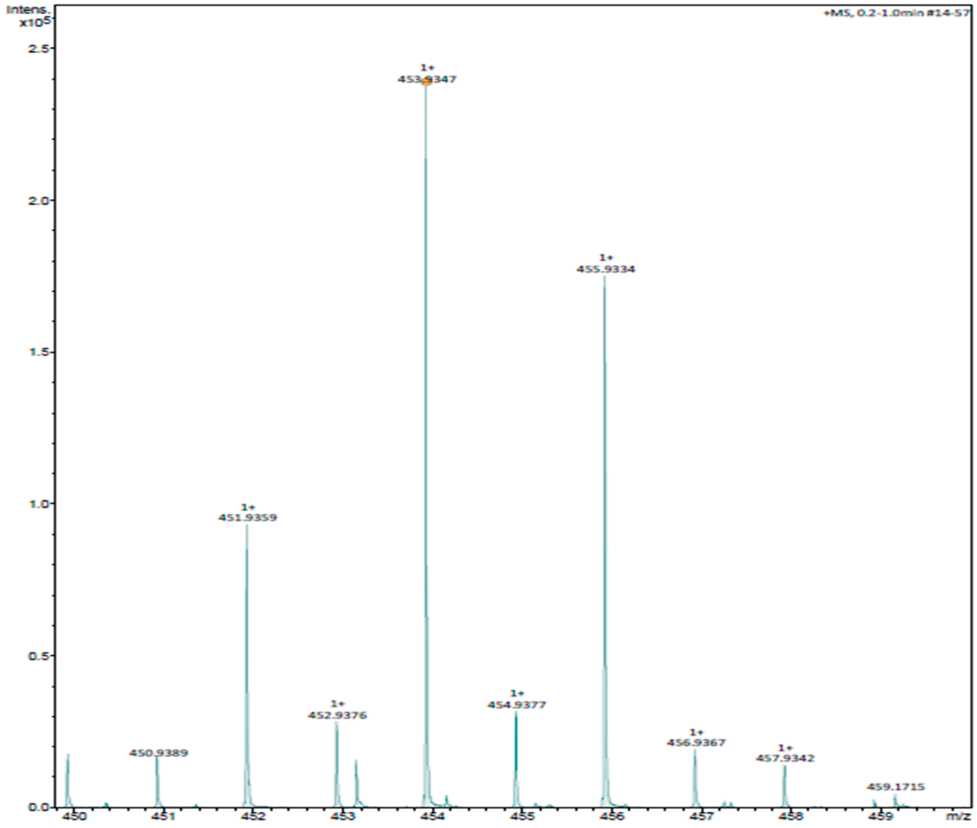

Figure S48: ^{1}H NMR full spectra of 38

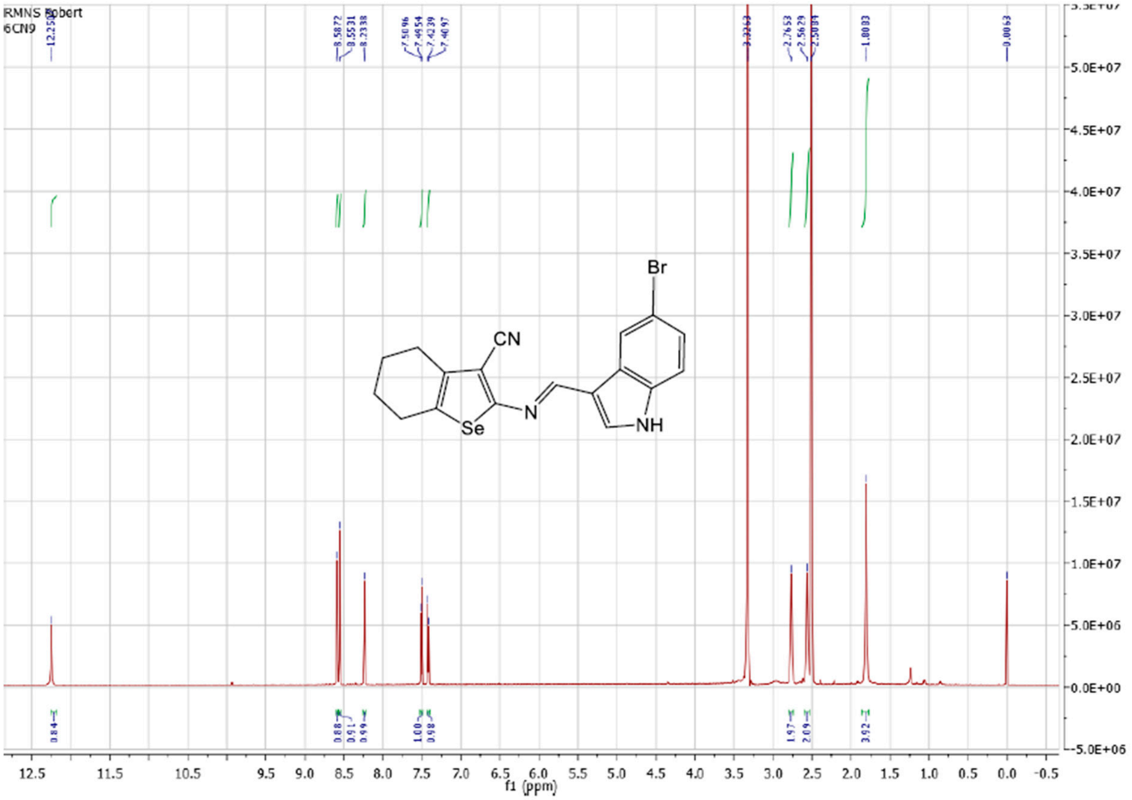

Figure S49: ^{1}H NMR expanded spectra of 38

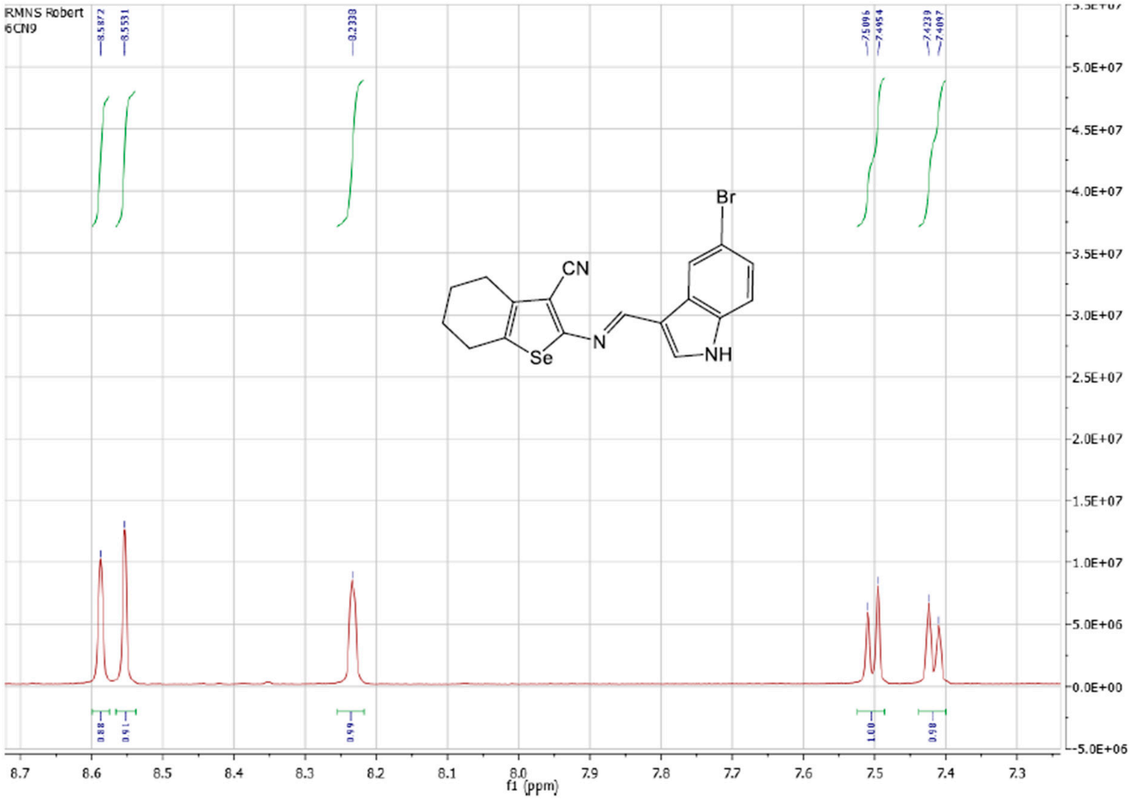

Figure S50: ¹³C NMR spectra of 38

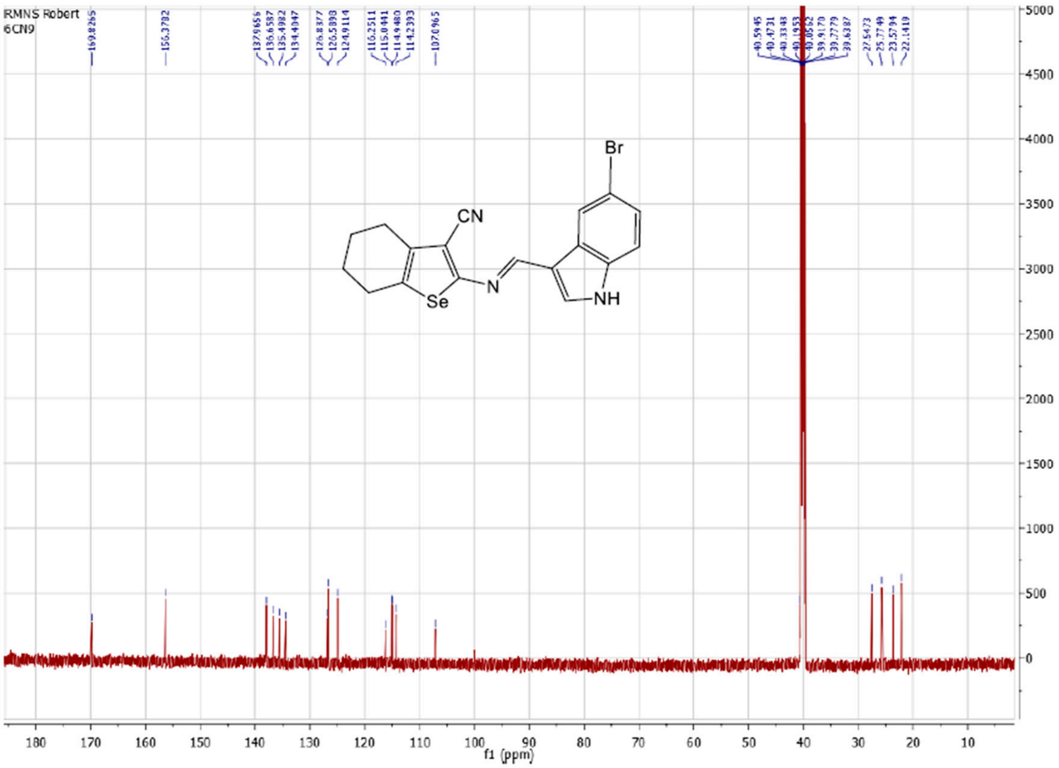

Figure S51: Mass spectra of 38

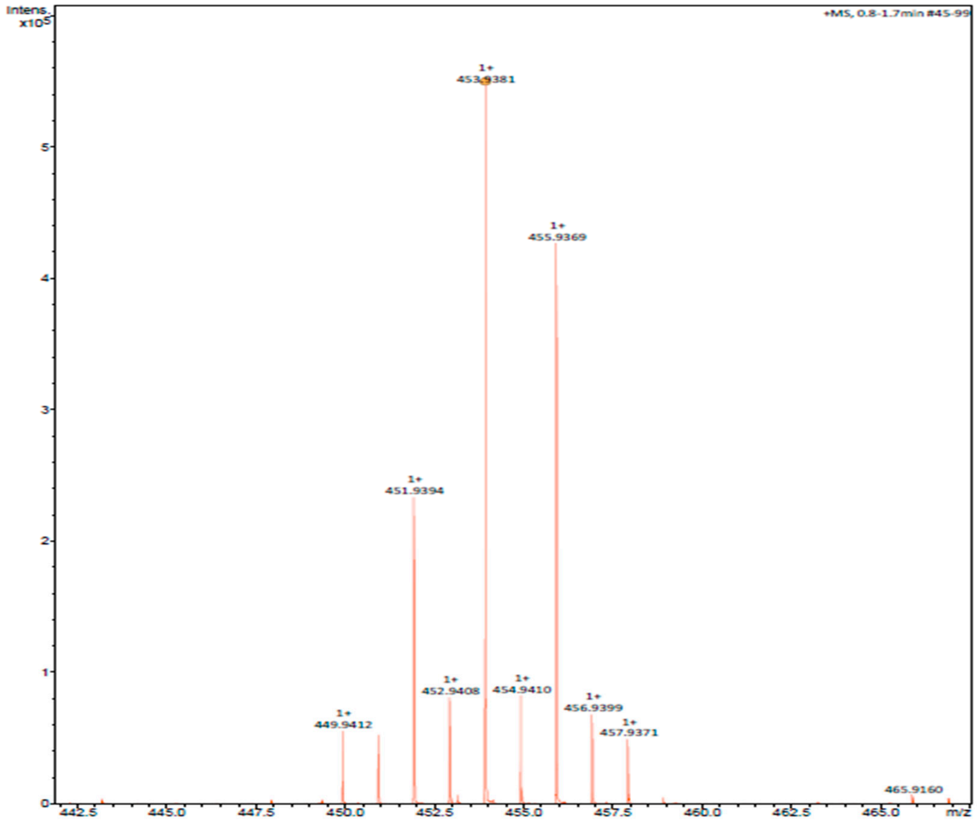

Figure S52: ^{1}H NMR full spectra of 39

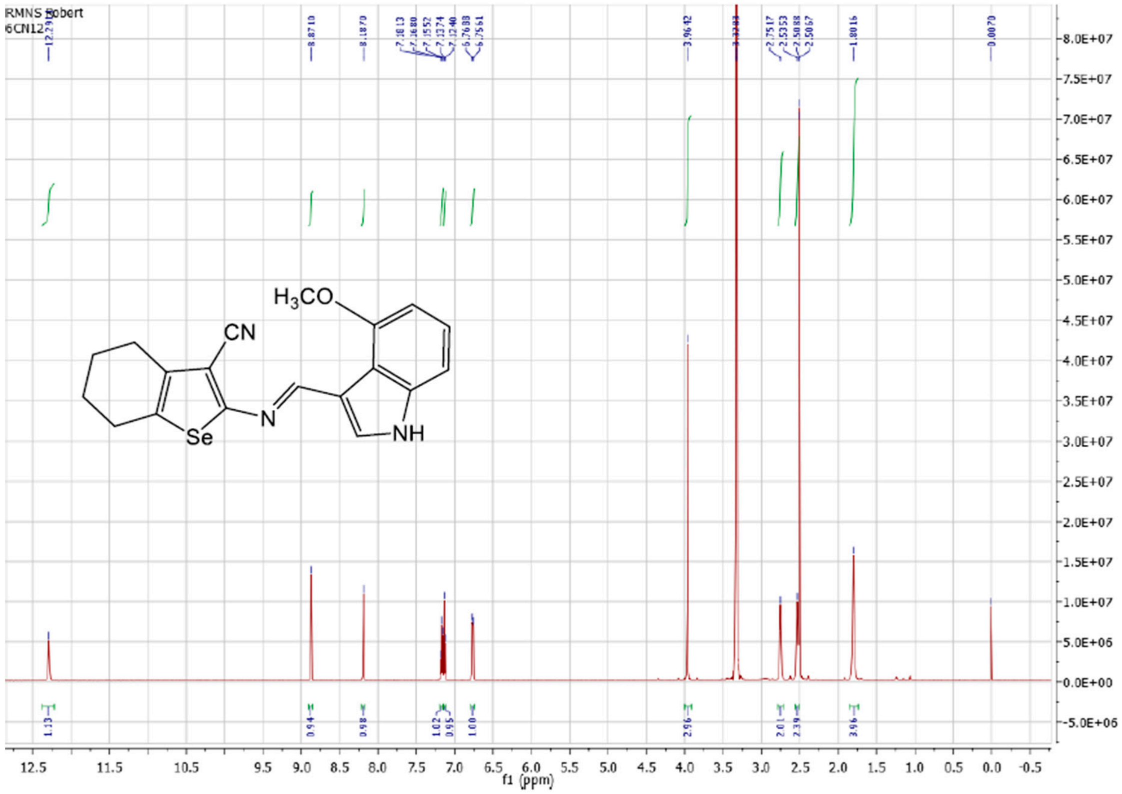

Figure S53: ^{1}H NMR expanded spectra of 39

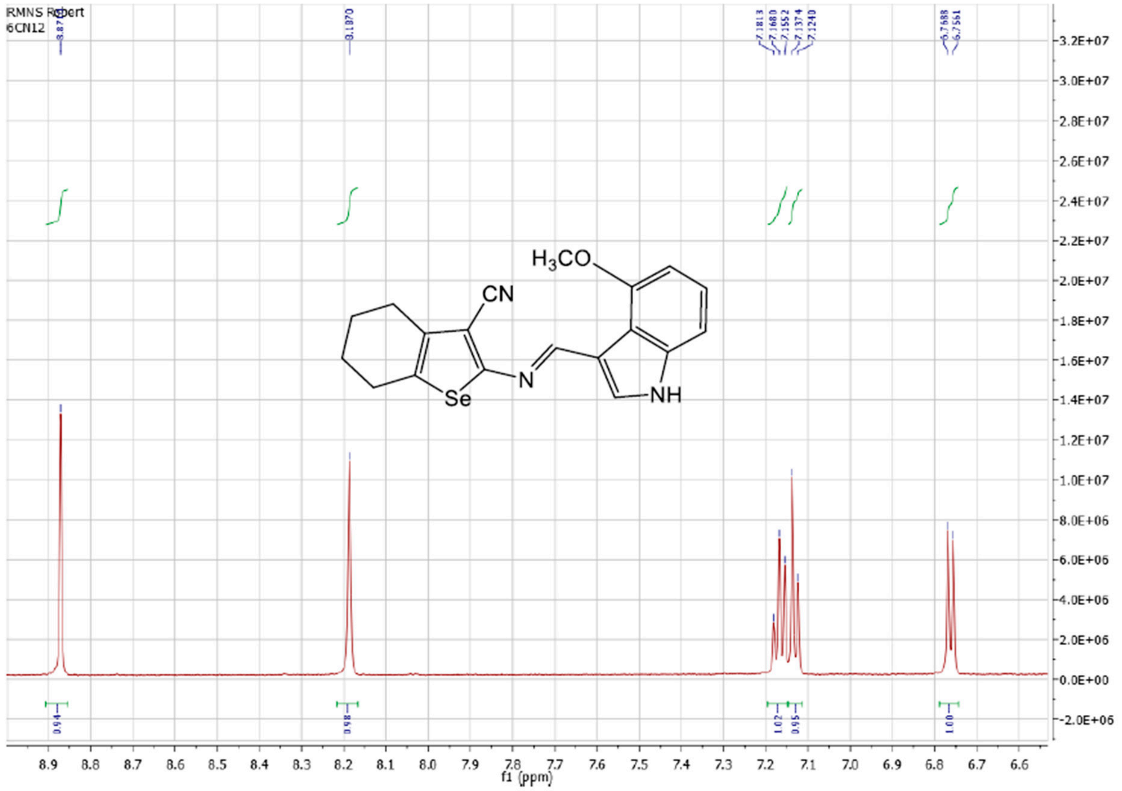

Figure S54: ¹³C NMR spectra of 39

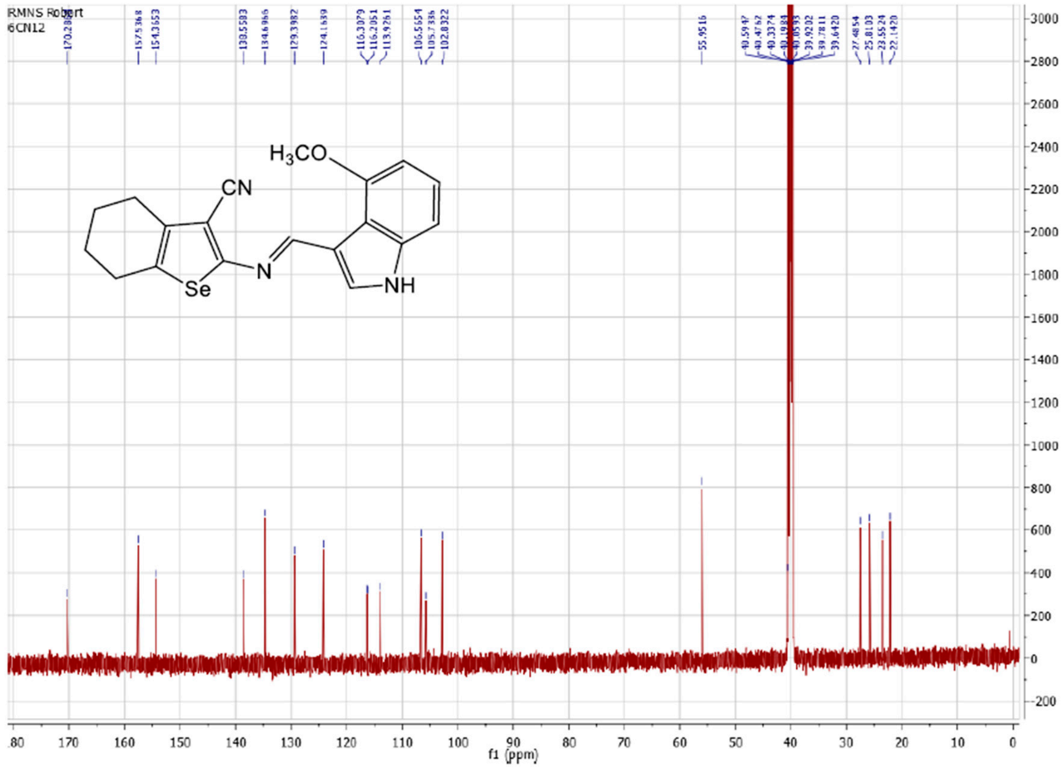

Figure S55: Mass spectra of 39

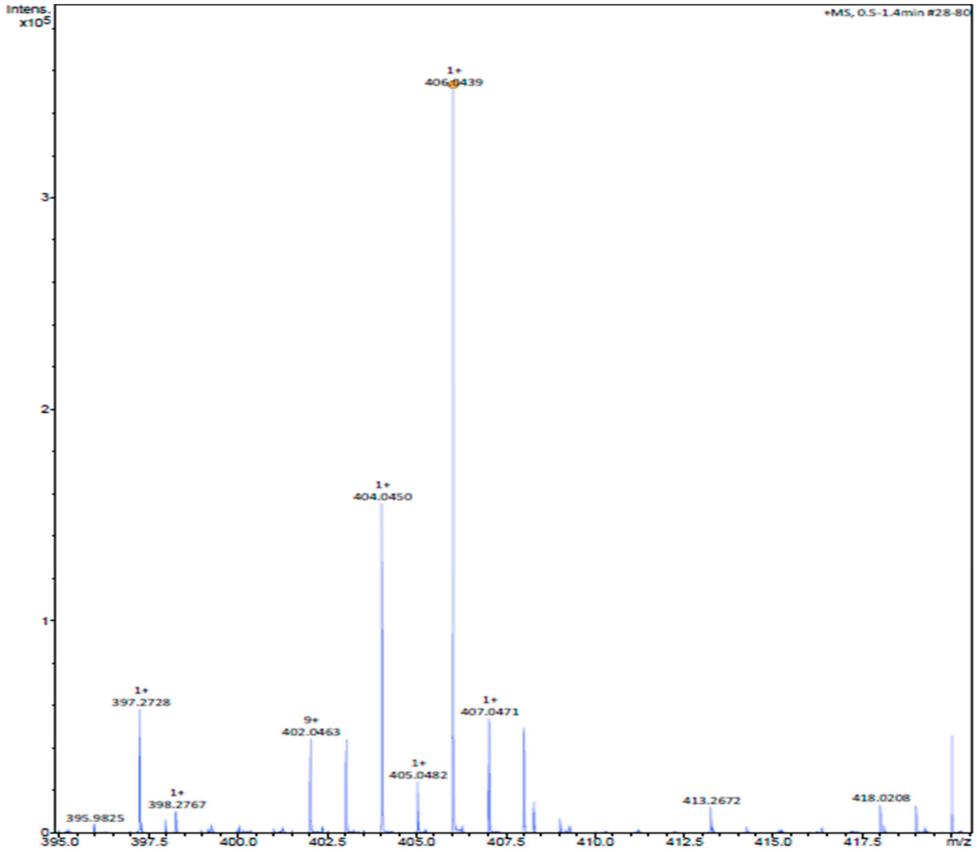

Figure S56: ^{1}H NMR full spectra of 40

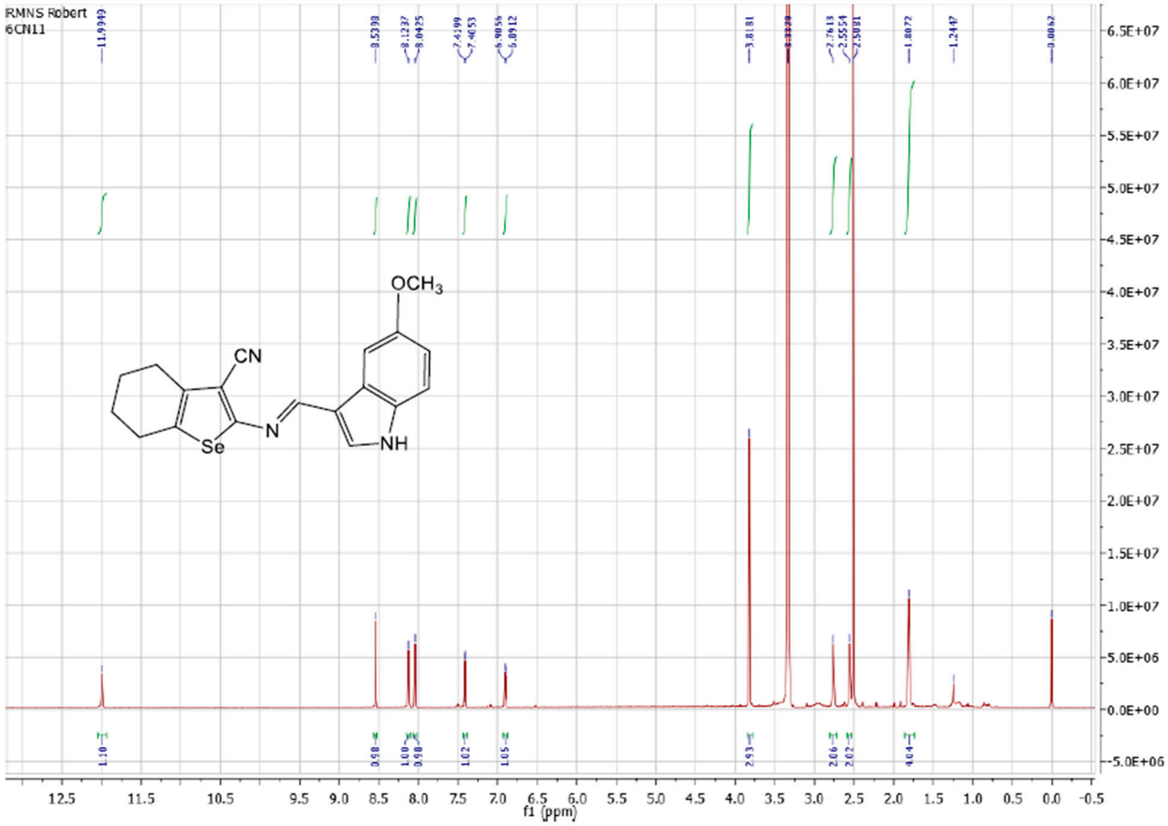

Figure S57: ^{1}H NMR expanded spectra of 40

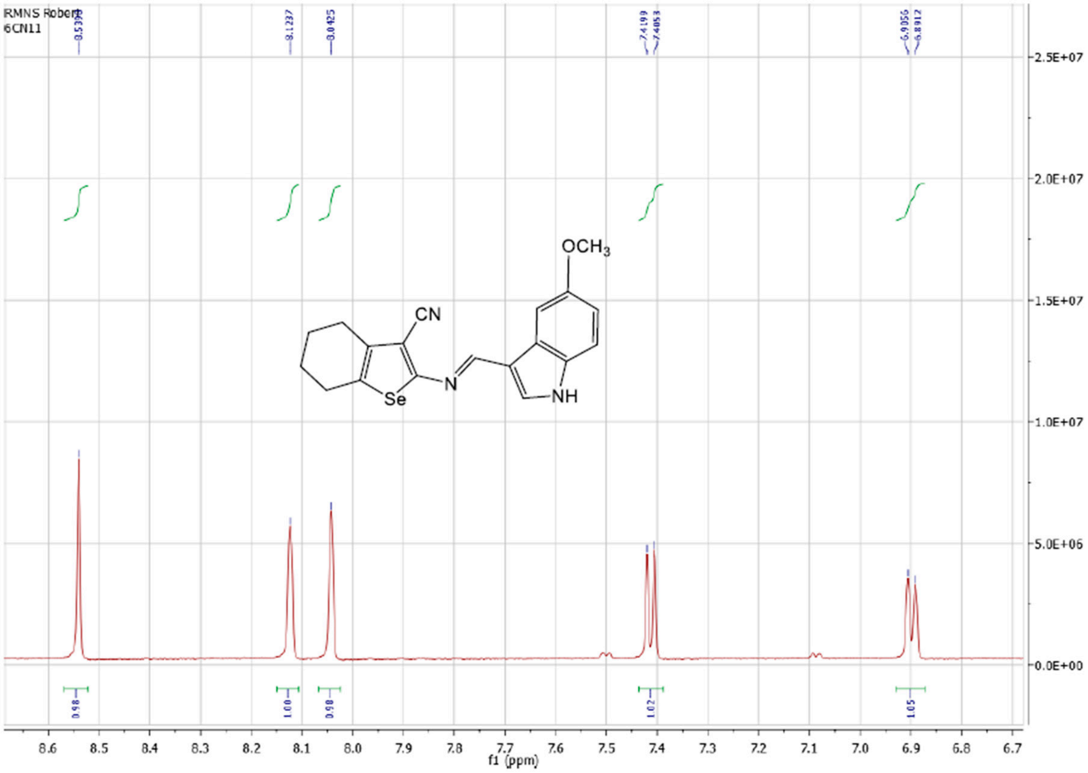

Figure S58: ^{13}C NMR spectra of 40

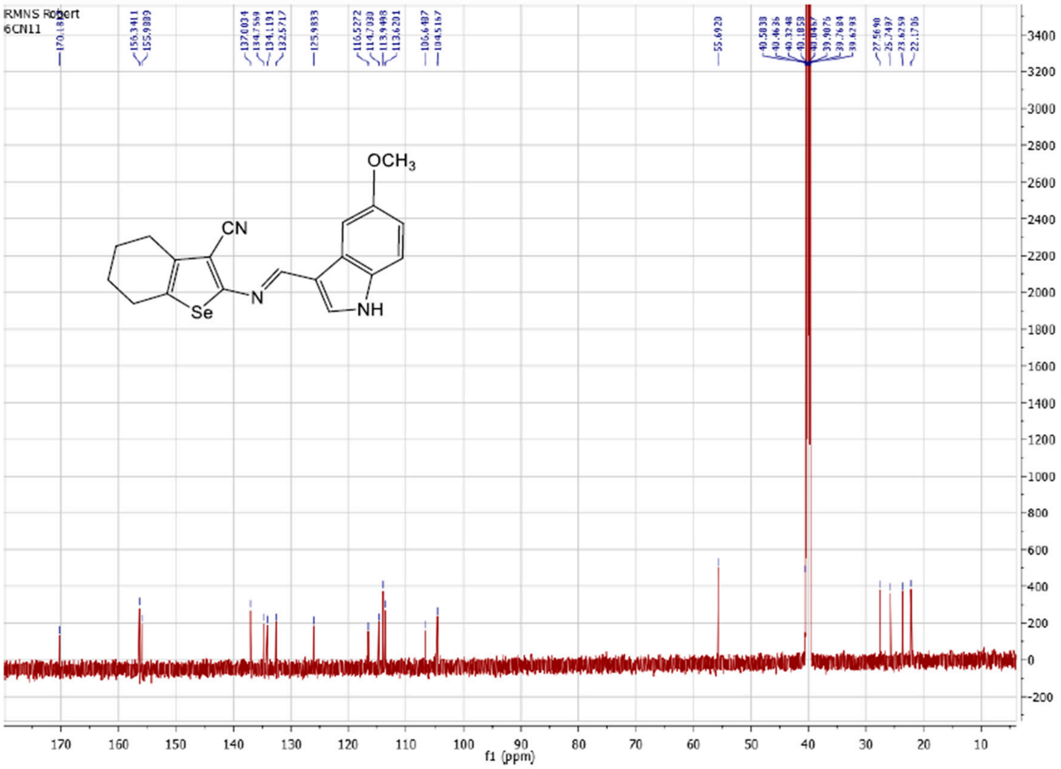

Figure S59: Mass spectra of 40

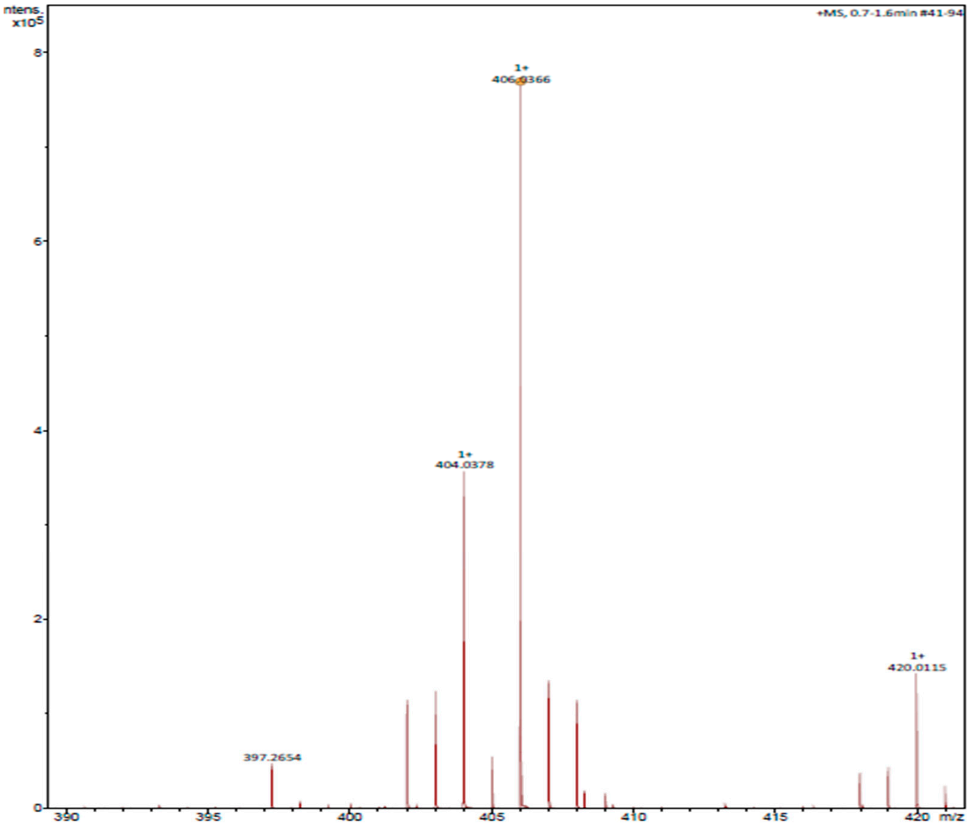

Figure S60. Chromatogram of 10 ug/mL of SB-44 before (top) and after (middle and bottom) chemical hydrolysis. Middle and bottom chromatograms are the aminothiophene and indole products, respectively.

SB44

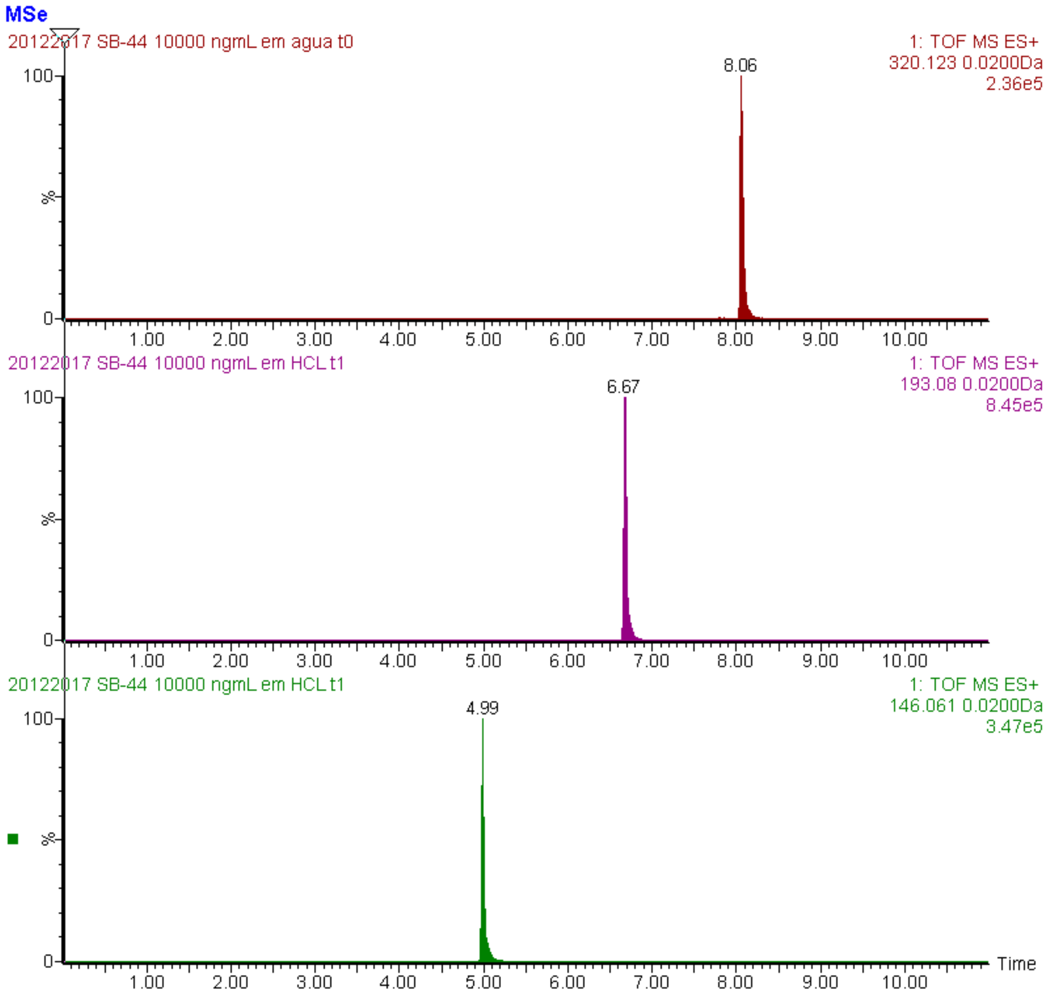

Figure S61. Spectra of SB-44 products after chemical hydrolysis. A and B show the aminothiophene hydrolysis product at low energy with the parent ion (m/z 193) in A and its product ions at high energy in B. C and D show the indole hydrolisis product at low energy with the parent ion (m/z 146) in C and its product ions at high energy in D.

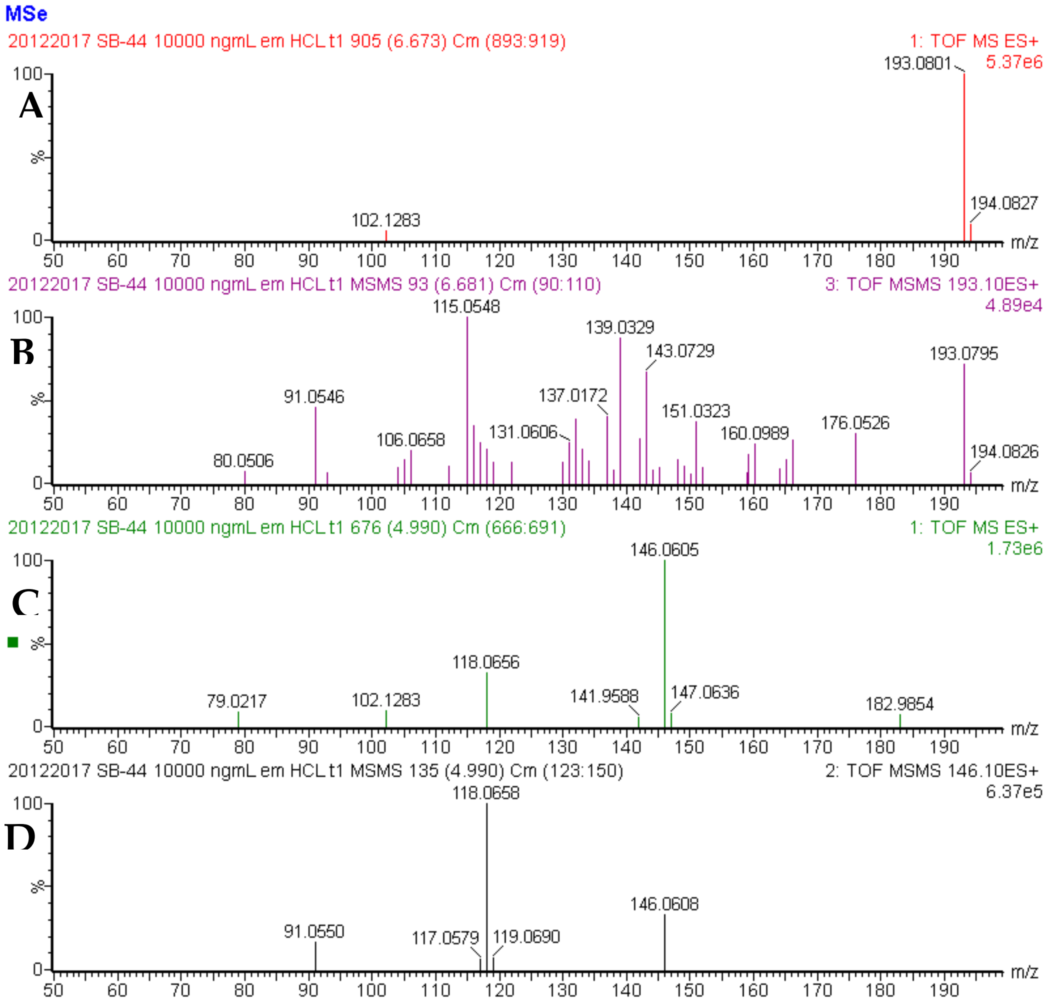

Figure S62. Chromatogram of 10 ug/mL of SB-83 before (top) and after (middle and bottom) chemical hydrolysis. Middle and bottom chromatograms are the aminothiophene and indole products, respectively.

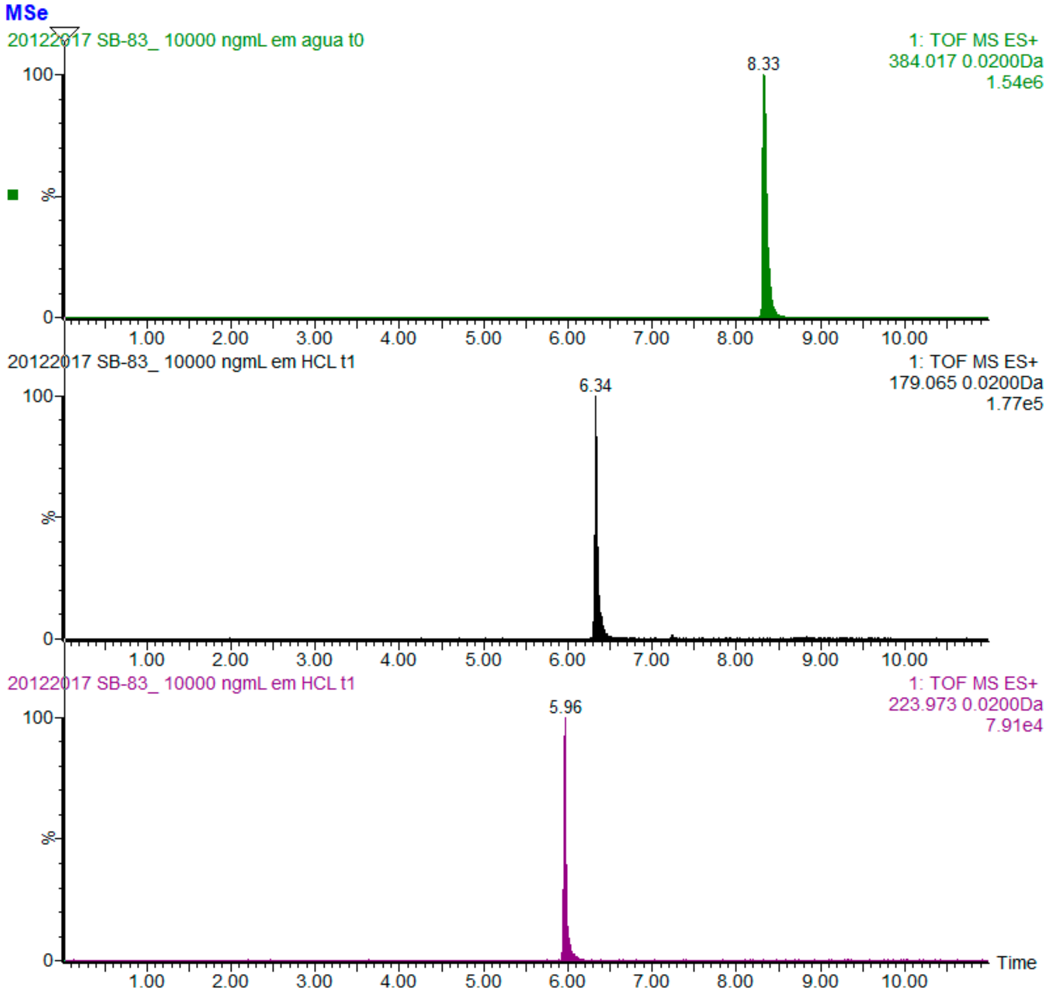

Figure S63. Spectra of SB-83 products after chemical hydrolysis. A and B show the aminothiophene hydrolisis product at low energy with the parent ion (m/z 179) in A and its product ions at high energy in B. C, D and E show the indole hydrolysis product at low energy with the parent ions of the two expected isotopes (m/z 224 and 226) in C and their respective product ions at high energy in D and E.

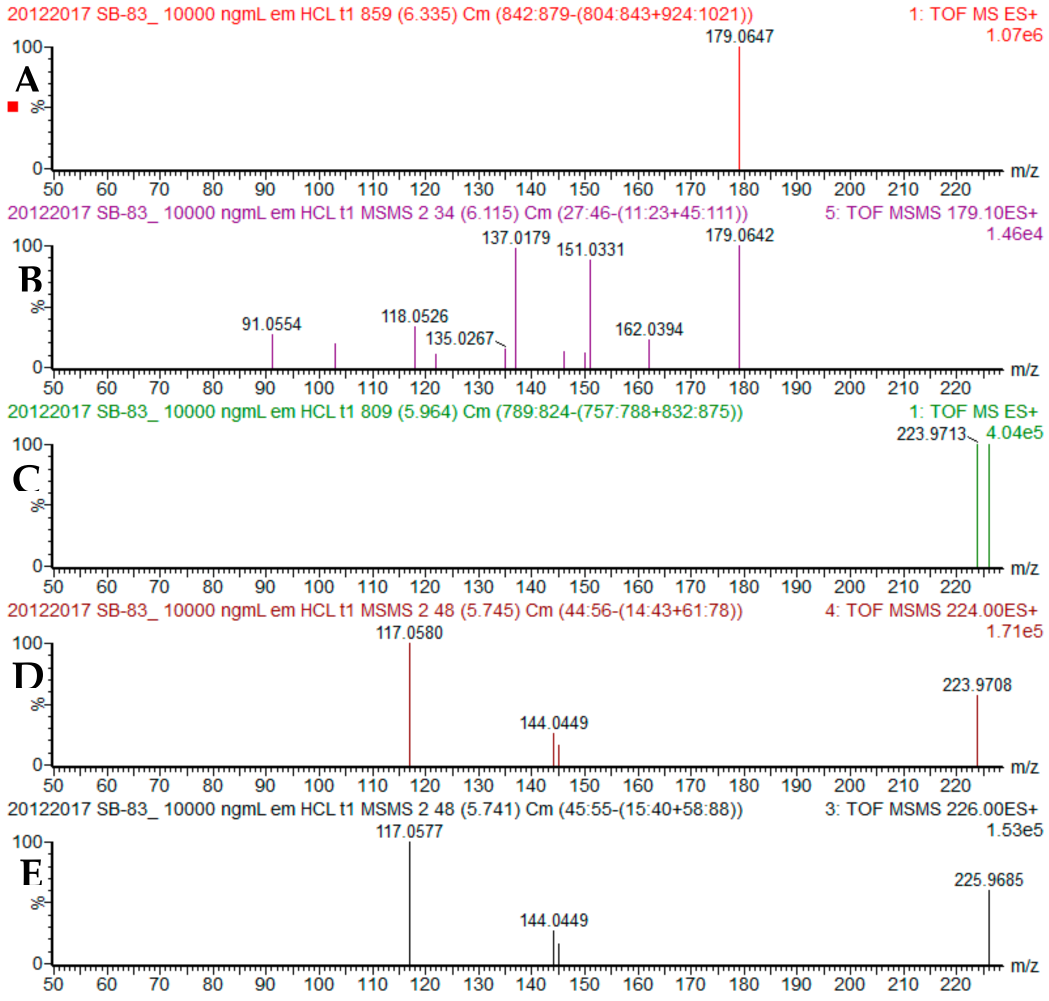

Figure S64. Chromatogram of 10 ug/mL of SB-200 before (top) and after (middle and bottom) chemical hydrolysis. Middle and bottom chromatograms are the aminothiophene and indole products, respectively.

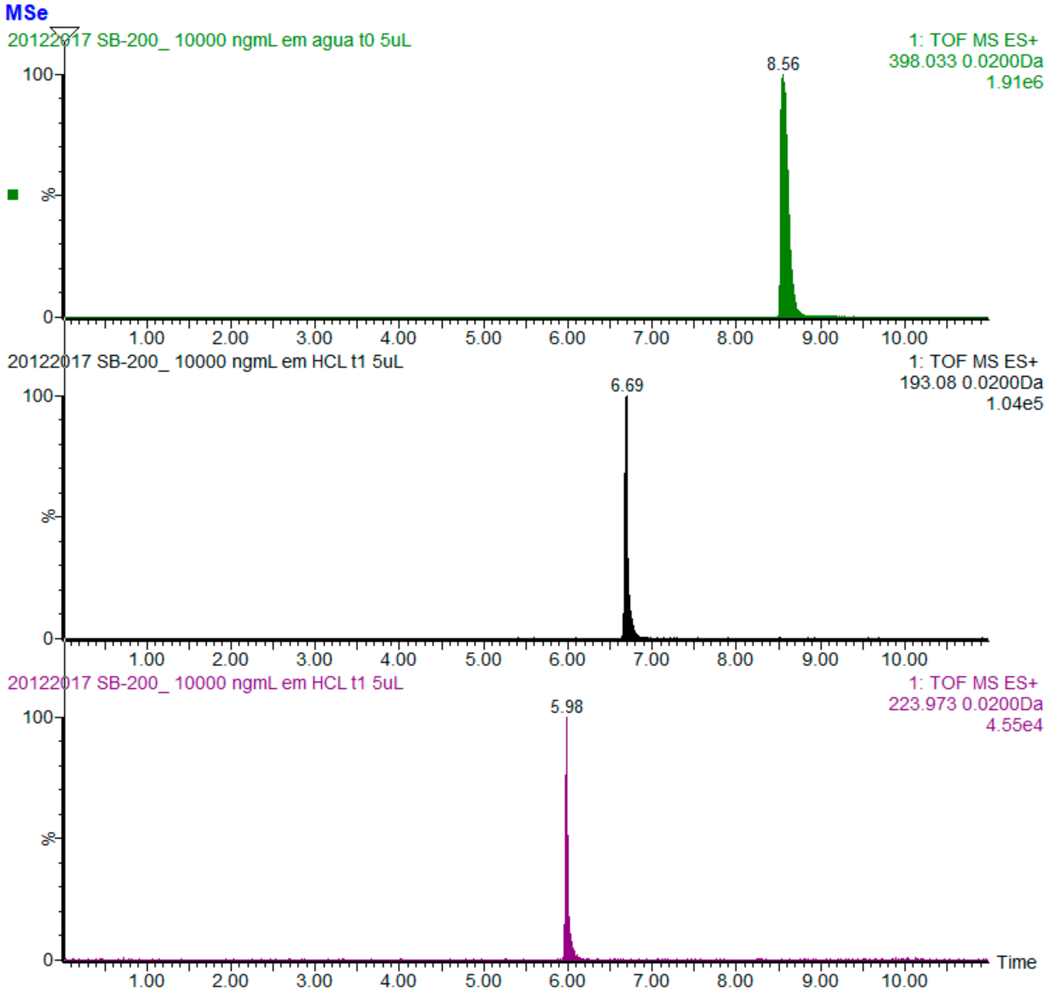

Figure S65. Spectra of SB-200 products after chemical hydrolysis. A and B show the aminothiophene hydrolisis product at low energy with the parent ion (m/z 193) in A and its product ions at high energy in B. C, D and E show the indole hydrolysis product at low energy with the parent ions of the two expected isotopes (m/z 224 and 226) in C and their respective product ions at high energy in D and E.

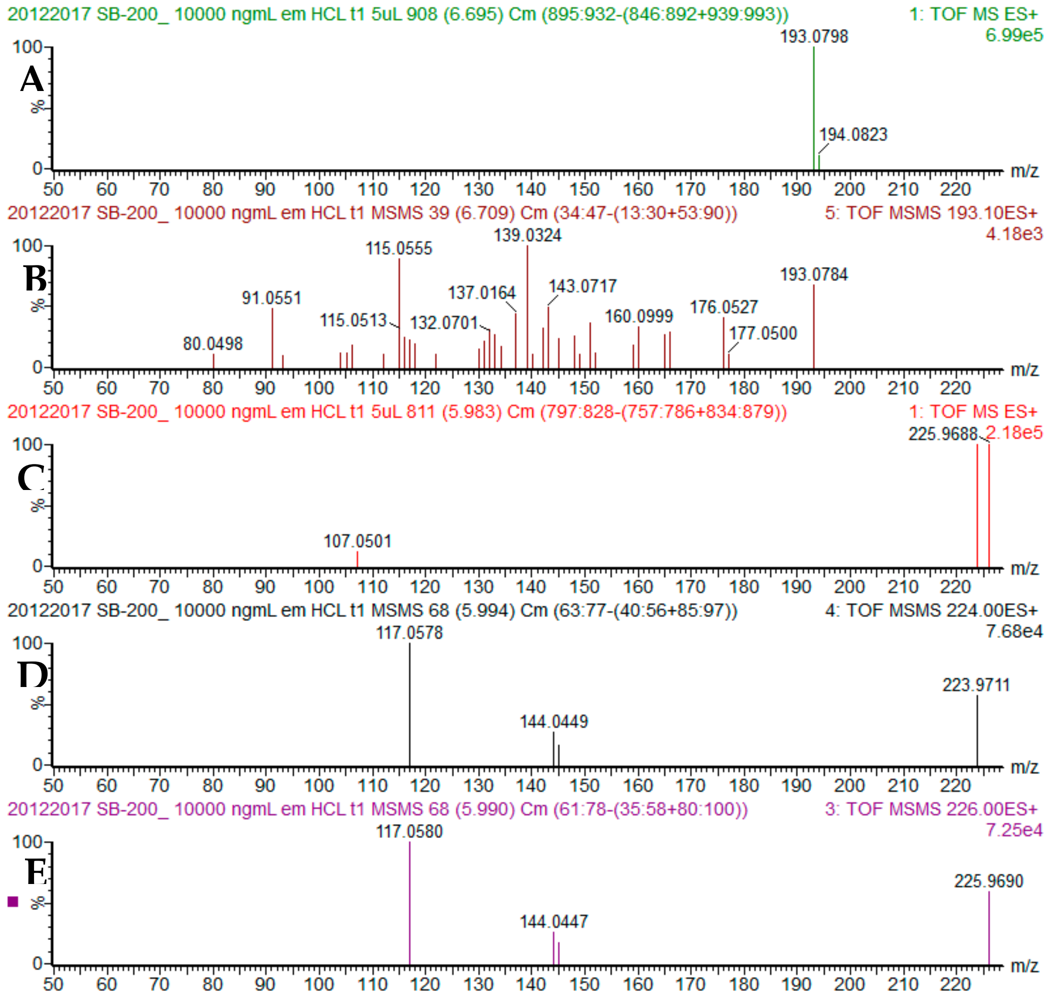

Figure S66. Chromatogram of 10 ug/mL of TN8-1 (top) and after (middle and bottom) chemical hydrolysis. Middle and bottom chromatograms are the aminothiophene and indole products, respectively.

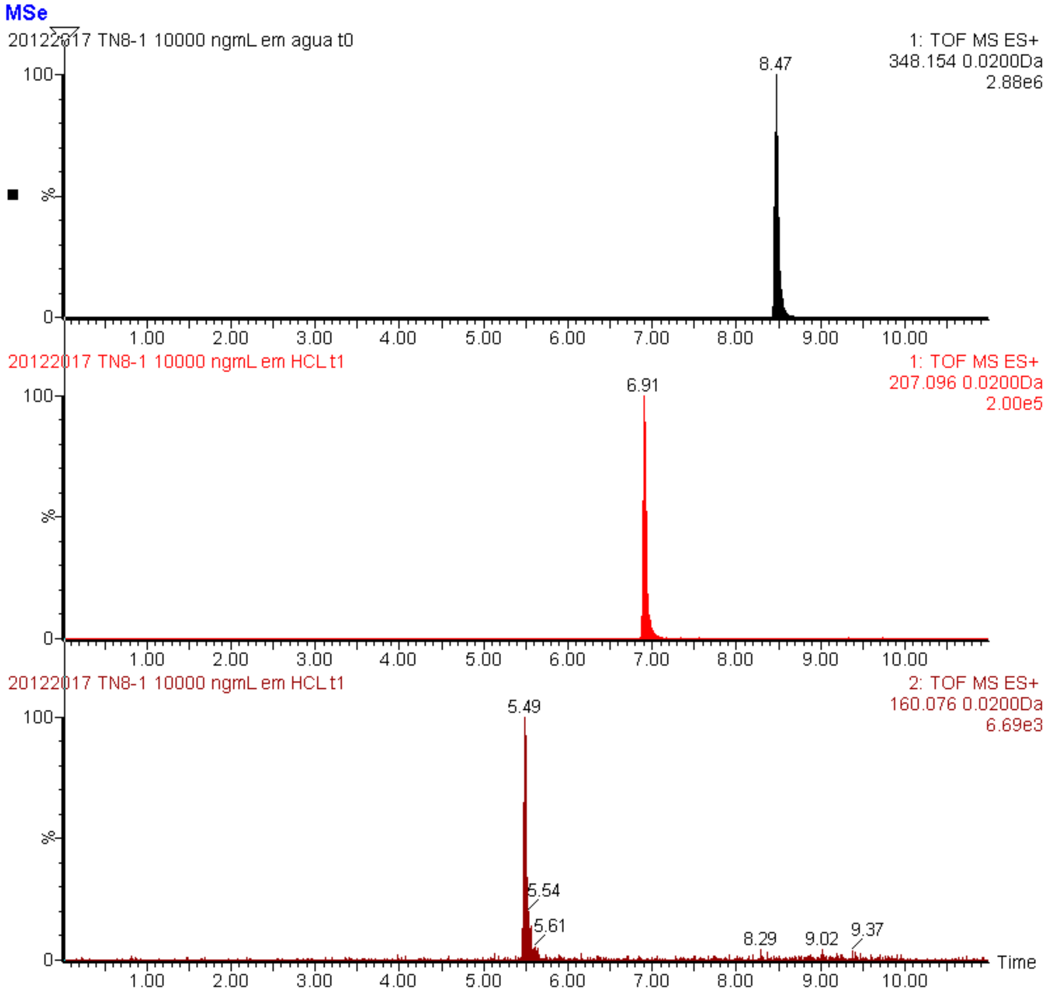

Figure S67. Spectra of TN8-1 products after chemical hydrolysis. A and B show the indole hydrolysis product at low energy with the parent ion (m/z 160) in A and its product ions at high energy in B. C and D show the aminothiophene hydrolysis product at low energy with the parent ion (m/z 207) in C and its product ions at high energy in D.

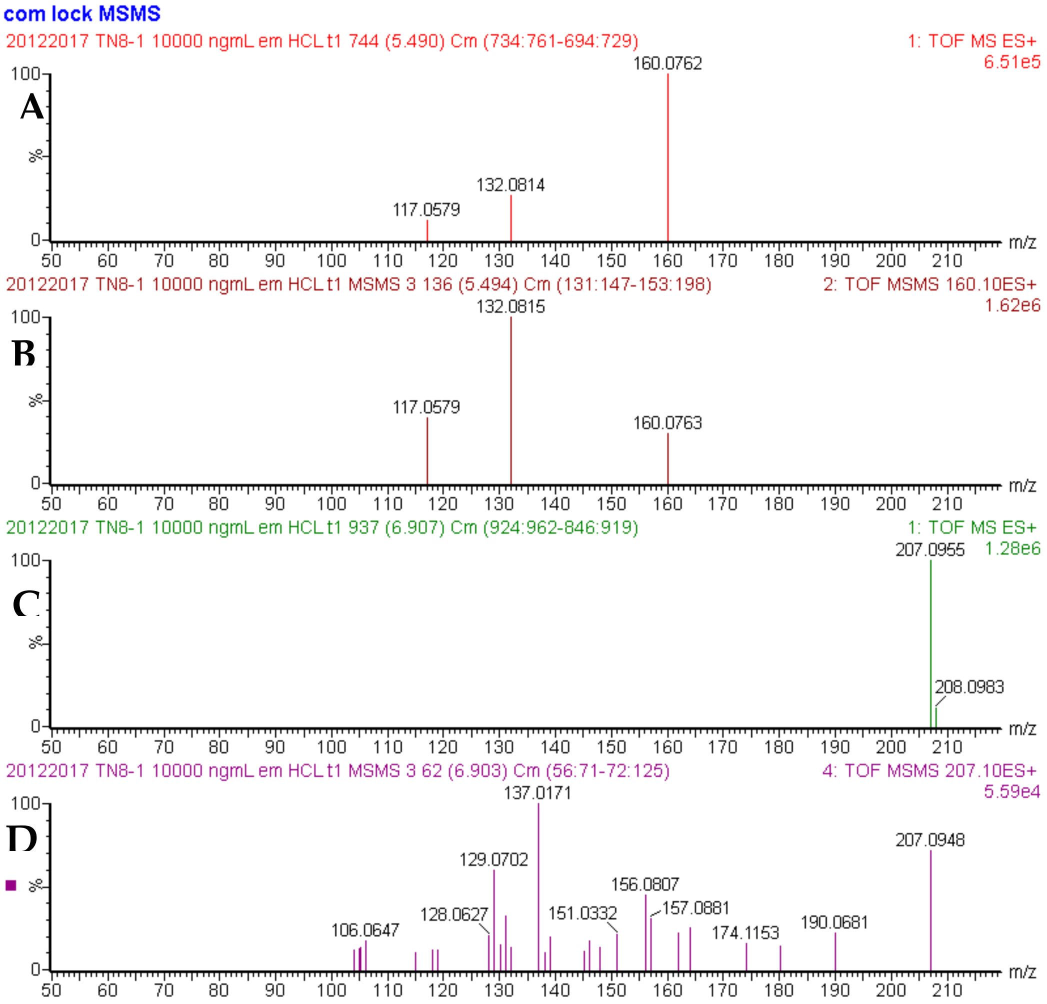

Figure S68. Chromatogram of 10 ug/mL of TN8-2 (top) and after (middle and bottom) chemical hydrolysis. Middle and bottom chromatograms are the aminothiophene and indole products, respectively.

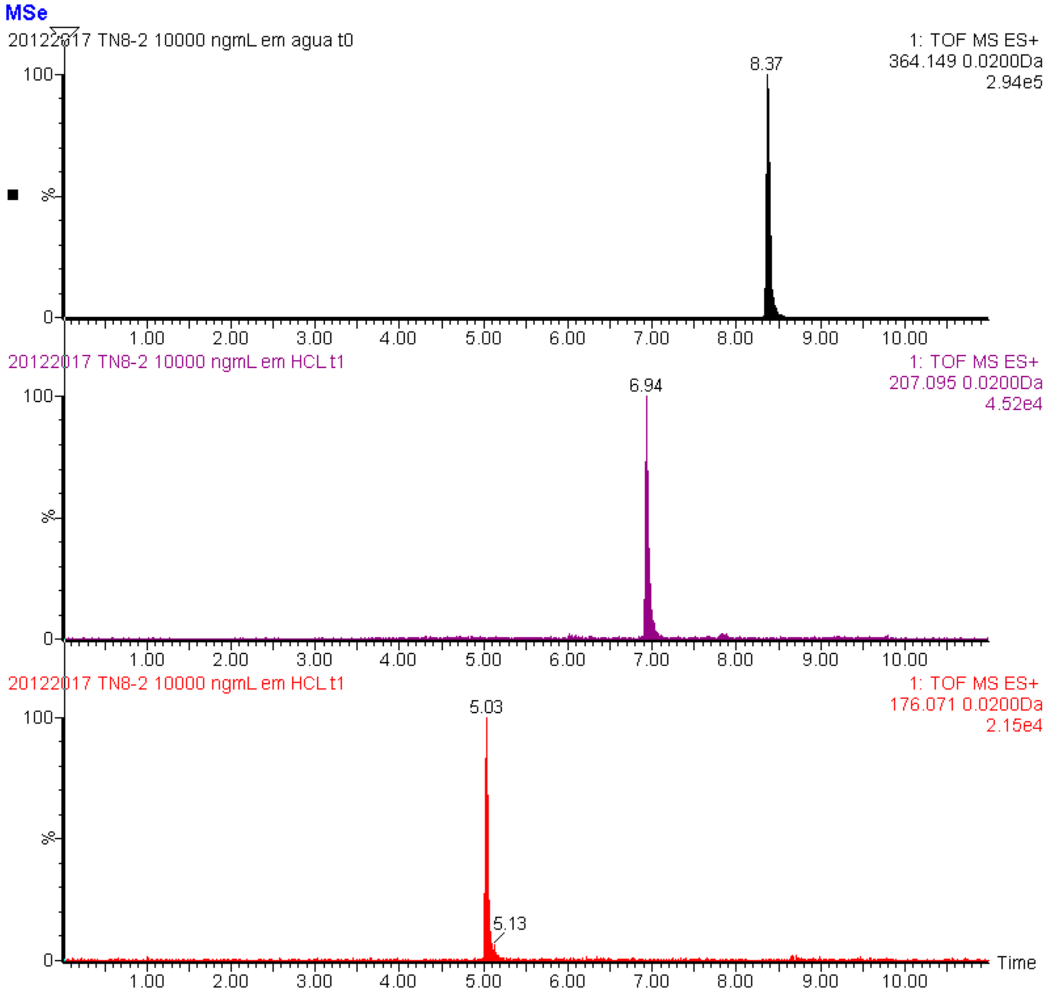

Figure S69. Spectra of TN8-2 products after chemical hydrolysis. A and B show the aminothiophene hydrolysis product at low energy with the parent ion (m/z 207) in A and its product ions at high energy in B. C and D show the indole hydrolysis product at low energy with the parent ion (m/z 176) in C and its product ions at high energy in D.

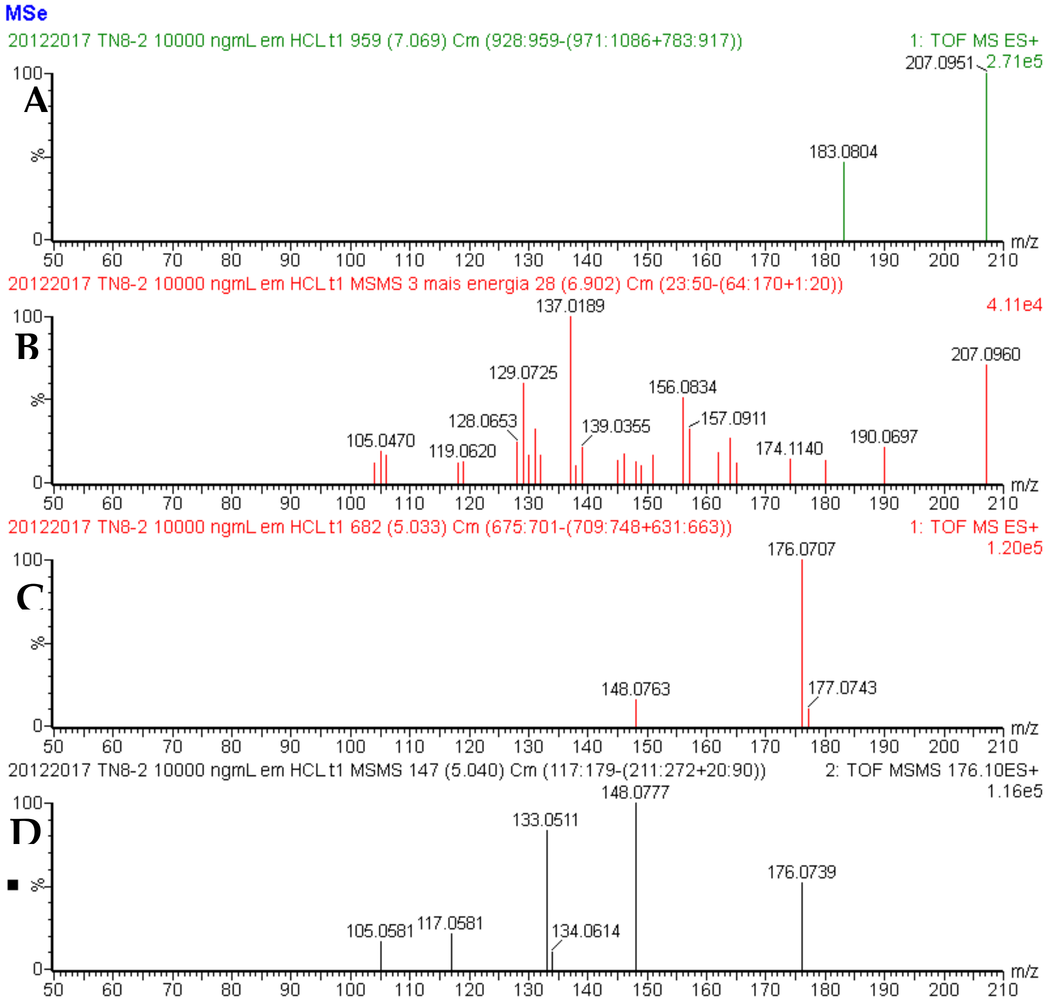

Table S1. Products from the aminothiophene derivatives using ultraperformance LC coupled to hybrid quadrupole-time of flight after chemical hydrolysis.

Product	M+H (m/z)	Erro (ppm)	RT (min)
Indole_SB44	146.0605	3.1	5.0
Aminothiophene_SB44-SB200	193.0798	2.1	6.7
Indole_SB83-SB200	223.9708	1.1	6.0
Aminothiophene_SB83	179.0647	5.3	6.3
Indole_TN8-1	160.0762	3.2	5.5
Aminothiophene_TN8-1-TN8-2	207.0951	0.3	6.9
Indole_TN8-2	176.0707	0.5	5.0
